# Optimal HIV testing strategies for South Africa: a model-based evaluation of population-level impact and cost-effectiveness

**DOI:** 10.1038/s41598-019-49109-w

**Published:** 2019-09-02

**Authors:** Leigh F. Johnson, Craig van Rensburg, Caroline Govathson, Gesine Meyer-Rath

**Affiliations:** 1 Centre for Infectious Disease Epidemiology and Research, University of Cape Town, Cape Town, South Africa; 2 Health Economics and Epidemiology Research Office, University of the Witwatersrand, Johannesburg, South Africa; 3 Department of Medicine, Faculty of Health Sciences, University of the Witwatersrand, Johannesburg, South Africa; 4 Department of Global Health, Boston University School of Public Health, Boston, USA

**Keywords:** HIV infections, Diagnosis, Applied mathematics

## Abstract

Although many African countries have achieved high levels of HIV diagnosis, funding constraints have necessitated greater focus on more efficient testing approaches. We compared the impact and cost-effectiveness of several potential new testing strategies in South Africa, and assessed the prospects of achieving the UNAIDS target of 95% of HIV-positive adults diagnosed by 2030. We developed a mathematical model to evaluate the potential impact of home-based testing, mobile testing, assisted partner notification, testing in schools and workplaces, and testing of female sex workers (FSWs), men who have sex with men (MSM), family planning clinic attenders and partners of pregnant women. In the absence of new testing strategies, the diagnosed fraction is expected to increase from 90.6% in 2020 to 93.8% by 2030. Home-based testing combined with self-testing would have the greatest impact, increasing the fraction diagnosed to 96.5% by 2030, and would be highly cost-effective compared to currently funded HIV interventions, with a cost per life year saved (LYS) of $394. Testing in FSWs and assisted partner notification would be cost-saving; the cost per LYS would also be low in the case of testing MSM ($20/LYS) and self-testing by partners of pregnant women ($130/LYS).

## Introduction

HIV testing is a critical first step in ensuring that HIV-positive individuals are diagnosed and treated, which in turn is critical to reducing HIV incidence. Globally it is estimated that in 2017, 75% of all HIV-positive individuals had been diagnosed positive^1^. The UNAIDS target is to increase this fraction diagnosed to 90% by 2020 and to 95% by 2030^2^. In many sub-Saharan African countries, where the burden of HIV is most severe, high levels of HIV diagnosis have already been achieved^3–5^. However, challenges remain, with the fraction of HIV-positive individuals who are diagnosed being relatively low in men, young adults and key populations such as sex workers and men who have sex with men (MSM)^5–10^. In addition, funding constraints have led to increased emphasis on efficiency, which is often assessed in terms of testing ‘yield’ (number of new diagnoses per test performed)^10,11^.

HIV testing services have historically been located in health facilities offering services such as antenatal care (ANC), sexually transmitted infection (STI) treatment, medical male circumcision (MMC) and treatment for HIV-related opportunistic infections (OIs). In recent years there have been several innovations to expand HIV testing coverage and to reach sub-populations that have previously had low rates of HIV testing. These include community-based testing strategies such as home-based testing and mobile testing, the latter being particularly effective in reaching men and key populations^12–14^. Recent studies have also demonstrated the value of adopting more active approaches to partner notification, with health workers contacting partners of newly-diagnosed individuals to encourage HIV testing^15^. Self-testing has been another major innovation^16^, with studies showing that the supply of self-testing kits leads to increased uptake of testing by partners of pregnant women^17^, sex workers^18,19^ and individuals in household settings^20,21^.

Given the growing menu of options for delivering HIV testing, and given the growing pressure on HIV programmes to deliver HIV testing more efficiently, there is a need for mathematical modelling to identify which strategies are likely to have the greatest population-level impact on levels of diagnosis, and which strategies will be most cost-effective. Few mathematical modelling studies have been conducted to evaluate the relative merits of different HIV testing strategies. A recent study by Korenromp and Stover^22^ modelled the need for new HIV testing strategies to reach the 90% target in four countries (Mozambique, Senegal, Nigeria and Bolivia), comparing 12 possible HIV testing modalities. Although many other models have evaluated individual HIV testing strategies, few have compared a broad range of different HIV testing strategies^23–28^, and most have assessed cost-effectiveness relative to internationally-accepted benchmarks (such as three times gross domestic product (GDP) per life year saved) rather than against locally-established ‘willingness to pay’ criteria. This makes it difficult for policymakers to determine which HIV testing interventions represent the best ‘value for money’ relative to currently-funded programmes.

This study aims to evaluate the potential impact and cost-effectiveness of a number of potential new HIV testing strategies in South Africa, the country with the largest number of HIV infections globally. In this setting, high levels of uptake and coverage have already been achieved for many HIV interventions, with the HIV-diagnosed fraction estimated at over 80% in recent years^29^. In this context of high baseline levels of intervention uptake, it is relatively difficult to identify new interventions that represent value for money, and the ‘willingness to pay’ threshold of the South African government (the main funder of the country’s HIV response) has been estimated at $547–842 per life year saved, substantially lower than the South African GDP per capita of around $6 000^30^.

## Methods

This analysis is based on the MicroCOSM model, an agent-based model that was developed to simulate HIV and other STIs in South Africa^31^. A detailed description of the model is provided elsewhere^32^. Briefly, the model simulates a nationally-representative sample of individuals, with the initial simulated population size set to 20 000 at the start of the simulation (in 1985). Each simulated individual is randomly assigned a date of birth, sex and race, and individuals are tracked over time, with the model randomly assigning to each individual a series of demographic events (birth, death and migration between urban and rural areas), educational events (entering school and passing, failing or dropping out of school at the end of each year), relationship events (acquiring new partners, marrying partners, ending relationships and engaging in casual or commercial sex), health events (acquisition of HIV and other STIs) and healthcare utilization events (adoption or discontinuation of hormonal contraception, condoms, pre-exposure prophylaxis (PrEP), antiretroviral treatment (ART) and MMC). The model also simulates incarceration and unemployment. The model allows for heterogeneity between individuals in sexual preference, propensity for concurrent partnerships and commercial sex, and willingness to use condoms. Individuals who form new partnerships are assumed to select their partner from within the simulated population, with sexual mixing patterns being assumed to be highly assortative with respect to age, race, educational attainment, risk group and location. Model assumptions about HIV transmission probabilities per sex act are calibrated such that the model matches HIV prevalence data from three national household surveys (conducted in 2005, 2008 and 2012^33–35^) and national antenatal surveys conducted over the 1997–2015 period^36^, as well as surveys of HIV prevalence in sex workers and MSM. Although the model simulates both sexual and mother-to-child transmission of HIV, the model does not currently simulate HIV testing in children, and this analysis is therefore limited to testing in adults.

**Figure 1:**
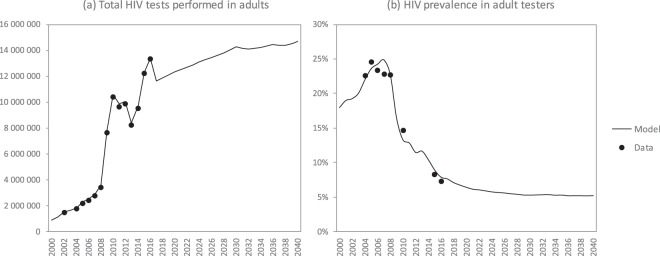
Trends in adult HIV testing levels and yields. Model projections are shown for the ‘baseline’ scenario, i.e. assuming no changes to existing HIV testing modalities. Data sources include routine HIV testing in public health facilities, HIV tests for life insurance purposes, HIV tests conducted by medical schemes and HIV tests as part of workplace testing programmes and other private sector programmes, as described elsewhere^37^.

The model simulates eight different HIV testing modalities that have already been introduced in South Africa: ‘general’ HIV testing (self-initiated testing, testing for insurance purposes and provider-initiated testing not included in other modalities), ANC testing, testing in OI patients, testing partners of newly-diagnosed individuals (‘passive referral’), STI patient testing, testing men seeking MMC, testing in prisons and testing in individuals receiving PrEP^32^. Key HIV testing assumptions are summarized in Table 1, and a more detailed description of these ‘baseline testing modalities’ is provided in the supplementary materials (section 2.1). Assumptions about historic rates of HIV testing have been set with reference to reported levels of HIV testing in antenatal clinics, STI clinics, tuberculosis patients (as a proxy for patients with OIs), and prisons, and the model has been calibrated to match the total annual numbers of HIV tests performed in South Africa (private and public sector combined^37^) as well as the fraction of tests that are positive over the 2002–2016 period (Fig. 1). All men who seek MMC and all PrEP recipients are assumed to receive HIV testing prior to MMC/PrEP initiation. The rate of ‘general’ HIV testing is assumed to depend on age, sex, educational attainment, HIV testing history and calendar period. For all HIV testing modalities (with the exception of those associated with MMC and PrEP), it is assumed that previously-diagnosed individuals can get retested, although the rate of testing is assumed to be reduced by a factor of 50% in previously-diagnosed ART-naïve individuals and by 85% in ART-experienced individuals^37^. After diagnosis, HIV-positive individuals are assumed to disclose their HIV status to their partner(s) with a probability that depends on their sex and relationship type. If disclosure occurs, there is a probability that the partner will seek HIV testing (passive referral). Female disclosure of an HIV-positive status is assumed to be associated with increased risk of union dissolution. If the relationship continues and the HIV status of the other partner is negative or unknown, there is assumed to be an increased odds of consistent condom use. Individuals who test positive are assumed to start ART with a probability that depends on the HIV testing modality, and the same probability is assumed to apply whether the individual is newly diagnosed or testing positive after a prior diagnosis^38^.

**Table 1:** HIV testing assumptions.

Parameter	Value	Range*	Source
RR of testing in previously-diagnosed ART-naïve	0.50	0.025–0.0975†	—
RR of testing in ART patients (relative to diagnosed ART-naïve)	0.36	0.07–0.74†	^54^
Probability that newly-diagnosed woman discloses her HIV status to a short-term partner	0.50	—	^55– 57^
OR for effect of marriage on probability of disclosure	2.50	—	^57– 59^
OR for effect of male sex on probability of disclosure	1.25	—	^58, 59^
Probability of referral for testing if disclosure occurs	0.42	—	^60^**
OR for effect of assisted partner notification on partner referral for HIV testing	3.00	1.79–4.52	^61, 62^
Fraction of adult population tested for each round of home-based testing	0.70	0.55–0.83	^12^
Ratio of male to female uptake of testing through home-based testing	0.67	—	^12^
Annual rate of testing in women attending FP clinics if HIV testing is integrated into FP	0.45	0.28–0.67	^63, 64^
Annual rate of HIV testing through mobile testing in communities with mobile testing services	0.055	0.023–0.101	^65, 66^
Relative rate of testing through mobile clinics in the presence of community mobilization	2.0	—	^67^
Increase in annual rate of testing in MSM if MSM-focused HIV testing is introduced	0.40	0.11–0.88	^39, 40^
Increase in annual rate of testing in FSWs if FSW-focused HIV testing is introduced	1.35	0.14–3.90	^68– 70^
Probability of test acceptance by sexually-experienced adolescents if offered testing in school	0.67 (M) 0.76 (F)	—	^71^
Relative rate of school testing in virgins	0.50	0.13–0.87	^72^
Fraction of employed population reachable through annual workplace testing programmes	0.30	0.03–0.70	^41^
Probability of testing through workplace testing if workplace testing is offered	0.22 (M) 0.28 (F)	—	^73^
Fraction of husbands of married pregnant women who get tested if given invitation letters	0.33	0.15–0.53	^74– 78^
OR for effect of marriage on probability of pregnant women’s partner getting tested	4.00	—	^76, 79, 80^
OR for effect of self-testing offer on uptake of testing	8.17	6.33–10.24	^16^
Probability of starting ART soon after diagnosis			
Diagnosed in ANC setting	0.93	—	^81^
Diagnosed in OI/TB clinic	0.78	—	^81^
Diagnosed in other clinic-based settings	0.40	—	^82^
RR of starting ART if diagnosed through community-based testing (relative to other clinic-based testing)	0.68	0.36–0.93	^18, 49– 51^
*Cost assumptions*			
Average health worker time (minutes) per facility-based test			
HIV-positive test	27.98	—	S2.6.4.1
HIV-negative test	21.63	—	S2.6.4.1
Cost per rapid test (first test) (USD)	0.52	—	S2.6.4.1
Cost per rapid test (second test) (USD)	0.54	—	S2.6.4.1
Cost per self-testing kit (USD)	2.40	—	S2.6.4.6
Cost per person year of ART (USD)	280.07	—	^83^

*Ranges are specified only for the parameters that vary in the uncertainty analysis; the specified ranges correspond to the 2.5 and 97.5 percentiles of the distributions from which the parameter values are sampled. ^†^The parameter is fixed for the baseline testing modalities, but a different parameter value is randomly sampled for each of the new testing modalities. **The parameter is estimated by dividing the reported fraction of partners who get tested (30%^60^) by the assumed probability of disclosure (0.71 in the case of married women). FP = family planning; FSW = female sex worker; MSM = men who have sex with men; OI = opportunistic infection; OR = odds ratio; RR = relative rate.

In addition to the existing HIV testing strategies, the model was extended to include a number of potential new HIV testing strategies:

- Home-based testing: This is assumed to be offered at an average frequency of once every two years. Male uptake is assumed to be lower than female uptake as men are less likely to be present when testing teams visit households^12^. Separate scenarios are run to compare the cost-effectiveness of home-based testing in urban and rural areas, and the effect of offering self-testing kits alongside it (as a strategy to increase uptake and get tests to household members who are not present at the time of the home visit).
- Mobile testing: Mobile clinics are assumed to move between communities, offering HIV testing. As with home-based testing, separate scenarios are run to compare the cost-effectiveness of mobile testing in urban and rural areas. A further scenario is run to evaluate the effect of using community mobilization to increase the uptake of mobile testing.
- Testing targeted to men who have sex with men (MSM): Men who have recently had sex with other men are assumed to be recruited into HIV testing programmes by peers^39,40^.
- Testing targeted to female sex workers (FSWs): Sex workers are encouraged to use dedicated sex worker services that offer HIV testing.
- Testing in family planning clinics: Women who use hormonal contraception (injectable methods or the pill) are assumed to be offered HIV testing on an annual basis.
- Assisted partner notification: Health workers are assumed to contact partners of newly-diagnosed individuals to offer them HIV testing, if the index patient consents and provides their partner’s contact details.
- Testing in schools: HIV testing in schools is assumed to be offered on an annual basis, with uptake being higher in sexually-experienced adolescents.
- Workplace testing: Uptake is conditional upon being in a workplace in which testing is offered. The fraction of the employed population working in the formal non-agricultural sector^41^ is considered an upper bound on the fraction of the employed population that could be offered testing annually.
- Testing partners of pregnant women: Pregnant women are issued invitation letters to give to their male partners, encouraging them to seek testing at the antenatal clinic. In an alternative scenario we consider the effect of instead providing pregnant women with self-testing kits to give to their male partners^17^.

Assumptions about the uptake and costs of these new testing modalities were based on a PubMed search (details in the supplementary material, section 1). Priority was given to African data sources and (where available) South African data in setting model assumptions. Where there was significant uncertainty around testing assumptions for the new testing modalities, prior distributions were assigned to represent the range of uncertainty around the relevant parameter (Table 1).

### Cost assumptions

We purpose-built a cost model for this analysis. All testing is assumed to follow a testing algorithm in keeping with the South African Department of Health’s testing cascade, which includes pre- and post-test counselling and rapid testing with confirmatory testing of positive results (Fig. S2.3). The test cascades in the cost model are divided into two groups, one for tests conducted in a healthcare facility, and another for those conducted through a mobile modality. We summarized cost and resource use for staff, consumables (including test kits) and equipment, overhead, and demand creation and targeting costs. Tests conducted in a facility were costed using a bottom-up approach for the staff, consumable and equipment costs and a top-down approach for the overhead costs, demand creation and targeting. Staff costs for facility-based testing were calculated based on observed time by client HIV status from a time-and-motion study. Tests conducted through a mobile modality were costed using a top-down approach for staff, equipment and overheads (because in a dedicated mobile testing service, these expenses are not split across other health activities), and consumable costs were calculated using a bottom-up approach. Resource use was calculated from the perspective of the provider, the public health system. All costs were updated to 2016–17 public-sector prices and salaries and converted to USD using the 07/2016 to 06/2017 period average of 1 USD = 13.58 ZAR^42^. Costs are presented unadjusted for inflation and undiscounted, in order to facilitate the use of total costs results for programme planning and budgeting. Further details regarding the cost assumptions are presented in section 2.6 of the supplementary material.

### Analysis

The model was used to project the expected number of new HIV infections and HIV-related deaths in South Africa over a 20-year period from 1 July 2019 to 30 June 2039. In the ‘baseline’ scenario we assumed no change to the existing HIV testing modalities, with rates of testing remaining constant at the levels estimated in 2016–17. Separate scenarios were defined for each of the potential new HIV testing modalities. For each of these new scenarios we calculated the change (relative to baseline) in total new HIV diagnoses, new HIV infections, life years lost due to HIV, HIV testing costs, and total HIV programme costs. Life years lost due to HIV were calculated using the West level 26 life table^43^. Two incremental cost-effectiveness ratio (ICER) measures were calculated over the 20-year period: the cost per HIV infection averted and the cost per life year saved, the latter being defined for consistency with the metric used in the South African HIV Investment Case^30^.

For the purpose of probabilistic sensitivity analysis, we randomly varied several of the model parameters simultaneously. As described elsewhere^32^, 100 different combinations of HIV transmission parameters and sexual behaviour parameters were identified that yielded model estimates of HIV prevalence consistent with historical South African HIV prevalence data. For each of these 100 parameter combinations we randomly sampled 5 parameter combinations from the distributions shown in Table 1, to generate a total of 500 different parameter combinations. For each of the scenarios described previously, the model was run for each of the 500 parameter combinations to generate a distribution of model outputs. From these we calculated means and standard errors, which were used to calculate 95% confidence intervals. Because ICER distributions were highly skewed, we summarized these instead using medians, with bootstrapping being used to estimate the 95% confidence intervals around the medians. To assess the sensitivity of the ICER estimates to the parameters that were allowed to vary across the 500 simulations, we fitted linear regression models to predict the change in ICER for unit changes in each of the parameters, and highlighted those parameters that had a statistically significant effect on the ICER. We also assessed the sensitivity of the model results to an arbitrary 50% reduction in the frequency of ‘general’ HIV testing over the 2019–39 period.

### Ethics

As this was a mathematical modelling study that did not involve human subjects, approval from an institutional review board was not necessary.

## Results

**Figure 2:**
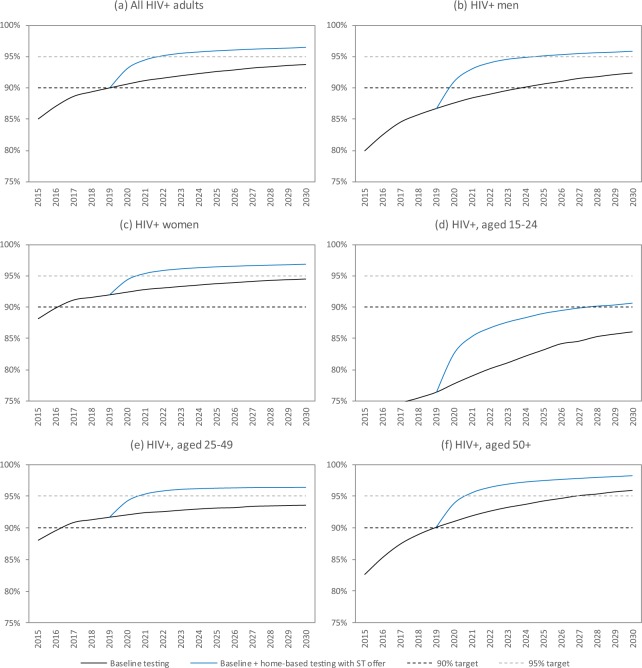
Projected progress towards the 90% and 95% targets for the fraction of HIV-positive adults who are diagnosed. ST = self-testing.

The model matches the reported total numbers of HIV tests performed and the reported levels of HIV prevalence in adults tested for HIV in the period up to 2016 (Fig. 1). By 2017, the model estimates that the fraction of HIV-positive adults who were diagnosed was 88.7%, though the proportion diagnosed was substantially lower among men (84.5%) than among women (91.2%), and substantially lower in the 15–24 age group (74.5%) than in the 25–49 and 50 + age groups (90.9% and 87.8% respectively) (Fig. 2). The fraction diagnosed was also relatively low among FSWs (82.9%) and MSM (81.4%) (Fig. S3.5). The model estimates that over the 2010–2015 period, the HIV testing modalities that accounted for the greatest numbers of HIV tests were general HIV testing (63.0%), testing in STI clinics (11.4%), testing of OI patients (9.8%) and antenatal testing (7.3%), while the testing modalities that accounted for the greatest numbers of new HIV diagnoses were general HIV testing (43.6%), testing of OI patients (32.5%), testing in STI clinics (8.1%), and testing partners of newly diagnosed individuals (7.4%) (Fig. S3.3).

The model estimates that in the absence of any change to current HIV testing strategies, 12–15 million adult HIV tests will be conducted each year over the next 20 years, and the fraction of tested individuals with positive results (including retests in previously-diagnosed individuals) will decline to around 5% (Fig. 1). Although South Africa is expected to meet the 90% diagnosis target by 2020, it is less likely to meet the 95% target by 2030, in the absence of any change to current HIV testing strategies (Fig. 2a). Reaching the 95% target is particularly unlikely in the case of men (Fig. 2b). Reaching the 90% and 95% targets is also expected to be challenging in groups that have high HIV incidence rates, notably youth (Fig. 2d), FSWs and MSM (Fig. S3.5). Of the baseline testing strategies, the strategies that are expected to lead to the highest rates of new diagnosis (per test performed) are testing partners of newly diagnosed individuals (6.8%) and testing of OI patients (6.8%). Yields are also expected to be relatively high in the case of males seeking MMC (2.3%), as most of these are young men who have not previously been tested. Yields on HIV testing in prisons and antenatal clinics are expected to be relatively low (0.7% and 1.1% respectively) because of the high rates of retesting in these settings.

**Figure 3:**
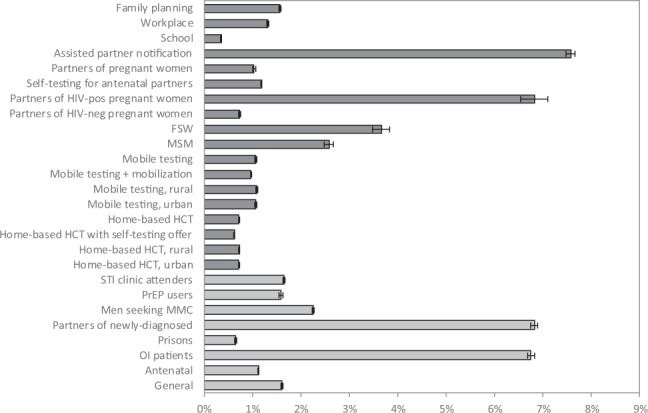
Fraction of tested individuals who are newly diagnosed, over the 2019–39 period. For the baseline testing modalities (in light grey), results are estimated in the baseline scenario. For the new testing modalities (in dark grey), results are estimated for the scenario in which the new testing modality is added to the baseline testing modalities. Error bars represent 95% confidence intervals.

The new HIV testing strategies that would most significantly increase the average annual number of HIV tests performed over the 2019–39 period include home-based testing coupled with an offer of self-testing (21.5 million additional HIV tests), standard home-based testing (16.0 million) and mobile testing with community mobilization (4.9 million) (Table S3.3). Although most strategies are expected to lead to increases in the total numbers of new diagnoses, some strategies (testing in MSM, FSWs, schools and partners of pregnant women) are expected to lead to reductions in the numbers of new diagnoses (Table S3.3), as the prevention benefits of testing imply a long-term reduction in new infections and thus a reduction in the maximum number of infections that can be diagnosed. The new strategies that are expected to achieve the highest rates of new diagnosis per test performed include assisted partner notification (7.6%), testing in FSWs (3.7%), and MSM (2.6%) (Fig. 3). Although testing partners of pregnant women is usually encouraged regardless of the woman’s HIV status, yields on such testing are expected to be substantially higher when the woman is HIV-positive (6.8%) than when the woman is HIV-negative (0.7%). Yields are expected to be lowest in the case of school-based testing (0.3%) and home-based testing coupled with self-testing (0.6%). Both for home-based and mobile testing, yields are expected to be similar in urban and rural areas.

In the absence of new testing strategies, the diagnosed fraction is expected to increase from 90.6% in 2020 to 93.7% by 2030 (Fig. 2a). The introduction of home-based testing with an offer of self-testing is expected to lead to the greatest increase in the fraction of HIV-positive adults diagnosed by 2030, 96.5% (Fig. S3.4). The introduction of this strategy would lead to the 95% target being met by 2030, both in men and women, although it would not be sufficient to achieve 95% diagnosis among youth (Fig. 2). Other strategies that would significantly increase the fraction diagnosed include regular home-based testing, mobile testing and testing in family planning clinics; most other new testing strategies, however, would have negligible impact on the overall fraction diagnosed (Fig. S3.4).

**Figure 4:**
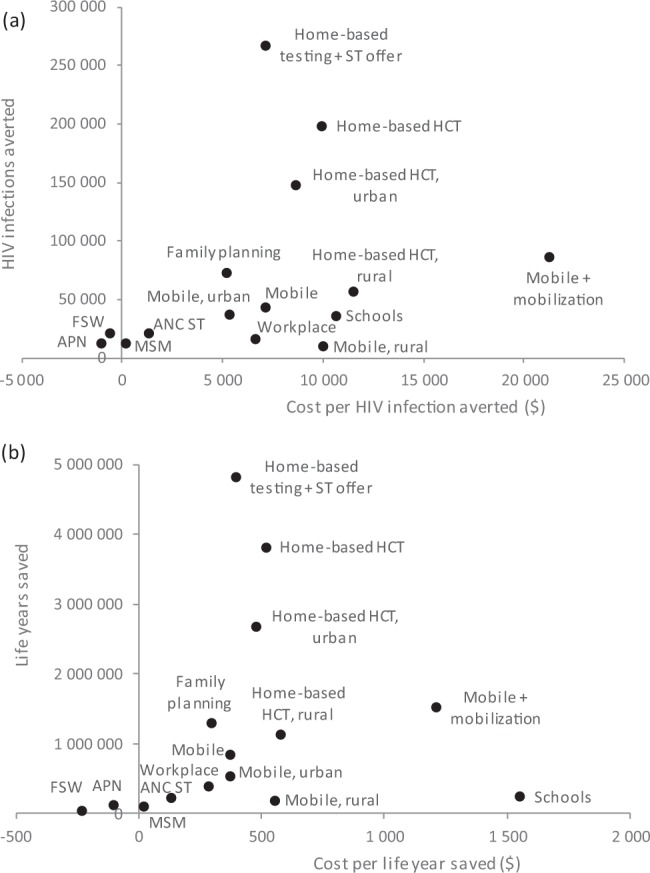
Impact and cost-effectiveness of potential new HIV testing strategies in South Africa, 2019–2039, in terms of HIV infections averted (**a**) and life years saved (**b**). The antenatal partner testing scenario was omitted as it did not lead to significant reductions in new infections or life year savings (Table S3.6). ANC ST = Antenatal clinic attender distribution of self-testing kits to partners; APN = assisted partner notification; FSW = female sex worker; HCT = HIV counselling and testing; MSM = men who have sex with men; ST = self-testing.

In the absence of any change to HIV testing policy, 5.5 million new adult HIV infections are expected in South Africa over the 2019–39 period, and 68 million life years are expected to be lost due to HIV-related deaths in this period. As a result of the modelled increases in condom use and ART initiation following diagnosis, all of the potential new HIV testing strategies are expected to lead to reductions in new HIV infections (Fig. 4a and Table S3.6). More substantial reductions are expected in the total numbers of life years lost due to HIV (Fig. 4b and Table S3.6). The greatest reductions are expected in the case of home-based testing coupled with an offer of self-testing: a median of 268 000 infections averted (95% CI: 249 000–288 000) and a median of 4.8 million life years saved (95% CI: 4.7–5.0 million).

The average cost per test is expected to be lowest in the case of self-testing, when offered through home-based testing ($3.08 per test) and when distributed to partners of pregnant women ($3.14), due to the saving in health worker time (Table S3.2). The cost per test is expected to be highest in the case of mobile testing with community mobilization ($17.46), due to the high costs of the community mobilization events, which we assumed to include some catering. Other relatively expensive strategies include testing in schools ($7.08) and assisted partner notification ($6.95). Average test costs for home-based and mobile testing are roughly 30% lower in urban areas than in rural areas, due to the greater distances that need to be travelled in the latter.

Total HIV testing costs, in the absence of any change to current testing strategy, are expected to be an average of $64 million over the 2019–2039 period (Table S3.4). This is equivalent to 3.4% of the projected annual cost of all HIV programmes combined ($37.7 billion over the 2019–2039 period, or $1.88 billion per year (Table S3.5)). The greatest increases in total testing costs would be expected in the mobile testing scenario with community mobilization (a median increase of $85 million (95% CI: $82–87 million) in the average annual testing cost), the regular home-based testing scenario (median increase $74 million, 95% CI: $73–74 million) and the home-based testing with an offer of self-testing scenario (median increase $66 million, 95% CI: $66–67 million). When taking into account the total HIV programme costs, two of the potential new strategies are expected to be cost-saving (assisted partner notification and testing in FSWs), although the change in net cost is not significantly different from zero (Table S3.5). As a result, these two testing strategies had negative median ICERs, both for the cost per HIV infection averted and the cost per life year saved (Fig. 4), though the upper 95% confidence limits extended above zero (Table S3.8). Other strategies with low ICERs included testing in MSM (median of $20 per life year saved, 95% CI: cost saving-$320), secondary distribution of self-tests to partners of pregnant women ($130, 95% CI: $34–263), workplace testing ($283, 95% CI: $203–378), testing in family planning clinics ($294, 95% CI: $251–350), mobile testing in urban areas ($370, 95% CI: $261–537) and home-based testing with the offer of self-testing ($394, 95% CI: 379–410). The highest cost per life year saved was $1 549 (95% CI: $1 037–2 686) in the case of school-based testing and $1 209 (95% CI: 1 105–1 340) in the case of mobile testing with community mobilization.

In the regression analysis, ICER estimates were relatively insensitive to changes in the parameters specific to the new testing modalities (Table S3.13). However, in several scenarios the ICER reduced significantly as the relative rate of testing in previously-diagnosed individuals increased (Table S3.11), because previously-diagnosed individuals were assumed to be more likely to start ART after a retest, and similarly ICERs tended to decrease as rates of ART initiation following community-based testing increased. In contrast, the ICERs tended to increase as the relative rate of testing in treated adults increased, as there was no assumed benefit to testing individuals who were already on ART. The ICERs were also significantly negatively related to the projected future HIV incidence trend (in the absence of changes in testing policy). For example, in the home-based testing with self-testing scenario, the cost per life year saved reduced by $220 for every 1 million increase in the projected number of new infections over the 2019–39 period (Table S3.15). ICERs were also sensitive to the level of HIV testing in the baseline scenario: if it was assumed that there was a 50% reduction in the rate of general HIV testing over the 2019–2039 period, the cost per life year saved in the home-based testing with self-testing scenario decreased from $394 to $316 (Table S3.16).

## Discussion

As noted recently by De Cock and colleagues, there is a clear tension between pursuing the HIV testing strategies that are most efficient and the strategies that have the greatest impact^10^. In our analysis, strategies such as assisted partner notification, testing in FSWs and MSM, and secondary distribution of self-testing to partners of pregnant women were highly cost-effective (possibly even cost-saving) but had only a very modest impact on population-level HIV incidence and mortality. In contrast, home-based HIV testing was predicted to have the most substantial impact on HIV incidence and mortality, but at the expense of relatively low yields and cost-effectiveness. Despite this, the cost per life year saved of home-based testing coupled with self-testing was $394, well below the willingness-to-pay threshold of $547–842 previously estimated for South Africa^30^. Indeed, most of the HIV testing strategies had ICERs below this threshold, with the exception of school-based testing and mobile testing combined with community mobilization. These findings are important, as it is often assumed that home-based testing is unlikely to be efficient in settings such as South Africa, where a high fraction of HIV-positive individuals already know their HIV status^10^. Although South Africa is already close to the 90% diagnosis target in adults, South Africa is unlikely to achieve the 95% target by 2030 in the absence of major changes to HIV testing programmes, and strategies such as home-based testing may be crucial to achieving this.

The challenges that South Africa faces in reaching the 95% target are to some extent a reflection of the challenges that the country faces in reducing HIV incidence, as the fraction diagnosed can only be increased substantially if the annual number of new diagnoses exceeds the annual number of new infections. This explains why the fraction diagnosed is lowest in the sub-populations in which HIV incidence is highest (FSWs, youth and MSM), and our results suggest that even with very aggressive HIV testing strategies, the 95% target is unlikely to be met in these high-incidence groups (Fig. 2 and Fig. S3.5). The MicroCOSM model forecasts a higher average number of new adult HIV infections per annum over the 2019–39 period (275 000) than the Thembisa model (170 000), the model on which UNAIDS estimates for South Africa are based^29^. MicroCOSM thus forecasts a lower fraction of HIV-positive adults diagnosed by 2030 than Thembisa (93.8% versus 95.7%). If the Thembisa forecast of 170 000 new infections per annum were more accurate than the MicroCOSM forecast, corresponding increases in the costs per life year saved might be expected. For example, in the home-based testing with self-testing scenario, the cost per life year saved would increase from $394 to $856 (394–0.00022 × (170 000–275 000) × 20, where the −0.00022 is from the regression model in Table S3.15), making it a less favourable policy option when compared against the current willingness-to-pay threshold. Projections of progress towards the 95% targets and cost-effectiveness are thus very sensitive to future HIV incidence trends, which are difficult to predict with confidence.

This analysis suggests that assessing HIV testing based on numbers of new diagnoses may be problematic, for two reasons. Firstly, our analysis shows that some HIV testing strategies might lead to increases in new diagnoses in the short term but longer-term reductions in new diagnoses as a result of reductions in HIV incidence. Secondly, much of the modelled benefit of testing arises from retesting previously-diagnosed individuals who have either never linked to HIV care or dropped out of care. Although such retesting of previously-diagnosed individuals is often considered a ‘waste’ of scare testing resources, it may be important in facilitating linkage to care and (re)initiation of ART. A recent South African study found similar rates of linkage to HIV care in newly-diagnosed adults and previously-diagnosed adults who had not previously linked to care, and higher rates of linkage in individuals who had dropped out of care^38^. Our sensitivity analyses suggest that the cost per life year saved reduces significantly as the relative rate of testing in previously-diagnosed untreated individuals increases, and thus it is important to consider repeat diagnoses as well as new diagnoses.

Our results point to the significant potential benefits of self-testing in improving both the impact and the cost-effectiveness of home-based testing and testing partners of pregnant women. This is in part because of the significantly higher HIV testing uptake associated with self-testing^16^, and in part because of the lower cost per HIV-negative test result when there is less health worker time required^22,23^. However, the lower cost assumption may be simplistic, as it does not take into consideration the cost of distributing the self-test, due to lack of data. Another potential concern is that self-testing may be less sensitive than standard testing performed by health workers, although a recent review found good agreement between self-testing and health worker-administered testing in most studies^44^. We have limited our analysis to two scenarios in which testing uptake would be low in the absence of self-testing: many individuals are not at home when home-based testing teams visit, and getting partners of pregnant women to come to antenatal clinics for testing is frequently challenging, making the secondary distribution of self-testing kits an obvious strategy in such scenarios. However, in settings in which the uptake of HIV testing by health workers is already high (for example, HIV testing in antenatal clinics and TB patients), the increase in HIV testing uptake associated with an offer of self-testing is likely to be minimal. Further work is required to improve our estimates of the cost of distributing HIV self-test kits, and to model the potential cost-effectiveness of offering self-testing to FSWs, a group that has been shown to benefit significantly from this intervention^18,19^. The offer of self-testing in outpatient departments could also significantly increase HIV testing rates^45^, and should also be evaluated in future modelling. It may also be useful to consider a more targeted approach to the secondary distribution of self-testing kits, such as limited to pregnant women who do not know their partner’s HIV status – although our current model is not capable of modelling such a scenario, and such targeting is not being considered in South Africa.

Our results show that in the context of mobile testing, including community mobilization activities substantially increases the epidemiological impact, but also substantially increases the cost per life year saved (above the willingness to pay threshold). A limitation of our analysis is that we do not consider the cost and impact of community mobilization and sensitization in the context of other new models of community-based HIV testing (particularly home-based testing), due to lack of local data. To some extent community care workers (who we have assumed to conduct home-based testing) do already perform community sensitization, but it will be important for future analyses to consider whether additional community mobilization activities are economically justified.

A key strength of this analysis is that it is based on a network model, which means that individuals are dynamically linked to specific partners, making it possible to model realistically the effects of disclosure of HIV status to partners and the benefits of partner notification strategies. Our analysis suggests that assisted partner notification and testing partners of pregnant women are likely to be among the most cost-effective strategies, with high yields. However, the relatively small population-level impact of these testing strategies is in part because disclosure occurs less frequently in short-term relationships than in long-term marital relationships, and rates of marriage are relatively low in South Africa^46^. Partner notification strategies may have relatively more impact in other African settings, in which a higher fraction of HIV-positive adults are married or cohabiting.

A disadvantage of the network modelling approach is that it is individual-based, which introduces substantial stochastic variation in model outputs. As a result, the 95% confidence intervals around the model estimates tend to be wide, and in some cases this makes the ranking of different HIV testing strategies difficult, especially when comparing ICERs, which have the widest confidence intervals.

Another limitation of this analysis is that it does not consider HIV testing strategies for children^47^. We have also not included infections averted in children and life years saved in children (as a result of reduced mother-to-child transmission) when estimating the ICERs. Our analysis also does not consider couple-based testing^48^, although – as noted previously – rates of cohabitation and marriage in South Africa are lower than in most other African settings, and this may present a challenge for recruiting couples in our setting. We also did not vary our input costs in the uncertainty analysis, and the costs of the novel testing modalities that have not been implemented at scale in the public sector (in particular self-test distribution) are currently based on assumptions regarding staff time, linkage and the number of tests that can be performed per day. Rates of linkage to ART after diagnosis are difficult to determine in the context of community-based models of HIV testing, which generally suffer from lower rates of linkage than facility-based testing^18,49–51^. In our uncertainty analyses, we found that in most community-based testing scenarios the cost per life year saved declined substantially as the rate of linkage to ART increased (Table S3.11), and it is therefore important to quantify linkage rates more precisely. ICERs may be similarly affected by future changes in ART effectiveness and cost, but we have conservatively assumed no future changes in costs or effectiveness in our analyses.

Some of our results may not be generalizable to other African settings. The cost-effectiveness of HIV testing is sensitive to the levels of HIV prevalence in the population^52^, which are relatively high in South Africa, and costs of HIV interventions tend to be higher in South Africa than in other African settings^53^. However, the *relative* impact and cost-effectiveness of different testing strategies is likely to be more similar across settings. Possible exceptions are testing strategies that target key populations, which are likely to be relatively more cost-effective (compared to testing in the general population) in the more concentrated epidemic settings of West and Central Africa, and partner notification, which may have relatively more impact in settings that have higher rates of marriage. In this analysis we have compared HIV testing strategies in terms of the cost per life year saved, rather than the cost per disability-adjusted life year (DALY) averted, which would allow for comparison with other disease areas and be more in line with economic analyses that aim to inform decisions of international funders. The primary motivation for using this metric is to achieve consistency with the metric used in the South African HIV Investment Case, which estimated the government’s ‘willingness to pay’ threshold for HIV interventions^30^, but other metrics may be more appropriate in other African settings. Our model can be applied to other African countries, although considerable work would be required to parameterize and calibrate the model for other settings, given the large number of socio-demographic and epidemiological assumptions.

As many African countries move closer to achieving the 90% diagnosis target, there has been growing concern over whether it is feasible to sustain the same intensity of testing efforts as has been achieved in the past, and whether it may be appropriate to scale back to a more ‘targeted’ approach to HIV testing strategies that are associated with higher yields^10,11^. Our results suggest that focusing only on yield may be simplistic, and that even HIV testing strategies with relatively low yields may be cost-effective when compared to other currently-funded HIV interventions. Although it may be efficient to reduce the frequency of screening in certain populations (for examples, pregnant women and prisoners), more rather than less testing may ultimately be appropriate if substantial reductions in HIV incidence and mortality are to be achieved.

## Supplementary information

Supplementary materials

## Data Availability

This analysis is based on simulated data generated by a mathematical model. The mathematical model and summaries of the data are available from the corresponding author on request.

## References

[1] UNAIDS. Miles to go - closing gaps, breaking barriers, righting injustices. Global AIDS update 2018. http://www.unaids.org/sites/default/files/media_asset/miles-to-go_en.pdf?utm_source=UNAIDS+Newsletter&utm_campaign=fc6ccfe4b0-EMAIL_CAMPAIGN_2018_07_18_07_53_COPY_01&utm_medium=email&utm_term=0_e7a6256e25-fc6ccfe4b0-114148825. Accessed 20 July 2018 (2018).

[2] UNAIDS. Fast-track - Ending the AIDS epidemic by 2030. http://www.unaids.org/en/resources/documents/2014/JC2686_WAD2014report. Accessed 17 Oct 2015 (2014).

[3] Maman D (2016). Closer to 90-90-90. The cascade of care after 10 years of ART scale-up in rural Malawi: a population study. J Int AIDS Soc.

[4] Haber N (2017). From HIV infection to therapeutic response: a population-based longitudinal HIV cascade-of-care study in KwaZulu-Natal, South Africa. Lancet HIV.

[5] Gaolathe T (2016). Botswana’s progress toward achieving the 2020 UNAIDS 90-90-90 antiretroviral therapy and virological suppression goals: a population-based survey. Lancet HIV.

[6] Huerga H (2016). Who needs to be targeted for HIV testing and treatment in KwaZulu-Natal? Results from a population-based survey. J Acquir Immun Defic Syndr.

[7] Grobler A, Cawood C, Khanyile D, Puren A, Kharsany ABM (2017). Progress of UNAIDS 90-90-90 targets in a district in KwaZulu-Natal, South Africa, with high HIV burden, in the HIPSS study: a household-based complex multilevel community survey. Lancet HIV.

[8] Lane T (2014). The Mpumalanga Men’s Study (MPMS): Results of a baseline biological and behavioral HIV surveillance survey in two MSM communities in South Africa. PLoS One.

[9] Risher K, Mayer KH, Beyrer C (2015). HIV treatment cascade in MSM, people who inject drugs, and sex workers. Curr Opin HIV AIDS.

[10] De Cock KM, Barker JL, Baggaley R, El Sadr WM (2019). Where are the positives? HIV testing in sub-Saharan Africa in the era of test and treat. AIDS.

[11] U.S. President’s Emergency Plan for AIDS Relief. PEPFAR 2019 Country Operational Plan Guidance for all PEPFAR Countries. https://www.pepfar.gov/documents/organization/288160.pdf. Accessed 20 Feb 2019 (2019).

[12] Sharma M, Ying R, Tarr G, Barnabas R (2015). Systematic review and meta-analysis of community and facility-based HIV testing to address linkage to care gaps in sub-Saharan Africa. Nature.

[13] Hensen B, Taoka S, Lewis JJ, Weiss HA, Hargreaves J (2014). Systematic review of strategies to increase men’s HIV-testing in sub-Saharan Africa. AIDS.

[14] Suthar AB (2013). Towards universal voluntary HIV testing and counselling: a systematic review and meta-analysis of community-based approaches. PLoS Med.

[15] Dalal S (2017). Improving HIV test uptake and case finding with assisted partner notification services. AIDS.

[16] Johnson CC (2017). Examining the effects of HIV self-testing compared to standard HIV testing services: a systematic review and meta-analysis. J Int AIDS Soc.

[17] Masters SH (2016). Promoting partner testing and couples testing through secondary distribution of HIV self-tests: A randomized clinical trial. PLoS Med.

[18] Chanda MM (2017). HIV self-testing among female sex workers in Zambia: A cluster randomized controlled trial. PLoS Med.

[19] Ortblad K (2017). Direct provision versus facility collection of HIV self-tests among female sex workers in Uganda: A cluster-randomized controlled health systems trial. PLoS Med.

[20] Ayles, H. *et al*. Increasing knowledge of HIV status among men: a cluster-randomised trial of community-based distribution of oral HIV self-test kits nested in four HPTN 071 communities in Zambia [Abstract TUAC0406LB]. In *9th International AIDS Society Conference*. (2017).

[21] Pettifor, A. *et al*. HIV self-testing increases testing in young South African women: results of an RCT [Abstract 992]. In *25th Conference on Retroviruses and Opportunistic Infections*. (2018).

[22] Korenromp, E. & Stover, J. HIV testing: program pathways for scale-up to the 90% knowledge target - epidemiological projections and country typologies. (2015).

[23] Cambiano V (2015). Assessment of the potential impact and cost-effectiveness of self-testing for HIV in low-income countries. J Infect Dis.

[24] Bassett IV (2014). Mobile HIV screening in Cape Town, South Africa: clinical impact, cost and cost-effectiveness. PLoS One.

[25] Olney JJ (2016). Evaluating strategies to improve HIV care outcomes in Kenya: a modelling study. Lancet HIV.

[26] Smith JA (2015). Cost-effectiveness of community-based strategies to strengthen the continuum of HIV care in rural South Africa: a health economic modelling analysis. Lancet HIV.

[27] Ying R (2016). Home testing and counselling to reduce HIV incidence in a generalised epidemic setting: a mathematical modelling analysis. Lancet HIV.

[28] Sharma M (2018). Assisted partner notification services are cost-effective for decreasing HIV burden in western Kenya. AIDS.

[29] Johnson LF, Dorrington RE, Moolla H (2017). Progress towards the 2020 targets for HIV diagnosis and antiretroviral treatment in South Africa. South Afr J HIV Med.

[30] Meyer-Rath G, van Rensburg C, Larson B, Jamieson L, Rosen S (2017). Revealed willingness-to-pay versus standard cost-effectiveness thresholds: Evidence from the South African HIV Investment Case. PLoS One.

[31] Johnson LF, Geffen N (2016). A comparison of two mathematical modeling frameworks for evaluating sexually transmitted infection epidemiology. Sex Transm Dis.

[32] Johnson, L. F., Kubjane, M. & Moolla, H. MicroCOSM: a model of social and structural drivers of HIV and interventions to reduce HIV incidence in high-risk populations in South Africa. *BioRxiv* (2018).

[33] Shisana, O. *et al*. South African National HIV Prevalence, HIV Incidence, Behaviours and Communication Survey, 2005., http://www.hsrcpress.ac.za. Accessed 1 Dec 2005 (HSRC Press, Cape Town, 2005).

[34] Shisana, O. *et al*. South African national HIV prevalence, incidence, behaviour and communication survey, 2008: A turning tide among teenagers?, (Human Sciences Research Council http://www.hsrcpress.ac.za. Accessed 9 June 2009, Cape Town, 2009).

[35] Shisana, O. *et al*. South African National HIV Prevalence, Incidence, and Behaviour Survey, 2012. http://www.hsrc.ac.za/en/research-outputs/view/6871. Accessed 16 April 2014 (Human Sciences Research Council, Cape Town, 2014).

[36] Department of Health. The 2012 National Antenatal Sentinel HIV and Herpes Simplex Type-2 Prevalence Survey in South Africa. http://www.health.gov.za/reports.php. Accessed 14 May 2014 (2014).

[37] Johnson, L. F. & Dorrington, R. E. Thembisa version 4.1: A model for evaluating the impact of HIV/AIDS in South Africa. (2018).

[38] Plazy M (2016). Access to HIV care in the context of universal test and treat: challenges within the ANRS 12249 TasP cluster-randomized trial in rural South Africa. J Int AIDS Soc.

[39] Lane T (2016). High HIV incidence in a South African community of men who have sex with men: results from the Mpumalanga Men’s Study, 2012-2015. J Acquir Immun Defic Syndr.

[40] Geibel S, King’ola N, Temmerman M, Luchters S (2012). The impact of peer outreach on HIV knowledge and prevention behaviours of male sex workers in Mombasa, Kenya. Sex Transm Infect.

[41] Statistics South Africa. Quarterly Labour Force Survey, Quarter 1: 2018. http://www.statssa.gov.za/publications/P0211/P02111stQuarter2018.pdf. Accessed 20 Dec 2018 (Pretoria, 2018).

[42] South African Reserve Bank. Mid-year average USD-ZAR conversion rates. https://www.resbank.co.za/publications/detail-item-view/pages/publications.aspx?sarbweb=3b6aa07d-92ab-441f-b7bf-bb7dfb1bedb4&sarblist=21b5222e-7125-4e55-bb65-56fd3333371e&sarbitem=7921. Accessed 20 Aug 2017 (2017).

[43] Murray CJ (1994). Quantifying the burden of disease: the technical basis for disability-adjusted life years. Bull WHO.

[44] Figueroa C (2018). Reliability of HIV rapid diagnostic tests for self-testing compared with testing by health-care workers: a systematic review and meta-analysis. Lancet HIV.

[45] Dovel, K. *et al*. Facility-based HIV self-testing for outpatients dramatically increases HIV testing in Malawi: A cluster randomized trial [Abstract TUAE0105]. In *22nd International AIDS Conference*. (2018).

[46] Bongaarts J (2007). Late marriage and the HIV epidemic in sub-Saharan Africa. *Pop*. Studies.

[47] Kellerman S, Essajee S (2010). HIV testing for children in resource-limited settings: what are we waiting for?. PLoS Med.

[48] Kilembe W (2015). Implementation of couples’ voluntary HIV counseling and testing services in Durban, South Africa. BMC Public Health.

[49] Bogart LM (2017). A comparison of home-based versus outreach event-based community HIV testing in Ugandan fisherfolk communities. AIDS Behav.

[50] Schwartz S (2017). Engagement in the HIV care cascade and barriers to antiretroviral therapy uptake among female sex workers in Port Elizabeth, South Africa: findings from a respondent-driven sampling study. Sex Transm Infect.

[51] MacKellar DA (2016). Enrollment in HIV care two years after HIV diagnosis in the kingdom of Swaziland: An evaluation of a national program of new linkage procedures. PLoS One.

[52] Cambiano V (2019). The impact and cost-effectiveness of community-based HIV self-testing in sub-Saharan. Africa: a health economic and modelling analysis. J Int AIDS Soc.

[53] Tagar E (2014). Multi-country analysis of treatment costs for HIV/AIDS (MATCH): facility-level ART unit cost analysis in Ethiopia, Malawi, Rwanda, South Africa and Zambia. PLoS One.

[54] Fuente-Soro L (2018). Monitoring progress towards the first UNAIDS target: understanding the impact of people living with HIV who re-test during HIV-testing campaigns in rural Mozambique. J Int AIDS Soc.

[55] Peltzer K, Chao LW, Dana P (2009). Family planning among HIV positive and negative prevention of mother to child transmission (PMTCT) clients in a resource poor setting in South Africa. AIDS Behav.

[56] Hilderbrand K, Goemaere E, Coetzee D (2003). The prevention of mother-to-child HIV transmission programme and infant feeding practices. S Afr Med J.

[57] Makin JD (2008). Factors affecting disclosure in South African HIV-positive pregnant women. AIDS Patient Care STDs.

[58] Haberlen SA (2015). Antiretroviral therapy availability and HIV disclosure to spouse in Rakai, Uganda: a longitudinal population-based study. J Acquir Immun Defic Syndr.

[59] Ostermann J (2015). HIV serostatus disclosure in the treatment cascade: evidence from Northern Tanzania. AIDS Care.

[60] Aluisio A (2011). Male antenatal attendance and HIV testing are associated with decreased infant HIV infection and increased HIV-free survival. J Acquir Immun Defic Syndr.

[61] Brown LB (2011). HIV partner notification is effective and feasible in sub-Saharan Africa: opportunities for HIV treatment and prevention. J Acquir Immun Defic Syndr.

[62] Rosenberg NE (2015). Recruiting male partners for couple HIV testing and counselling in Malawi’s option B+ programme: an unblinded randomised controlled trial. Lancet HIV.

[63] Church K (2017). Impact of integrated services on HIV testing: A nonrandomized trial among Kenyan family planning clients. Stud Fam Plann.

[64] Liambila W (2009). Feasibility and effectiveness of integrating provider-initiated testing and counselling within family planning services in Kenya. AIDS.

[65] Kranzer K (2011). High prevalence of self-reported undiagnosed HIV despite high coverage of HIV testing: a cross-sectional population based sero-survey in South Africa. PLoS One.

[66] Bassett IV (2015). Finding HIV in hard to reach populations: mobile HIV testing and geospatial mapping in Umlazi township, Durban, South Africa. AIDS Behav.

[67] Sweat M (2011). Community-based intervention to increase HIV testing and case detection in people aged 16-32 years in Tanzania, Zimbabwe, and Thailand (NIMH Project Accept, HPTN 043): a randomised study. Lancet Infect Dis.

[68] Ghys PD (2002). Increase in condom use and decline in HIV and sexually transmitted diseases among female sex workers in Abidjan, Côte d’Ivoire, 1991-1998. AIDS.

[69] Cowan FM (2018). Targeted combination prevention to support female sex workers in Zimbabwe accessing and adhering to antiretrovirals for treatment and prevention of HIV (SAPPH-IRe): a cluster-randomised trial. Lancet HIV.

[70] Lafort Y (2018). Effect of a ‘diagonal’ intervention on uptake of HIV and reproductive health services by female sex workers in three sub-Saharan African cities. Trop Med Int Health.

[71] Madiba S, Mokgatle M (2015). “Students want HIV testing in schools” a formative evaluation of the acceptability of HIV testing and counselling at schools in Gauteng and North West provinces in South Africa. BMC Public Health.

[72] Meinck, F., Carty, C. & Cluver, L. In *INTEREST Workshop* (Yaoundé, Cameroon, 2016).

[73] Van der Borght SF (2010). Long-term voluntary counseling and testing (VCT) uptake dynamics in a multicountry HIV workplace program in sub-Saharan Africa. AIDS Care.

[74] Krakowiak D (2016). Home-based HIV testing among pregnant couples increases partner testing and identification of serodiscordant partnerships. Journal of Acquired Immune Deficiency Syndrome.

[75] Osoti AO (2014). Home visits during pregnancy enhance male partner HIV counselling and testing in Kenya: a randomized clinical trial. AIDS.

[76] Jefferys LF, Nchimbi P, Mbezi P, Sewangi J, Theuring S (2015). Official invitation letters to promote male partner attendance and couple voluntary HIV counselling and testing in antenatal care: an implementation study in Mbeya Region, Tanzania. Reproductive Health.

[77] Byamugisha R (2011). Male partner antenatal attendance and HIV testing in eastern Uganda: a randomized facility-based intervention trial. J Int AIDS Soc.

[78] Mohlala BK, Boily MC, Gregson S (2011). The forgotten half of the equation: randomized controlled trial of a male invitation to attend couple voluntary counselling and testing. AIDS.

[79] Msuya SE (2008). Low male partner participation in antenatal HIV counselling and testing in northern Tanzania: implications for preventive programs. AIDS Care.

[80] Katz DA (2009). Male perspectives on incorporating men into antenatal HIV counseling and testing. PLoS One.

[81] Massyn, N. *et al*. District Health Barometer 2015/16. http://www.hst.org.za/publications/district-health-barometer-201516-0. Accessed 5 March 2017 (Durban, 2016).

[82] Rosen S, Fox MP (2011). Retention in HIV care between testing and treatment in sub-Saharan Africa: a systematic review. PLoS Med.

[83] Department of Health & South African National AIDS Council. South African HIV and TB Investment Case - Summary Report Phase 1. http://sanac.org.za/wp-content/uploads/2016/03/1603-Investment-Case-Report-LowRes-18-Mar.pdf. Accessed 31 May 2016 (2016).

