# Supplementary material for: Optimal HIV testing strategies for South Africa: a model-based evaluation of population-level impact and cost-effectiveness
Source: Sci Rep. 2019 Sep 2;9:12621. doi: 10.1038/s41598-019-49109-w (PMC6718403; doi:10.1038/s41598-019-49109-w)
Supplement: Supplementary file 1 — Supplementary materials [file 41598_2019_49109_MOESM1_ESM.pdf]

Optimal HIV testing strategies for South Africa: a model-based evaluation of population-level impact and cost-effectiveness

Supplementary materials

Leigh F. Johnson[1]
Craig van Rensburg[2,3]
Caroline Govathson[2,3]
Gesine Meyer-Rath[2,3,4]

1. Centre for Infectious Disease Epidemiology and Research, University of Cape Town, South Africa
2. Health Economics and Epidemiology Research Office, University of the Witwatersrand, Johannesburg, South Africa
3. Department of Medicine, Faculty of Health Sciences, University of the Witwatersrand, Johannesburg, South Africa
4. Department of Global Health, Boston University School of Public Health, Boston, USA

Table of contents

1. Literature review 4
   1.1 Search strategy 4
   1.2 Results and uses 4
      1.2.1 Papers on testing uptake 4
      1.2.1 Papers on the cost of testing 6
2. Methods 8
   2.1 Methods overview 8
   2.2 Current South African HIV testing modalities 12
      2.2.1 General HIV testing 12
      2.2.2 Testing of patients with HIV-related opportunistic infections 14
      2.2.3 Testing of pregnant women 15
      2.2.4 Testing of partners of diagnosed individuals 16
      2.2.5 Testing of STI patients 17
      2.2.6 Testing of men who seek medical male circumcision (MMC) 18
      2.2.7 Testing of men in prison 19
      2.2.8 Testing of sex workers and MSM receiving PrEP 19
   2.3 New HIV testing strategies 20
      2.3.1 Assisted partner notification 20
      2.3.2 Home-based testing 21
      2.3.3 Testing of women attending family planning clinics 23
      2.3.4 Mobile testing 24
      2.3.5 HIV testing targeted to MSM 25
      2.3.6 HIV testing targeted to sex workers 26
      2.3.7 HIV testing targeted to adolescents/school-based testing 27
      2.3.8 HIV testing in workplaces 28
      2.3.9 Testing of male partners of pregnant women 30
      2.3.10 Self-testing 31
   2.4 Test sensitivity and specificity 33
   2.5 Linkage to care after diagnosis 34
   2.6 Cost model 36
      2.6.1 Structure 36
      2.6.2 Calculations: Average and total testing cost and total programme cost 38

2.6.3 Calculations: Cost effectiveness 38
2.6.4 Cost inputs 38
3. Additional results 44
3.1 HIV testing and diagnosis up to 2016 44
3.2 Future trends in HIV test uptake and diagnosis 46
3.3 Average and total cost, number of tests and additional diagnoses 49
3.4 Effectiveness 54
3.5 Cost effectiveness 55
3.6 Sensitivity analysis 57
4. Comparison with other modelling studies 67
References 70
Annex 1: Average cost and cost per category by testing modality 83

1. Literature review

We conducted a number of literature reviews to identify previous research on community-based testing, following three different strategies: Firstly, we reviewed existing systematic reviews of literature of HIV counselling and testing (HCT). Secondly, we identified appropriate papers from records identified during an existing review of literature reporting on testing covering men. Thirdly, we additionally searched PubMed for papers on the testing modalities included in the analysis.

1.1 Search strategy

For the literature search concerning testing of men, we searched PubMed using search terms denoting HIV, testing, and men, male, father or partner. For the second search, we searched PubMed using combinations of search terms denoting HIV, testing, and the testing modalities included in the analysis (i.e. home-based, mobile, campaign, school or index testing, partner notification, self-testing or medical male circumcision).

The searches were conducted between September 2016 and April 2017 and included papers written in any language between 2007 (the year of the first WHO guidance explicitly mentioning rapid tests) [1] and 2017.

1.2 Results and uses

1.2.1 Papers on testing uptake

Our review included a total of 3,179 records, of which 300 (9%) were duplicates. 454 of the 3,179 records (including 210 duplicates) came from published literature reviews of HCT; 813 (including 80 duplicates) from the review of HCT options for men, and 1,901 (including 10 duplicates) from our additional review of community-based testing. Figure S1.1 summarises the findings of the review; Table S1.1 gives the results by type of search/ review.

68% of the screened records were excluded at the abstract/title level; 77% of all papers assessed at the full text level were excluded, 57% of which were because they reported on testing interventions outside of sub-Saharan Africa (SSA), and 33% because they did not report on testing uptake. Overall, 23% of all assessed records, or 10% of all identified records were included, with the yield being slightly higher (8%) for the papers identified through the review of published studies and the review of HCT options for men than for the additional search covering community-based modalities (6%).

Of the 205 studies identified through the literature review, 40 were used to inform model parameters on testing uptake by age, gender, risk group, HIV testing history and HIV status.

Table S1.1: Results of literature review by review method

	Records identified	Duplicates	Records screened	Records excluded	Full-text articles assessed	Full-text articles excluded					Studies included	Yield	Cost data
						Paper not found	No data on testing uptake	Non SSA	Other	Total excluded			
1. Review of published literature reviews	465	210	254	102	153	7	90	0	0	116	37	8%	4
2. Search as part of Testing men review	813	80	733	228	503	20	85	322	10	437	66	8%	0
3. Additional search for community-based testing	1,901	10	1,891	1,637	254	1	58	83	10	152	102	6%	14
All	3,179	300	2,878	1,967	910	28	233	405	20	705	205	6%	18

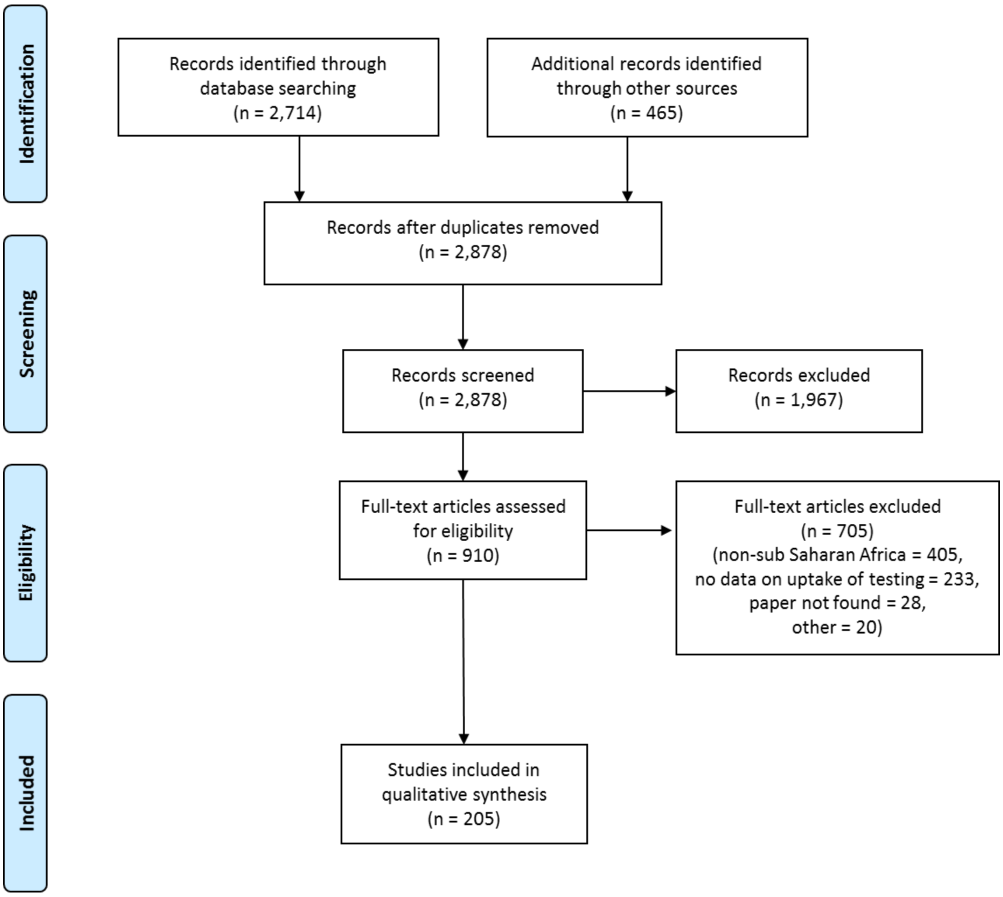

Figure S1.1: Diagram of literature review for literature on the uptake and costs of community-based testing modalities

1.2.1 Papers on the cost of testing

Costs were mentioned in only 18 of the identified papers, with 15 papers reported for sub-Saharan Africa (4 papers for Uganda and Malawi each, 3 for South Africa, 2 for Kenya, 1 for Lesotho and Swaziland each). Most of the SSA papers reported on home-based testing (9 papers), with 3 papers each reporting on self-testing and demand-creation campaigns. A further 3 reported on mobile testing, and 1 on testing as part of MMC (papers could report on more than one modality). All papers except one reported on costs from the provider perspective; the one exception reported on the patient cost of facility-based testing (mostly travel costs).

None of the studies containing cost data were used for the economic evaluation, as we required South Africa-specific data for this, and the three South African cost analyses either did not include the cost of HCT [2, 3] or reported on patient instead of healthcare provider costs [4]. We instead identified additional papers relevant to those testing modalities for which we did not have data from our own bottom-up cost analyses from a separate review of per-patient costs of HIV interventions in South Africa [5] as well as from an existing database of unit costs that we had compiled for the South African HIV Investment Case [6]. Three papers on economic evaluations of home-based testing [7], mobile testing [8] and assisted partner notification [9]

were identified this way and used in the construction of the per-patient costs of these services (Section 2.5.4).

2. Methods

2.1 Methods overview

The epidemiological model used in this analysis is MicroCOSM, an agent-based model developed for South Africa [10]. MicroCOSM is an extension of an agent-based model developed to simulate the spread of HIV and other STIs in South Africa [11]. The model simulates a nationally representative sample of the South African population, starting in mid-1985, allowing for changes in population size over time, as a result of births and deaths. The model does not simulate migration, and is therefore not a fully realistic representation of South Africa's demographic profile. Fertility rates, non-HIV mortality rates and initial population sizes – stratified by age, sex and race – are assumed to be the same as those assumed in the ASSA2008 model, developed by the Actuarial Society of South Africa [12]. The starting population size is set to 20 000 individuals, and the population profile is updated at weekly time steps.

Figure S2.1 summarizes the variables that are assigned to each individual in the model. Each individual is assigned a date of birth, sex and race. Based on these demographic variables, individuals are assigned to various socio-economic states, which are dynamically updated. The socio-economic variables include highest educational attainment, current schooling, urban/rural location, migrant status and incarceration. Demographic and socio-economic variables are in turn assumed to affect uptake of healthcare (hormonal contraception, condoms, HIV testing, ART, pre-exposure prophylaxis and male circumcision). Each individual is also assigned a number of sexual behaviour variables, which include their sexual experience, propensity for concurrent partners ('low risk' individuals are defined in the model as individuals who have no propensity for concurrent partners), marital status, number of current partners, and sexual preference (each male is assigned a male preference value, which can vary between 0 and 1, the latter representing men who are exclusively homosexual). The model also records, for each individual, the identifiers of their current partners, so that individuals can be linked in a sexual network. Individuals are assumed to choose their sexual partners in a highly assortative manner, i.e. tending to select partners of the same educational attainment, race, location, risk group and age group. Based on their sexual behaviour and uptake of healthcare/prophylaxis, individuals are assigned risks of acquiring HIV and other STIs, as well as (in the case of women) risks of falling pregnant. Each HIV-positive individual in the population is assigned an HIV viral load and CD4 count, which determine their risk of transmitting HIV and their risk of dying from HIV respectively. A more detailed description of the model is provided elsewhere [10].

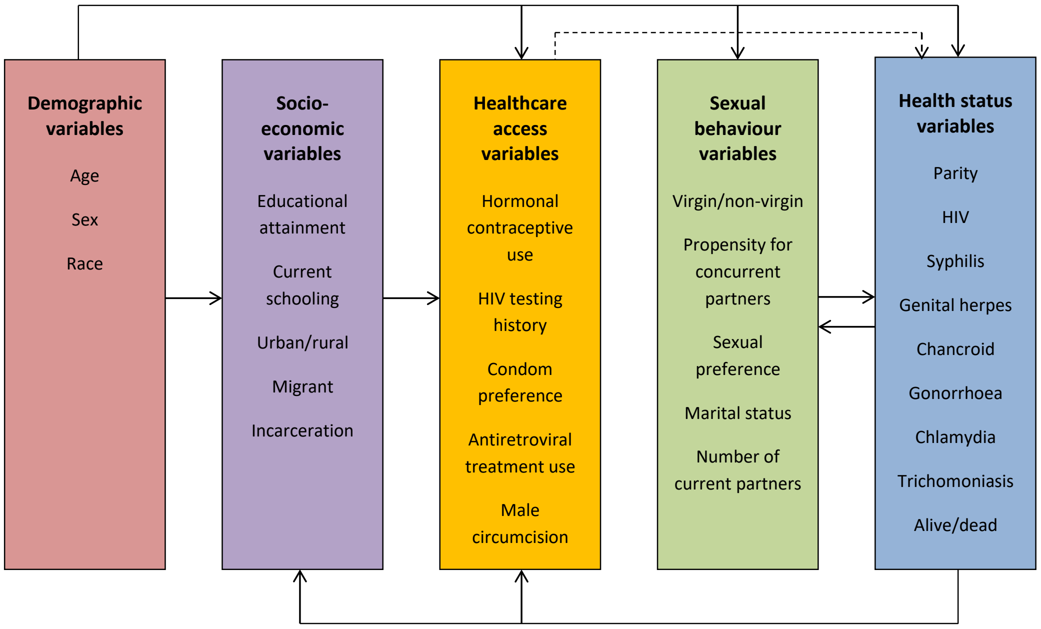

Figure S2.1: Individual-level variables in MicroCOSM

The model has been fitted to age-specific HIV prevalence data from three national household surveys (the HSRC household surveys conducted in 2005, 2008 and 2012 [13-15]) and national antenatal surveys conducted over the 1997-2015 period [16], as well as surveys of HIV prevalence in sex workers and MSM. A likelihood function is defined, to represent the goodness of model fit to the data. Prior distributions are specified to represent the uncertainty in 11 of the HIV transmission parameters, and a sample of 48 000 parameter combinations is drawn from these prior distributions. For each parameter combination, the model is run, and the likelihood is calculated. The 100 parameter combinations that yield the highest likelihood value are then used to generate more detailed model outputs. For each of the 100 parameter combinations, the model is run 5 times, to reduce the extent of the stochastic variation in model outputs, giving a total of 500 model runs. Most of the model results presented in this report are calculated as the average of the results from these 500 parameter combinations. Where uncertainty ranges are presented, these are calculated using the standard errors of the results across the 500 simulations. These uncertainty ranges reflect mostly the stochastic uncertainty that is inherent in the use of an agent-based modelling approach, and are not a true reflection of the overall uncertainty in the model parameters.

Figure S2.2 compares the model estimates of HIV prevalence in the general population with those observed in the HSRC household surveys. The model provides a reasonable fit to the data, although some of the model estimates lie outside of the 95% confidence intervals around the survey estimates.

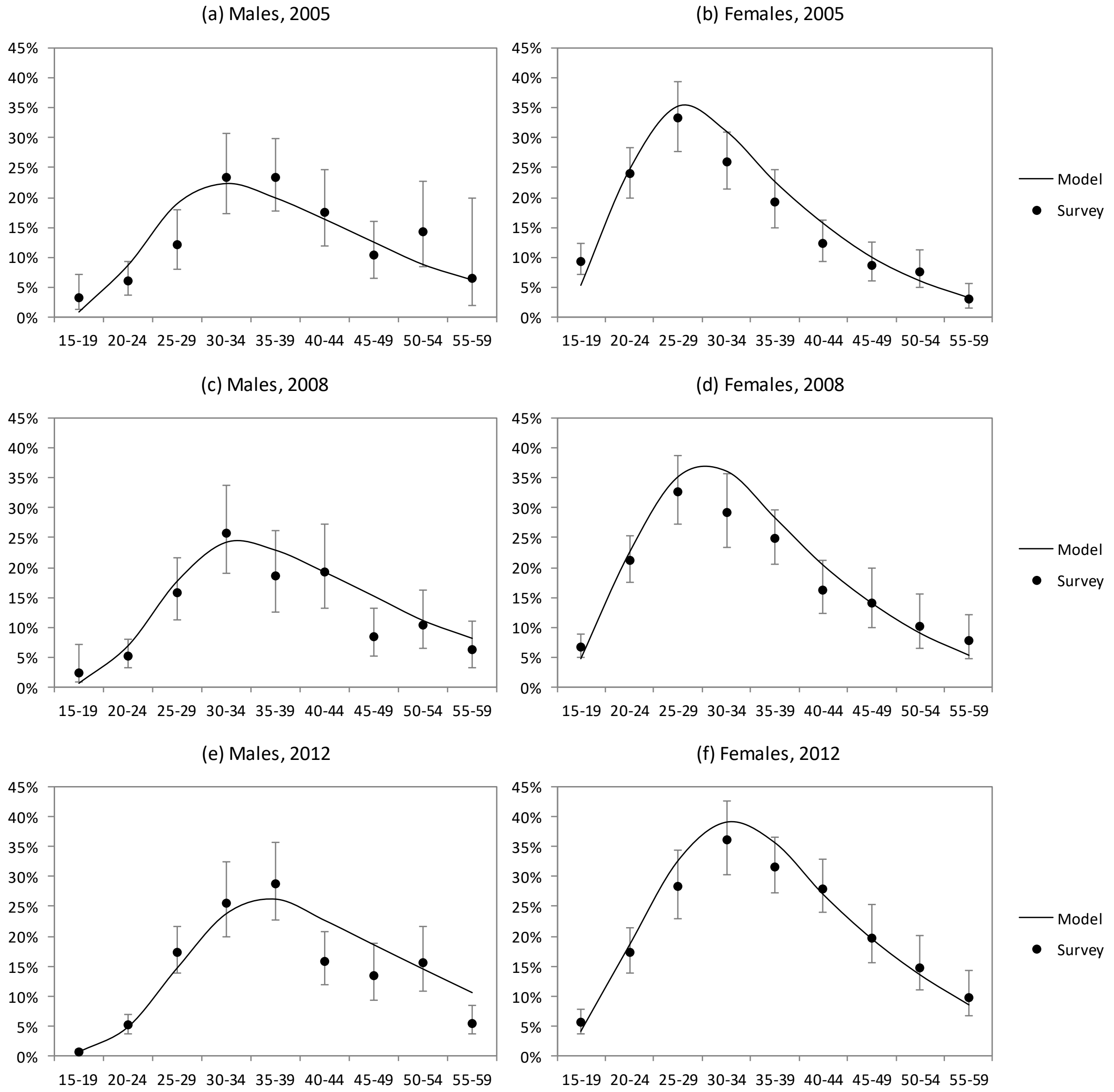

Figure S2.2: HIV prevalence in the general population
Model results are the average results generated using 500 simulations.

Figure S2.3 shows the model calibration to the antenatal HIV prevalence data. Although the model fits the overall antenatal HIV trend reasonably well, the model tends to slightly under-estimate HIV prevalence in women aged 35-39. Antenatal survey results prior to 1997 survey were not used in defining the likelihood function, but are shown for validation purposes: the model tends to over-estimate the pre-1997 prevalence at younger ages (15-24), but under-estimates the pre-1997 prevalence in the 30-39 age group.

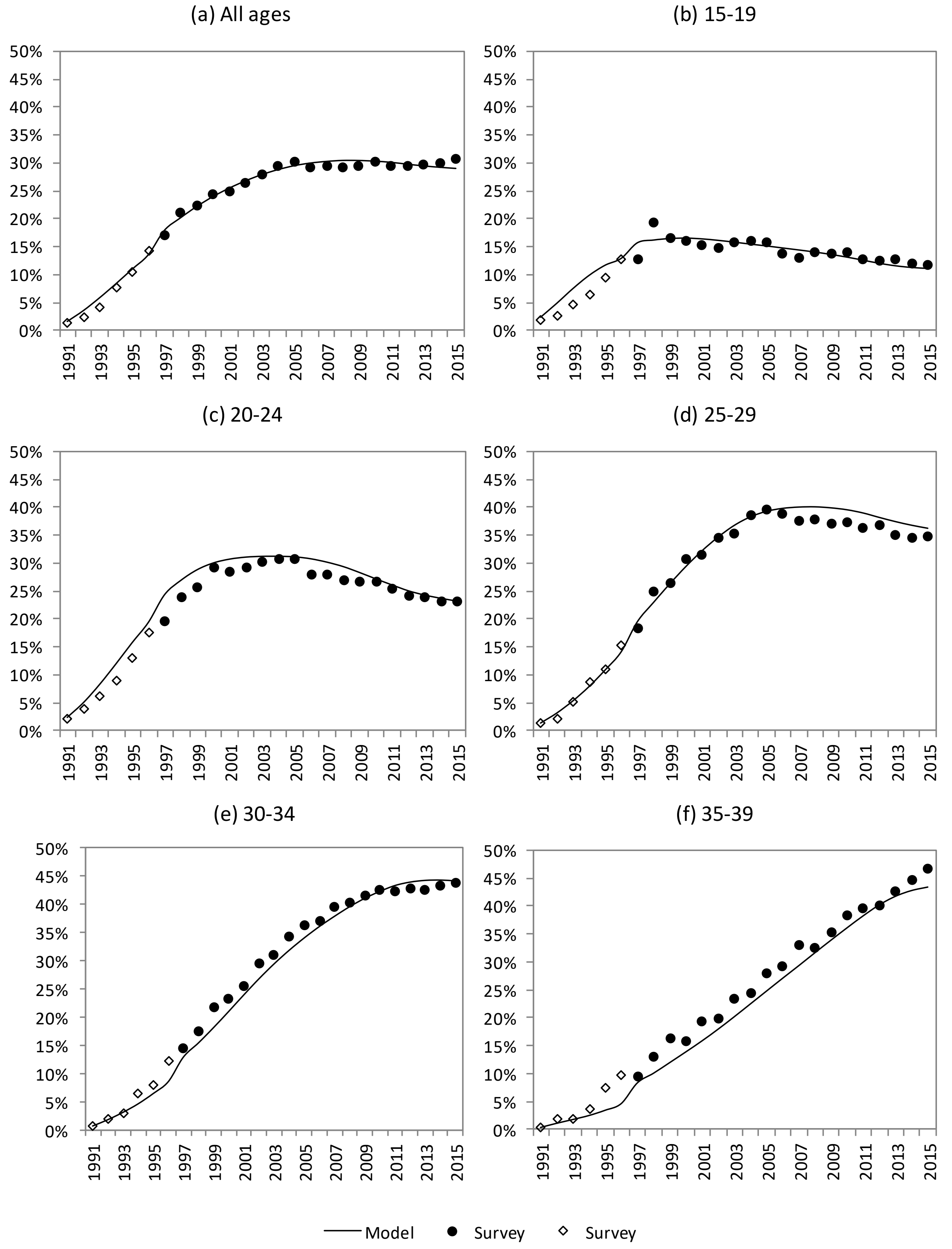

Figure S2.3: HIV prevalence in pregnant women attending public antenatal clinics
Model results have been adjusted to reflect the antenatal biases: the antenatal bias changes in 1997 due to changes in antenatal testing protocol (surveys in 1997 and subsequent years did not include confirmatory testing and some exaggeration due to false positive test results is therefore expected). Model results are the average results generated using 500 parameter combinations.

Figure S2.4 compares the model estimates of HIV prevalence in key populations with the levels of HIV prevalence measured in local surveys. Although the model estimates of HIV prevalence in MSM appear higher than those observed in surveys, the surveys are probably biased towards younger MSM, who have lower HIV prevalence, and the model estimates are roughly consistent with those obtained in a recent analysis that controlled for age differences between modelled and observed MSM populations [17]. Model estimates of HIV prevalence in female sex workers appear roughly consistent with survey data, though slightly higher on average. The average model estimate of HIV prevalence in prisoners, over the 2005-2015 period, was 15.5%, consistent with survey estimates of between 7% and 23% over the 2006-2013 period [18-20].

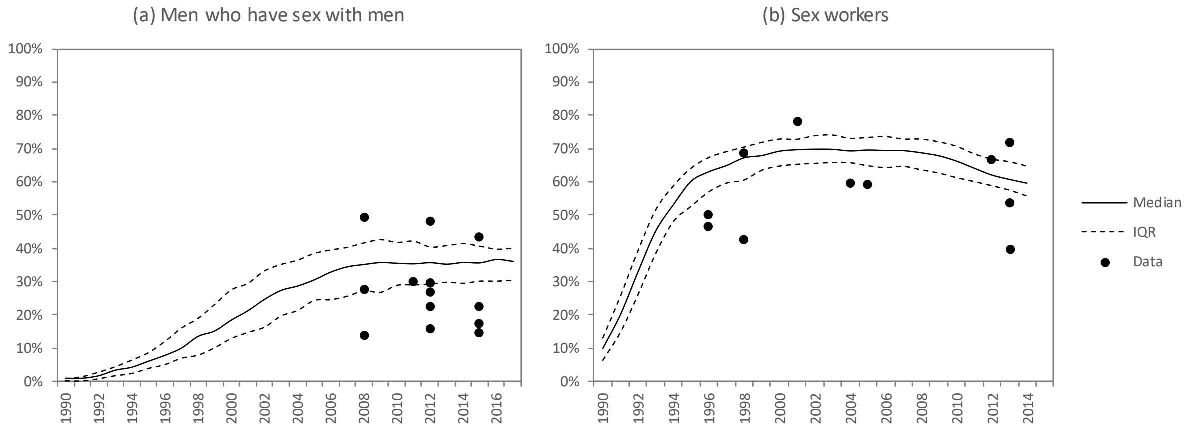

Figure S2.4: HIV prevalence in key populations
Model results are the median HIV prevalence estimates and interquartile ranges (IQRs) generated using 500 parameter combinations.

The MicroCOSM model has been extended to allow for a number of new HIV testing modalities. The model also allows for the possibility that the community-based models of HIV testing may be associated with different probabilities of linkage to ART following diagnosis (compared to existing HIV testing modalities in South Africa). Sections 2.2, 2.3 and 2.5 describe these extensions to the model in more detail.

Where absolute numbers are presented, these are calculated by multiplying the simulated numbers in the sample of the South African population (from MicroCOSM) by the ratio of the size of the South African population in the relevant year (as estimated by the Thembisa model [21]) to the size of the simulated MicroCOSM sample in the year of interest.

2.2 Current South African HIV testing modalities

This section describes the modelling of HIV testing strategies that have already been introduced in South Africa (i.e. testing strategies that form part of the 'baseline' scenario).

2.2.1 General HIV testing

General HIV testing refers mainly to self-initiated HIV testing and all other HIV testing not included in the categories outlined below. The approach to modelling general HIV testing is similar to that described in the Thembisa model [22], which is to say that the rate of general

HIV testing is assumed to depend on the calendar year and the individual's age, sex, sexual experience, educational attainment and HIV testing history. Base HIV testing rates are specified for each sex and each year (see Table S2.1). These base HIV testing rates are set in such a way that the model matches the total recorded numbers of HIV tests performed in South Africa in each year (see Figure 1a of the main text), and are also set to match the relative rates of male and female reporting of past HIV testing in household surveys, as estimated using the Thembisa model [22]. The base testing rates prior to 2002 are estimated by interpolating linearly between a zero rate in 1990 and the rate estimated in 2002, due to the lack of data on routine HIV testing prior to 2002.

The base HIV testing rates are assumed to apply at age 25. Multiplicative age adjustment factors are applied to these base rates in calculating the rates that apply at other ages. A gamma probability density function is used to represent the relative rate of HIV testing at different ages; in males, the mean and standard deviation of the gamma distribution are set at 37.2 and 28.0 respectively, while in women the mean and standard deviation are set at 22.3 and 19.4 respectively, the values estimated when the Thembisa model was fitted to age- and sex-specific HIV testing data in South Africa [22].

The base HIV testing rates are assumed to apply in individuals whose highest educational attainment is grade 11. For each additional grade completed, the rate at which individuals seek general HIV testing is assumed to increase by a factor of 1.12. (For example, the rate of HIV testing in someone who has completed grade 8 would be 0.71 times (1.12^{-3}) the base rate that applies in an individual who has completed grade 11.) The factor of 1.12 is the estimated effect of educational attainment on the uptake of HIV testing by black South Africans between 2010 and 2012, after controlling for age, sex and other factors, which was estimated using data from nationally-representative surveys [23].

General HIV testing is assumed not to occur in youth who are not yet sexually experienced, but apart from this, no effect of sexual risk behaviour on the uptake of general HIV testing is assumed. These assumptions are consistent with South African studies. For example, in a recent study of youth in Western Cape and Mpumalanga, those who reported being sexually experienced were 4.7 times (95% CI: 3.1-7.1) as likely to report having tested for HIV as those who were virgins, after controlling for age and other factors (Franziska Meinck, personal communication). Similarly, in a household survey conducted by Venkatesh *et al* [24], virgins were significantly less likely to report previous HIV testing than youth who were sexually experienced, after controlling for age and other factors. However, among youth who were sexually experienced, the lifetime number of sexual partners was not a significant determinant of the individual's previous testing, either in men or women. Most other South African studies have also not found significant associations between HIV testing history and numbers of partners [25-27].

The base rates apply to individuals who have never tested for HIV before. Individuals who have previously been tested for HIV (and tested negative) are assumed to have a higher rate of HIV testing than individuals who have never previously sought testing, with a multiple of 1.97 being applied to the base rates. This multiple is the same as that estimated when the Thembisa model was fitted to South African HIV testing data [22], and is consistent with various South African studies that show higher rates of testing uptake in previously-tested individuals compared to previously-untested individuals [28-30]. It is assumed that individuals who have previously been diagnosed positive might also get retested, either because they seek

confirmation of the positive test result or because they need to test again in order to link to HIV care. The relative rate of retesting in previously-diagnosed untreated individuals is set to 0.5, based on model calibration to observed trends in HIV prevalence among adults testing for HIV (Figure 1b of the main text). In treated individuals, this relative rate is multiplied by a further adjustment factor to take into account the likely lower rate of retesting in treated individuals: this relative rate is 0.15, based on the Thembisa model [31].

Mathematically, the rate of HIV testing through general HIV testing is calculated as

$$b_g(t)\, A_g(x,t)\, E^h\, r_i\, \theta_a,$$

where $b_g(t)$ is the 'base' rate of general testing in year t, in individuals of sex g aged 25 whose highest educational attainment is grade 11, $A_g(x)$ is the adjustment factor to account for age (x), E^h is the adjustment factor to account for highest educational attainment (h), r_i is the adjustment to take into account HIV testing history (i) and θ_a is the adjustment factor to take into account ART status (a). The E parameter has been set to 1.12. The r_i values have been set to 1 for individuals who have never been tested (i = 0), to 1.97 for individuals who have previously been tested but not diagnosed positive (i = 1), and to 0.5 for individuals who have previously been diagnosed positive (i = 2). The θ_a values have been set to 1 for individuals who are not on ART (a = 0), and to 0.15 in the case of individuals on ART (a = 1). The age adjustment factor is of the form

$$A_g(x) = \left(\frac{x}{25}\right)^{\alpha_g - 1} \exp\left(-\lambda_g (x - 25)\right),$$

where α_g and λ_g can be considered the parameters of a gamma density, with corresponding mean and standard deviation of α_g/λ_g and $\alpha_g^{0.5}/\lambda_g$ respectively. As noted previously, the means have been set to 37.2 and 22.3 years for men and women respectively, while the standard deviations have been set to 28.0 and 19.4 years for men and women respectively.

2.2.2 Testing of patients with HIV-related opportunistic infections

The CD4 counts of HIV-positive adults are dynamically updated, and the incidence of HIV-related opportunistic infections (OIs) is assumed to be a function of the individual's CD4 count and receipt of ART. The annual incidence of OIs in untreated HIV-positive adults is assumed to be 0.05 per annum during acute HIV infection, 0.08 per annum after acute infection in individuals with CD4 counts of 350 cells/µl or higher, 0.27 per annum in individuals with CD4 counts of 200-349 cells/µl and 0.90 per annum in individuals with CD4 counts <200 cells/µl. These rates are based on studies of the incidence of OIs in HIV-positive adults in sub-Saharan African settings [32, 33], and are the same as the values assumed in the Thembisa model [22]. The incidence of HIV-related OIs in HIV-negative adults has been set to 0.019 per annum; this assumption is derived by dividing the annual incidence of TB in the HIV-negative South African population (0.004 per annum [34]) by the fraction of OI patients that have TB at high CD4 counts (20.8% in a Cape Town study [32]). The incidence of OIs in ART patients is assumed to be 0.10 per annum, which is 0.11 times the rate in untreated patients with CD4 <200 and 0.37 times the rate in untreated patients with CD4 200-349. These ratios are roughly consistent with studies that have shown the incidence of HIV-related morbidity after ART

initiation to be between 0.16 and 0.39 times that in untreated patients, after controlling for differences in baseline CD4 count [35-37].

Few data exist on the proportion of OI patients who are tested for HIV, except in the case of TB. It is therefore assumed that the fraction of OI patients tested for HIV is the same as the rate of HIV testing in patients with TB symptoms. Table S2.1 summarizes the model assumptions about the fraction of OI patients tested for HIV and the associated data sources. The model also allows for the possibility that individuals who have previously been diagnosed may get retested when they experience OIs, and the relative rates of testing in previously-diagnosed individuals are the same as assumed in the previous section.

Table S2.1: Assumed rates of general testing and testing in OI patients and pregnant women (undiagnosed)

Year	General testing, $b(t)$		Testing of OI patients		Testing of pregnant women		Retesting of HIV-negative pregnant women	
	Male	Female	Rate	Sources	Rate	Sources	Rate	Sources
Pre-1999	-	-	5%		0.0%		0%	
1999-00	0.0347	0.0558	5%		0.9%		0%	
2000-01	0.0365	0.0586	5%		2.9%		0%	
2001-02	0.0374	0.0602	5%		7.5%	[38]	0%	
2002-03	0.0390	0.0626	5%		15.6%	[39, 40]	0%	
2003-04	0.0308	0.0506	5%		31.3%		0%	
2004-05	0.0228	0.0384	8%	[41]	42.0%	[42]	0%	
2005-06	0.0263	0.0453	15%*	[43]	54.5%	[44]	0%	
2006-07	0.0265	0.0467	21%*	[43]	72.2%	[45]	0%	
2007-08	0.0241	0.0435	29%*	[43, 46]	84.0%	[47]	5%	[48]
2008-09	0.0382	0.0707	34%*	[43, 46]	89.0%	[49]	15%	
2009-10	0.1477	0.2803	43%*	[43, 50]	93.0%		25%	[51]
2010-11	0.1982	0.3861	55%*	[50, 52]	97.0%	[53]	35%	[51]
2011-12	0.1550	0.3020	78%*	[50]	98.0%	[54]	45%	[51]
2012-13	0.1472	0.2867	83%*	[50]	98.0%		55%	
2013-14	0.1043	0.2031	87%*	[50]	98.0%		65%	
2014-15	0.1199	0.2336	92%*	[50]	98.0%		75%	
2015-16	0.1677	0.3268	93%*	[50]	98.0%		85%	
2016-17	0.1851†	0.3605†	93%*⁑		98.0%⁑		95%‡	

* Assumed rates are slightly lower than those quoted in the cited sources because the sources present the fraction of TB patients with a known HIV status, which is higher than the fraction of TB patients who receive an HIV test. † Annual rates of testing for 2017-18 and subsequent years are set to 0.1448 and 0.2821 for men and women respectively, the average of the values estimated over the 2012-2017 period. ⁑ Rates are assumed to remain constant at the 2016/17 level after 2017. ‡ The proportion is assumed to remain at 95% in 2016 and all subsequent years, based on recent unpublished DHIS data, which suggest that almost all HIV-negative pregnant women receive retesting in late pregnancy.

2.2.3 Testing of pregnant women

The modelling of fertility is described in more detail elsewhere [10]. Briefly, each woman's probability of conception is assumed to be a function of her sexual activity (highest in women with multiple partners, and zero in women who are not sexually active), contraceptive usage, breastfeeding and HIV stage (women in more advanced stages of HIV disease being less likely to fall pregnant). The model also allows for variation between women in their natural fecundability, both intra-individual variation (due to changes in hormone levels around menarche and menopause) and inter-individual variation (i.e. allowing for the possibility that

some women are naturally more fertile than others). The modelled conception probabilities are constrained such that the model matches the rates of fertility estimated in the ASSA2008 AIDS and Demographic model [12], by age, race and calendar year.

Table S2.1 shows the assumed proportions of pregnant women tested for HIV in each year. These proportions apply to all women who have not previously been diagnosed positive. As before, the model allows for the possibility of retesting in pregnant women who have previously been diagnosed, and the adjustment factors are the same as described in previous sections.

In recent years it has become common for women who test negative on their first antenatal HIV test to be offered retesting in late pregnancy. Although these retests are usually not included in the total numbers of HIV tests reported by the Department of Health, they do nevertheless affect the cost-effectiveness of antenatal HIV screening and are therefore important to model. Table S2.1 shows the assumed fraction of women testing HIV-negative antenatally who receive retesting in late pregnancy.

2.2.4 Testing of partners of diagnosed individuals

Currently in South Africa, there is no policy of active partner notification when an HIV diagnosis is made. Although newly-diagnosed individuals may be encouraged by health workers to disclose their HIV status to their sexual partner(s), the health worker makes no attempt to contact these sexual partners on behalf of the patient. This ‘passive’ approach to partner notification is generally not shown to be very effective in getting partners of newly-diagnosed individuals tested for HIV. Many South African studies have shown that HIV-diagnosed individuals do not disclose their HIV status to their sexual partners, with disclosure being particularly uncommon in the case of non-spousal relationships [55-57]; it has also been found that rates of disclosure are relatively low in women and in individuals who have not yet initiated ART [56, 58-60]. Even when individuals disclose their HIV status, there is no guarantee that this will result in their sexual partner seeking HIV testing.

Table S2.2 summarizes the results of African studies that have assessed the proportion of sexual partners who come for HIV testing following HIV diagnosis of the index patient. With the exception of the studies of Brown *et al* [61] and Kiene *et al* [62], all of these studies have been done in the context of antenatal screening, and there is thus limited African data on the success of partner notification when the index patient is male. The weighted average fraction of partners receiving HIV testing was 30% (after weighting by the number of index cases in each study). It is also important to note that all of these studies have been conducted in settings in which a relatively high proportion of index patients are married or cohabiting with their sexual partner. In two of the studies, there was a strong association between male partner testing and marital status (OR 4.50 (95% CI: 2.20-9.19) in Tanzania [63] and OR 2.11 (95% CI: 1.14-3.92) in Kenya [64]).

Table S2.2: Proportion of partners coming for HIV testing following diagnosis of index HIV case (passive partner notification approaches)

Study	Location	% married	# individuals diagnosed	% of partners seeking testing
Msuya *et al* [63]	Moshi, Tanzania	91%	184	18.5%
Aluisio *et al* [64]	Nairobi, Kenya	84%*	456	30.7%
Brown *et al* [61]	Lilongwe, Malawi	73%	82	24.4%
Rosenberg *et al* [65]	Lilongwe, Malawi	100%	100	52.0%
Kiene *et al* [62]	Mpigi, Uganda	-	38†	35.1%

* Monogamous marriages only. † The number of partners of newly-diagnosed individuals.

In our model, the probability of HIV disclosure at the time of HIV diagnosis is assumed to depend on the relationship type and the individual's sex. A disclosure probability of 50% is assumed for women who are in non-marital relationships, and this disclosure probability is adjusted by odds ratios of 1.25 in men and 2.5 in married couples (i.e. the probability of disclosure from a married woman to her husband is 71%, and the probability of disclosure from an unmarried man to his partner is 55%). If 30% of the husbands of newly-diagnosed married women receive HIV testing following their wives' HIV diagnosis, this implies that the probability of men seeking HIV testing, given that their wives have disclosed their HIV-positive status, is 0.42 (i.e. 0.30/0.71). In the absence of more detailed data, the same 0.42 probability of seeking testing (conditional on disclosure) is assumed to apply regardless of whether the partner is male or female, and regardless of whether the relationship is marital or non-marital. For example, if an unmarried woman is diagnosed positive, the probability that she discloses her HIV status to her male partner and he gets tested as a result of this disclosure is $0.50 \times 0.42 = 0.21$.

2.2.5 Testing of STI patients

Early syndromic management protocols in South Africa did not explicitly mention the need for HIV testing as part of STI patient consultations [66, 67], and the importance of HIV testing has only been mentioned in guidelines issued since 2009 [68]. Few data exist on the extent to which HIV testing is actually offered. The only nationally representative data are from surveys conducted in 2002 and 2014, in which standardized patient actors were used to estimate that 8% and 67% of providers respectively offered HIV testing to STI patients [39, 69]. A longitudinal study in North West province in 2013 found that 54% of standardized patient actors were offered HIV testing at baseline [70]. Earlier studies in Cape Town and KwaZulu-Natal found intermediate proportions: Leon *et al* conducted a trial and found that in non-intervention STI clinics, the rate of HIV testing increased from around 30% prior to 2007 to 43% in 2007 [71], and the latter was similar to the rate found in KwaZulu-Natal in 2006 [72]. Table S2.3 shows the assumed proportions of STI patients receiving HIV testing in each year, based on these studies. These rates of HIV testing are assumed to apply only in the public health sector, as the only available data are from the public sector. The limited data available from the South African private sector suggest that relatively few private practitioners follow syndromic management protocols [73], and thus few would be expected to offer HIV testing as part of the STI patient consultation.

Table S2.3: Assumed HIV testing rates in public-sector STI patients and prisoners, and rates of medical male circumcision (MMC)

Year	Testing of STI patients		Ratio of # MMC operations	Testing of prisoners	
	Proportion	Sources	to # men aged 15-49	Annual rate	Sources
Pre-2000	0%		0.0000	0.0	
2001-02	3%		0.0000	0.0	
2002-03	8%	[39]	0.0000	0.0	
2003-04	13%		0.0000	0.0	
2004-05	18%		0.0000	0.0	
2005-06	24%		0.0000	0.05	
2006-07	30%	[71]	0.0000	0.10	
2007-08	43%	[71]	0.0000	0.19	[74]
2008-09	47%		0.0004	0.29	
2009-10	51%		0.0007	0.39	[75]
2010-11	55%		0.0098	0.49	
2011-12	58%		0.0258	0.58	[76]
2012-13	61%		0.0309	0.68	[76]
2013-14	64%	[70]	0.0240	0.97	[77]
2014-15	67%	[69]	0.0363	1.13	[78]
2015-16	69%*		0.0363*	1.13*	

* Rates are assumed to remain constant at the 2015/16 level after 2016.

Our model simulates the incidence of five STIs in addition to HIV: syphilis, genital herpes, gonorrhoea, chlamydia and trichomoniasis. In addition, the model simulates the incidence of vaginal candidiasis and bacterial vaginosis in women. For each of these infections, an assumed proportion of cases becomes symptomatic, with individuals experiencing symptoms of genital ulceration or discharge. Symptomatic individuals are assumed to seek treatment at a weekly rate, with a proportion of these cases seeking treatment in public health facilities. Assumptions regarding STI treatment seeking are described in more detail elsewhere [79], and the model calibration to South African STI prevalence data is also described elsewhere [11]. The model simulates HIV testing in STI patients by applying the testing probabilities in Table 2.3 to the weekly rates of seeking treatment in public health facilities, in individuals who have any symptoms of genital ulceration or discharge. A limitation of this modelling approach is that it does not consider other conditions that could prompt patients to seek treatment at STI clinics (for example, human papillomavirus infections, which cause genital warts). The model allows for retesting in STI patients who have previously been diagnosed, based on the same adjustments as described in section 2.2.1.

2.2.6 Testing of men who seek medical male circumcision (MMC)

It is standard practice for men who get medically circumcised to receive HIV testing prior to circumcision. WHO guidelines on MMC recommend HIV testing prior to circumcision [80], and most donor-supported programmes also recommend this. For example, Lesotho's MMC programme, supported by USAID, has provided HIV testing to 97% of all men presenting for MMC [81], and in a South African study, almost all men seeking MMC received HIV testing [82]. South African studies also show a strong association between MMC and knowing one's HIV status. For example, in a sample of South African men who had been circumcised, Jean *et al* [83] found that 24% of those who had received an HIV test reported that the most recent HIV test was at the time of undergoing MMC. The sample included men who had been

traditionally circumcised (i.e. not in health facilities) and thus the proportion would probably be much higher than 24% if the analysis was restricted to men who had received MMC.

Assumptions about the annual rate of MMC uptake in uncircumcised men are based on the most recent version of the Thembisa model [84]. In the Thembisa model, the uptake of MMC is modelled based on published numbers of MMC operations in annual reports of the Department of Health [85-87]. Uptake is assumed to be a function of age, based on the reported age distribution of MMC operations [88]. In MicroCOSM, we use the Thembisa estimates of the number of MMC operations per male aged 15-49 in each year, as model inputs (see Table S2.3), and also use the Thembisa estimates of the relative rates of MMC uptake by age to determine the age-specific rates at which uncircumcised men get medically circumcised. Relative rates of MMC (relative to ages 10-14) are set to 0.60 for males aged 15-19 years, 0.35 for 20-24, 0.27 for 25-29, 0.21 for 30-34, 0.16 for 35-39, 0.12 for 40-44, 0.08 for 45-49, 0.05 for 50-54, 0.03 for 55-59, 0.02 for 60-64, 0.01 for 65-69 and zero for males aged 70 and older. This implies that the majority of MMC operations occur in adolescent boys.

It is assumed that only men who have not been diagnosed positive would seek MMC, and that all men who seek MMC are tested for HIV. In the event that men are diagnosed positive on seeking MMC, it is assumed that they choose not to get circumcised. (Although protocols do not restrict MMC to men who are HIV-negative, it is assumed that HIV risk reduction is the primary reason for seeking MMC, and thus a man who is diagnosed HIV-positive would have less incentive to get medically circumcised.)

2.2.7 Testing of men in prison

MicroCOSM simulates male incarceration and release from prison, assuming that rates of incarceration depend on men's age, educational attainment, race, and history of prior incarceration. The model has been calibrated to match the estimated fraction of the male population in prison and the age profile of incarcerated men. A more detailed description of the model of incarceration is provided elsewhere [10].

HIV testing is commonly offered to prisoners at the time of admission to prison. In addition, prisoners are often able to request HIV testing at other times. The Department of Correctional Services only publishes the total number of HIV tests performed in prisons each year (i.e. with no split between new prisoners and those who have been incarcerated for longer durations), and we therefore model HIV testing in prisons as occurring at an annual rate, which is independent of the time since entry into prison. The model assumption about the rate of testing in a given year is obtained by dividing the reported total number of HIV tests in each year by the estimated number of prisoners in that year. The resulting assumed rates of testing are shown in Table S2.3. Relative rates of retesting in previously-diagnosed prisoners are the same as those assumed for general testing (see section 2.2.1).

2.2.8 Testing of sex workers and MSM receiving PrEP

A policy of free provision of PrEP for South African sex workers was announced in March of 2016, but there has to date been very limited uptake among sex workers. In a South African PrEP demonstration project, only 219 out of 351 HIV-negative sex workers started PrEP, and only 117 returned for their first visit 1 month after baseline [89], suggesting an effective adoption rate of 33% (117/351). By the end of December 2017, approximately 3000 sex

workers had started PrEP in South Africa, 1.5 years after the South African sex worker PrEP programme was introduced (Hasine Subedar, personal communication). This is approximately 8% of the Thembisa estimate of the number of HIV-negative sex workers in South Africa. We assume that from mid-2016 the mean annual rate at which sex workers adopt PrEP is 0.07.

There is limited local data on PrEP uptake among MSM, as this only became policy in April of 2017. To be consistent with the assumptions made about PrEP uptake in sex workers, we set the assumed annual rate of PrEP uptake in MSM to be 0.07 per annum from July of 2017.

It is assumed that all sex workers and MSM will be tested for HIV prior to initiating PrEP, and that those who test positive will not be started on PrEP. South African guidelines recommend quarterly screening of all individuals receiving PrEP [90], and it is therefore assumed thatindividuals receiving PrEP are tested for HIV every three months. Those who test positive are assumed to discontinue PrEP immediately.

2.3 New HIV testing strategies

This section describes potential new HIV testing strategies that could be introduced in South Africa. Although many of these new testing strategies have been piloted in small studies, none have been rolled out on a national scale. For each new testing strategy, we describe the model assumptions and the data sources on which they are based.

Although assumptions about relative rates of testing in previously-diagnosed HIV-positive individuals are fixed for the HIV testing modalities that have already been introduced in South Africa (as described in section 2.2.1), we allow for uncertainty regarding the relative rates of testing for each of the new testing modalities. For the r_2 parameter (the relative rate of testing in diagnosed untreated individuals, when compared to individuals who have never been tested), we assign a uniform (0, 1) prior to represent the uncertainty, i.e. assuming that diagnosed individuals would be less likely to accept an HIV testing offer than individuals who have never been tested. For the θ_1 parameter (the relative rate of testing in treated individuals compared to diagnosed untreated individuals), we assign a beta prior with a mean of 0.36 and a standard deviation of 0.18, based on a Mozambican study that found significantly lower uptake of HIV testing in ART patients when compared to HIV-diagnosed untreated individuals, in the context of home-based testing [91].

2.3.1 Assisted partner notification

Assisted partner notification differs from ‘passive’ partner notification in that the healthcare provider takes a more active role in facilitating disclosure of HIV status to sexual partners and referral of partners for HIV testing. Two trials in Malawi examined the effect of assisted partner notification compared to the traditional passive approach. Rosenberg *et al* [65] examined the effect of contract referral, i.e. women in the intervention arm agreed that if their partner did not come to the clinic for testing within the next week, a community health worker would trace them and invite them for HIV testing. This study found that the rate of partner notification in the contract referral arm was 74%, compared to 52% in the passive partner notification arm. The second trial tested two different forms of assisted partner notification: contract referral and provider referral (in the latter case, the health worker contacted the partner immediately and invited them for HIV testing) [61]. The rate of partner referral in the two intervention arms was

51% (the same rate in the contract and provider referral arms) compared to 24% in the control arm. Pooling the results of these two trials gives a combined OR of 2.93 (95% CI: 1.87-4.58) for the effect of assisted partner notification on rates of partner referral.

In a more recent trial of provider referral in Kenya, Cherutich *et al* [92] found that the fraction of partners who received HIV testing within 6 weeks was 65% in the intervention arm compared to only 13% in the control arm. It is not clear why the rate of HIV testing in the control arm was so low when compared with the average of 30% from Table S2.2, but it may be because control arm recipients were told that their partners would be contacted regardless of which arm they were in – the only difference in the control arm being that contact occurred after a 6-week delay. This might have meant that individuals in the control arm had less incentive to disclose their HIV status to sexual partners. For this reason we have not pooled the result of this trial with the results of the two Malawian trials. Another study found that 56% of male partners were tested through a provider referral intervention in Cameroon, but this study did not include a control arm [93]. A recent meta-analysis of the results from the three randomized trials also estimated a significant effect of assisted partner notification on partner testing (RR 1.46, 95% CI: 1.22-1.75), though the effect was small because it was expressed as a rate ratio rather than an odds ratio [94].

We model the effect of assisted partner notification by applying an odds ratio to the assumed rates of partner notification specified in section 2.2.4. For example, if the proportion of husbands of newly-diagnosed women who come for HIV testing is 30% in the absence of active partner notification, and assisted partner notification is assumed to increase the odds of partner testing by a factor of 3, then the fraction of husbands of newly-diagnosed women who would come for HIV testing under assisted partner notification is 0.56, i.e. 3 = (0.56/(1 – 0.56))/(0.30/(1-0.30)). To represent the uncertainty around the odds ratio, we assign a gamma prior with a mean of 3 and a standard deviation of 0.7, which has 2.5 and 97.5 percentiles of 1.79 and 4.51 respectively. This confidence interval corresponds roughly to confidence interval around the pooled odds ratio of 2.93 (1.87-4.58). Although the model allows for assisted partner notification to change the proportion of partners who are tested, it is assumed not to change the probability that the index case discloses their HIV status to their partner, and thus the only parameter that changes is the probability of partner testing *conditional* upon disclosure. In the example, this conditional probability increases from 0.42 (0.30/0.71) in the absence of assisted partner notification to 0.79 (0.56/0.71) with assisted partner notification.

2.3.2 Home-based testing

Several pilot studies have been conducted to assess the uptake of home-based HIV testing in sub-Saharan Africa. Although the majority of these studies show that a high proportion of the individuals reached through home-based testing consent to testing [95-97], relatively few studies report the fraction of the population reached through home-based testing, or the rate at which individuals are reached [98]. Some studies report the number tested both as a fraction of those offered testing and as a fraction of those in the population (Table S2.4). The latter is substantially lower because many household members are not present at the time of the visit of the HIV testing team. These findings are consistent with a recent systematic review and meta-analysis of home-based testing interventions in sub-Saharan Africa, which estimated average uptake of 82% (95% CI: 76-87%) but average coverage of only 70% (95% CI: 58-79%) when absent household members were included in the denominator [99].

Table S2.4: Comparison of home-based testing coverage levels using different coverage definitions

Study	Location	Uptake of testing	
		As % of those offered	As % of population
Parker *et al* [100]	Shizelweni region, Swaziland	73.6%	52.6%
Tumwebaze *et al* [101]	Kabwohe district, Uganda	98.1%	80.2%
Sekandi *et al* [102]	Kampala, Uganda	69.4%	58.0%
Bogart *et al* [103]	Kavenyanja Island, Uganda	88.3%	60.3%
Shanaube *et al* [104]	Zambian communities in PopART trial	72.2%*	59.0%*

* Excluding individuals who self-reported a prior HIV-positive diagnosis.

Some test refusal occurs because HIV-positive individuals already know their HIV status or because uninfected individuals have recently tested. For example, the coverage estimate of 60.3% in Kavenyanja Island increased to 66.3% if individuals who already knew they were HIV-positive were excluded [103]. In a South African study, 41% of individuals who refused the offer of home-based HIV testing did so because they already knew their HIV status [95]. Thus the rates of uptake in Table S2.4 could be under-estimates of rates of test uptake in undiagnosed individuals.

Men and women appear to have different rates of HIV testing. The previously-cited meta-analysis of studies of home-based testing in Africa estimated that men comprised only 40% of individuals tested for HIV [99], which suggests higher rates of uptake in women compared to men. If it is assumed that there are on average equal numbers of men and women, this suggests an HIV testing rate in men that is two thirds of that in women. We therefore assume that the rate of uptake of home-based testing is 1.2 times the average in women and 0.8 times the average in men (so that the male-to-female testing rate is 2/3). To represent the uncertainty regarding the average uptake (in undiagnosed individuals), we assign a beta distribution with a mean of 70% and a standard deviation of 7%. This distribution has 2.5 and 97.5 percentiles of 55.5% and 82.7% respectively; the lower bound is close to the lowest uptake estimate in Table S2.4 (58%) and the upper bound corresponds to the maximum rate of 100% for women (since 82.7% × 1.2 = 99.2%). The mean of the distribution is the same as that estimated in the meta-analysis of home-based testing studies [99].

Age is assumed to have no effect on the uptake of home-based testing [103, 105], although two studies in Malawi and Zambia found that uptake of home-based testing was significantly lower in older adults [104, 106].

The likely frequency of home-based testing is unclear. Although modelling studies have typically assumed very frequent household HIV testing (for example, once a year or more frequently [107, 108]), studies in South Africa have had variable success in achieving rapid coverage. In a cluster-randomized trial of home-based testing in rural KwaZulu-Natal, Doherty *et al* [98] found that it took 14 months to visit all households in the intervention clusters. In another cluster-randomized trial of home-based testing in rural KwaZulu-Natal, Naik *et al* [95] found that it took 5 months to visit all households in the intervention clusters. Another community-based study in rural KwaZulu-Natal also found that it took 5 months to visit all households [96]. Given that these are research studies or randomized trials, which are relatively well-resourced, one might expect testing teams in field settings to take longer to reach

households. It is also worth noting that almost all of these studies have been once-off evaluations of home-based HIV testing, and only one study has evaluated the uptake of home-based HCT when repeated after an interval of two years [106]. In this study, uptake of HIV testing improved in the second round. Although this may have been partly because of greater efforts to reach absent household members in the second round, the results suggest that biennial home-based testing is feasible without significant deterioration in uptake. We therefore assume that home-based testing teams visit households once every two years on average, and that there is no change in home-based testing uptake over time.

2.3.3 Testing of women attending family planning clinics

Although the most recent South African HIV testing guidelines recommend offering HIV testing to women attending family planning clinics [109], no data exist on the extent to which this guidance is actually followed. It is therefore conservatively assumed that there has been minimal HIV testing performed in family planning clinics to date. Data from other African settings in which HIV testing has been integrated into family planning services generally suggest low HIV test uptake. In a Kenyan study, it was found that 35% of women attending intervention clinics, in which HIV testing was offered as part of family planning consultations, received HIV testing [110]. In another Kenyan study, 28% of women attending family planning services received HIV testing at baseline, but the extent of integration of HIV testing into family planning services varied across clinics [111]. Paradoxically, HIV test uptake at baseline was lower in the family planning clinics in which there had already been substantial integration, and the authors speculated that this might be because there was a degree of saturation, i.e. HIV testing was less likely to be received by women who had already tested at the family planning service. The fraction of women attending the well-integrated clinics who received the recommended testing frequency (at least one test per 12-month period) was 65%, but this is likely to be an upper bound on the fraction of women who actually received the recommended tests in family planning clinics, because women were asked about any HIV testing (not specifically testing in family planning clinics).

For the purpose of modelling the potential future impact of promoting HIV testing in family planning clinics, we assign a gamma prior to represent the uncertainty around the annual rate at which family planning attenders get tested at family planning services. This prior distribution is assigned a mean of 0.45 and a standard deviation of 0.10. The 2.5 and 97.5 percentiles of this distribution are 0.28 and 0.67 respectively; the lower bound thus corresponds to the value that might be expected if women make only one family planning clinic visit per year (since the 28% and 35% estimates are the testing probabilities for a single visit), while the upper bound of 0.67 corresponds to the value that would be expected if all of the women who received annual testing in the Kenyan study were tested at family planning services.

MicroCOSM simulates the uptake of contraception dynamically, assigning to each woman a female-controlled contraceptive use variable (no contraception, injectable contraception, oral contraception or sterilization) and separately assigns to each woman indicators of whether she consistently uses condoms with her current sexual partner(s). The uptake of hormonal contraception is assumed to be lower in women who consistently use condoms. It is also assumed to be influenced by women's age, race, educational attainment, sexual activity (lower in women who are not sexually active), natural fertility (lower in women who are relatively less fertile), pregnancy status and breastfeeding status. The model also allows for a shift towards lower hormonal contraceptive methods over time, particularly in young women, as

condoms have become relatively more popular. Women who use injectable contraception are also assumed to be more susceptible to HIV. A more detailed description of the model of hormonal contraceptive use is provided elsewhere [10]. The women who use hormonal contraception (injectable or oral contraceptives) are assumed to comprise the population of women attending family planning clinics.

2.3.4 Mobile testing

A limitation of many studies of mobile testing is that they do not report information on the demographics of the population in which mobile testing is provided, but report only the characteristics of the individuals tested for HIV. This makes it difficult to determine both the rate of uptake at a population level and the relative rates of uptake by age and sex. Nevertheless, a few studies have provided enough information to calculate an annual rate of HIV testing when mobile HIV testing was offered in a particular community (Table S2.5). A study conducted in a South African township near Cape Town found that when a mobile testing service was introduced in the community over a 16-month period between 2009 and 2010, the annual rate of HIV testing through the service was 5.5 tests per 100 person years [112]. Similarly, when a mobile testing service was offered in the Umlazi township in KwaZulu-Natal, the average HIV testing rate in adults was approximately 2.8 HIV tests per 100 person years [113]. These rates are substantially lower than the rates of 15-19 per 100 person years observed in a randomized trial of mobile HIV testing in three countries [114]. This may be because the randomized trial included a significant community mobilization component. Being a randomized trial may also have meant that the intervention was more intense than might be expected in a 'real world' setting. A high rate of uptake (34 per 100 person years) was also observed in mobile-testing campaign conducted over a short period (5 months) in Tanzania, the high uptake rate a likely reflection of the short duration of the intervention and the community mobilization associated with it [115]. A limitation of all four studies is that they were not able to estimate what fraction of the population was already HIV-diagnosed (and thus unlikely to present for HIV testing); including these diagnosed individuals in the denominator means that the rate of testing in undiagnosed individuals may be under-estimated.

Table S2.5: Annual uptake of HIV testing due to mobile testing

Study	Location	Population	Annual testing rate (per person year)
Kranzer *et al* [112]	South Africa	Ages 15+	0.055
Bassett *et al* [113]	South Africa	Ages 15+	0.028
Sweat *et al* [114]	Tanzania	Ages 16-32	0.15
	Zimbabwe	Ages 16-32	0.16
	Thailand	Ages 16-32	0.19
Ostermann *et al* [115]	Tanzania	Ages 18-50	0.34

Although the Cape Town study did not directly report testing rates stratified by demographic characteristics, information on the demographic profile of the community from another study [116] allows the calculation of these rates. Testing rates were slightly higher in men than in women (6.3 versus 4.8 per 100 person years) and slightly higher in individuals aged 15-34 than in those aged 35 or older (5.8 versus 4.6 per 100 person years), but were the same for individuals who had previously tested for HIV and those who had never tested for HIV before. Another study in Thailand, which did not report testing rates but nevertheless compared the features of individuals who received mobile HIV testing with those who did not, found that the

two groups were similar with respect to age, sex and prior HIV testing, but that individuals who received mobile testing were more likely to be unmarried and to report higher levels of risk behaviour [117]. In a meta-analysis of mobile HIV testing studies in sub-Saharan Africa [99], the average proportion of clients who were male was 50% (95% CI: 47-54%), which suggests similar rates of mobile testing uptake in men and women. In the Tanzanian study described previously, similar numbers of men and women were tested as part of the intervention, but uptake was slightly lower in older adults than in younger adults [115].

In our model we assume that if mobile HIV testing is offered in a community, there is a constant rate of mobile test uptake that is the same for all adults, regardless of age or sex. To represent the uncertainty regarding the annual rate that would be expected in the absence of community mobilization, we assign a gamma prior with a mean of 0.055 (the same as that estimated in the Cape Town study [112]) and a standard deviation of 0.02. We also assume that community mobilization would lead to a doubling of this rate. The 2.5 and 97.5 percentiles of the distributions are 0.023 and 0.101 respectively in the absence of community mobilization (i.e. wide enough to include the estimates from the two South African studies), and 0.046 and 0.202 respectively with community mobilization (wide enough to include the estimates from the RCT of mobile testing with community mobilization). Although it may seem conservative to assume that community mobilization doubles testing uptake, this is more consistent with the modest effects of community mobilization observed in another recent South African study, which found that community mobilization had little effect on HIV testing [118].

2.3.5 HIV testing targeted to MSM

Few studies in developing countries have assessed the impact of mobile HIV testing targeted to men who have sex with men (MSM). In a Nigerian study, Adebajo *et al* [119] evaluated a strategy that involved high-risk men (either MSM or injecting drug users) offering HIV testing to their peers, or else referring them to a nearby mobile testing service. Although it was found that a high proportion of the MSM accepted the offer of HIV testing (99% and 77% respectively), no information was provided on the methods used to locate MSM or the fraction of MSM who were reached by the intervention. This makes it difficult to estimate what levels of coverage are feasible with such an intervention. Another study involved peer education among Kenyan male sex workers (MSWs), and found that MSWs who had been exposed to the peer educators were more likely to report having ever been tested for HIV [120]. After the intervention had been running for a year it was found that the proportion ever tested was 81% among the 33% of MSWs who had been exposed to peer educators, compared to 61% among the MSWs who had not been exposed. Crudely, this suggests an annual uptake of HIV testing among all MSWs of $0.33 \times (-\ln((0.81 - 0.61)/(1 - 0.61))) = 0.22$.

Another MSM-focused intervention in South Africa, Boithato, aimed to recruit MSM in a predominantly rural district into regular HIV testing, provided by MSM-friendly clinics. An RDS survey identified a sample of 185 HIV-negative MSM in the community at baseline [121]. Of these, 121 subsequently tested for HIV as part of the Boithato intervention, over an average duration of 1.2 years [122]. This suggests an annual rate of HIV testing through the intervention of roughly 0.88 ($-\ln(1 - 121/185)/1.2$). This estimate of 0.88 is probably an over-estimate, as MSM who participated in the baseline survey were probably not a representative sample of all MSM in the district, and because they participated in the baseline survey, they may have been more exposed to the HIV testing intervention.

We assign a gamma prior distribution to represent the uncertainty around the MSM testing rate in communities in which interventions similar to Boithato have been introduced. This gamma prior is assigned a mean of 0.40 and a standard deviation of 0.20. The 97.5 percentile of this distribution is 0.88, the same as the upper limit estimated from the Boithato trial, while the 2.5 percentile is 0.11, less than the value of 0.22 estimated in Kenya. This rate is multiplied by the assumed coverage of this testing intervention to obtain the actual uptake in a given year.

2.3.6 HIV testing targeted to sex workers

Although many African studies have assessed the impact of interventions to increase STI screening in sex workers [123], few African studies have examined the uptake of HIV testing among sex workers in programmes that targeted sex workers. Ghys *et al* [124] evaluated the impact of an intervention in Abidjan (Côte d'Ivoire), which involved peer educators recruiting sex workers to a dedicated clinic for HIV testing and STI screening. Independently, sex workers in the community were surveyed to determine whether they had ever accessed this dedicated sex worker service, and the proportion of sex workers who reported having ever accessed the service increased from 9% in 1993 (soon after the service was introduced) to 37% in 1997. This is roughly equivalent to a 15% annual rate of HIV testing (taking into account the competing risk of retirement from commercial sex work).

In the more recent SAPPH-IRe trial, conducted in Zimbabwe, community mobilization was conducted among sex workers in intervention clusters to promote various HIV services, including HIV testing (women who were HIV-negative were encouraged to retest at 6-month intervals) [125]. RDS surveys were conducted at the start and end of the trial, and among HIV-positive sex workers in the intervention clusters, the fraction diagnosed increased from 62.3% in the baseline survey to 79.8% in the final survey, roughly 29 months later. HIV prevalence among sex workers remained relatively stable over the trial, and it is therefore reasonable to assume that HIV-diagnosed sex workers who retired from sex work over the course of the trial were replaced by either (a) new sex workers who were already HIV-positive, or (b) new HIV infections among women who were sex workers at the time of acquiring HIV. For the purpose of estimating the annual rate of HIV testing among sex workers in the intervention clusters, λ, it will be assumed that both sources of 'replacement' HIV infection are initially undiagnosed. It is further assumed that of those 'replacement' infections remaining in the intervention clusters at the time of the final survey, the distribution of times since either acquiring HIV or entering sex work (whichever event occurred later) is uniform over the time between the baseline and final surveys. This means that among these replacement HIV infections, the proportion who are undiagnosed at the time of the final survey is

$$\frac{1}{2.42}\int_0^{2.42} \exp\left(-\lambda(2.42-t)\right)dt,$$

where 2.42 is the time between the first and final surveys (in years). If r is the annual rate at which women retire from sex work, then the proportion of HIV-positive sex workers at the time of the first survey who are still sex workers at the time of final survey should be $\exp(-2.42r)$, and the proportion of these women who remain undiagnosed at the time of final survey should be $\exp(-2.42\lambda) \times (1 - 0.623)$. Thus the overall proportion of HIV-positive sex workers who are undiagnosed at the time of the final survey should be

$$\exp(-2.42r)\exp(-2.42\lambda)(1-0.623)+(1-\exp(-2.42r))\frac{1}{2.42}\int_0^{2.42}\exp(-\lambda(2.42-t))dt.$$

Setting this expression to 1 – 0.798 and fixing r at an assumed retirement rate allows us to solve for λ. However, results are sensitive to the assumed value of r. If $r = 0.33$ (the value assumed in the Thembisa model [126], equivalent to an average duration of 3 years in sex work), the estimated value of λ is 1.11. Changing r to 0.125 and 0.50 (equivalent to average durations of 8 and 2 years respectively) yields λ estimates of 0.60 and 1.41 respectively. These annual rates of HIV testing are substantially higher than the estimates obtained from Côte d'Ivoire.

Another recent study assessed the effect of an intervention to improve sex workers' access to reproductive healthcare (including HIV testing) in three African cities (Durban, Mombasa and Tete), using a variety of context-specific methods [127]. As in the Zimbabwean trial, RDS surveys were conducted prior to the intervention and at the end of the intervention to assess the effect of the intervention on the uptake of HIV testing. The annual testing rate (calculated from the proportion of women who reported HIV testing in the last 6 months) increased from 1.05 to 3.57 in Durban, from 2.47 to 4.17 in Mombasa, and from 1.64 to 2.90 in Tete. These imply increases in annual testing rates of 2.52, 1.71 and 1.26 respectively, i.e. somewhat higher than the Zimbabwe trial results suggest. It is possible that the observed change in testing rates might be due in part to changes in testing rates in the general population over the intervention period (independent of the intervention). However, in all cities except Durban sex workers did not report any increase in HIV testing through public health facilities, and almost all of the increase in HIV testing was through sex worker-focused services.

A recent systematic review of sex worker interventions in China found that in six studies that evaluated comprehensive interventions involving HIV testing, there was a significant increase in the fraction of female sex workers who reported having tested for HIV in the last 12 months (OR 8.1, 95% CI: 4.0-16.7) [128]. However, it is not clear what proportion of sex workers were reached by these interventions, which makes it difficult to parameterize our model based on these results.

A gamma distribution is assigned to represent the uncertainty around the annual rate of uptake of HIV testing through FSW-focused programmes. Based on the results of the studies in Côte d'Ivoire and Zimbabwe, we assign a mean of 1.35, the average of the five rates estimated in the three studies. A standard deviation of 1 is assigned; the resulting 2.5 and 97.5 percentiles of the prior distribution are 0.14 and 3.90 respectively, thus including the range of estimates presented.

2.3.7 HIV testing targeted to adolescents/school-based testing

Although there have been a few HIV testing interventions piloted in South African schools by NGOs [129, 130], no national policy on HIV testing in schools has been adopted. In a recent survey of South African high school students in two provinces, 72% of students reported that they would get tested at school if HIV testing was offered in schools [131]. Acceptability was significantly higher in girls (76%) than in boys (67%). However, actual uptake was much lower (18%) when HCT was offered at schools in northern KwaZulu-Natal as part of an intervention that involved the use of drama to promote HIV testing [130]. A recent household survey in the Western Cape and Mpumalanga provinces found that of the 21.7% of adolescents who reported having ever tested for HIV, 71.8% reported having tested for HIV as part of a school-based

campaign; this suggests a relatively high rate of uptake of testing in schools, considering that only 23% of all youth reported having ever been exposed to school testing programmes [132]. As noted previously, this study also found that there was a strong association between having recently tested for HIV and being sexually experienced (aOR 4.7, 95% CI: 3.1-7.3) (Franziska Meinck, personal communication).

In our model we assume that the rate of uptake of HIV testing in sexually experienced high school students is 67% and 76% in boys and girls respectively, based on the previously-cited survey of acceptability levels [131], and based on the high proportions tested through school-based testing programmes in a recent household survey [132]. However, lower rates of HIV testing are assumed to apply in youth who are not sexually experienced. To represent the uncertainty regarding the effect of virginity on HIV testing uptake, we assign a gamma prior with a mean of 0.5 and a standard deviation of 0.2. This distribution has 2.5 and 97.5 percentiles of 0.13 and 0.87 respectively; the upper limit is close to 1 (i.e. implying little difference between virgins and sexually-experienced adolescents), while the lower limit corresponds to the lower limit estimated from the work of Meinck *et al* (1/7.3). HIV testing is assumed to be offered once every two years on average, i.e. at a testing frequency similar to that assumed for home-based testing.

MicroCOSM simulates educational attainment and grade progression by assigning to each individual in the population a highest grade passed and a variable indicating whether they are currently in school/studying. Age-specific rates of entry into grade 1 are assumed, and for each grade, annual probabilities of school-dropout and grade repetition are specified, which vary in relation to sex and race. Adolescent girls and young women can also drop out of school as a result of pregnancy. The model has been calibrated to various South African education statistics, and a more detailed description of the model of educational attainment is provided elsewhere [10]. For the purpose of the current analysis, it is assumed that HIV testing is offered only to those adolescents and youth who are currently in high school, i.e. currently in grades 8-12.

2.3.8 HIV testing in workplaces

A number of studies have evaluated the potential impact of HIV testing in workforce populations, which may be particularly important in reaching men. In a study of a company-based testing intervention in Zimbabwe, Corbett *et al* [133] found that 49.5% of company employees received HIV testing; rates of testing were similar in men and women. A multinational brewing company conducted more routine HIV testing among employees in five African countries and found that the annual fraction of eligible employees who were tested for HIV was higher in women (28%) than in men (22%), although the vast majority of the workforce was male [134]. Another study conducted among health workers in seven Kenyan hospitals found that the uptake of self-testing was substantially higher among women (37%) than among men (29%) [135].

Table S2.6 summarizes the results of a logistic regression model applied to data from the 2015 South African Labour Force Survey (Quarter 3), to assess factors affecting the probability of employment (defined in the survey as having done any paid work in the last week). The results confirm that employment rates are substantially higher in men than in women. These results are used to predict the probability of employment, for each individual in the model, at the start of each year (individuals who are currently in school/studying and individuals who are younger

than 15 are assumed not to be employed). This is a relatively simple model, as it does not take into account that employment status in year *t* is likely to be a function of employment status in year *t*-1 (although the dependence of the two probabilities on the demographic variables shown in Table S2.6 does nevertheless ensure that there is strong correlation). Further work is required to model changes in employment status more realistically.

Table S2.6: Factors associated with employment in South Africa (2015)

Variable		Adjusted odds ratio (95% CI)
Sex	Male	1.00
	Female	0.61 (0.58-0.64)
Location	Rural	1.0
	Urban	1.51 (1.43-1.59)
Age	15-19	1.00
	20-24	2.54 (2.01-3.21)
	25-29	4.56 (3.62-5.73)
	30-34	5.88 (4.67-7.39)
	35-39	6.61 (5.24-8.32)
	40-44	7.11 (5.64-8.96)
	45-49	7.00 (5.54-8.85)
	50-54	5.34 (4.22-6.77)
	55-59	4.24 (3.33-5.38)
	60-64	1.52 (1.18-1.96)
	65+	0.24 (0.18-0.33)
Education (highest grade passed)	None	1.00
	1	1.16 (0.61-2.21)
	2	0.97 (0.53-1.79)
	3	0.86 (0.47-1.57)
	4	0.82 (0.45-1.47)
	5	0.93 (0.52-1.68)
	6	1.11 (0.62-1.97)
	7	1.10 (0.62-1.95)
	8	0.95 (0.54-1.69)
	9	1.05 (0.59-1.85)
	10	1.23 (0.70-2.16)
	11	1.17 (0.67-2.07)
	12	1.97 (1.12-3.47)
	Tertiary	3.47 (1.95-6.16)
	Other	1.16 (0.65-2.04)
Race	Black	1.00
	Coloured*	1.42 (1.30-1.54)
	Asian	0.79 (0.68-0.92)
	White	1.23 (1.11-1.37)
Constant		0.106 (0.067-0.230)

Source: South African Labour Force Survey 2015, Quarter 3 (authors' own calculations). * This term is used in South Africa to refer to individuals of mixed race.

To model the impact of workplace testing programmes on the annual rate of HIV testing, we apply an annual rate of HIV test uptake to all employed individuals, with this annual rate being multiplied by the fraction of the workforce population that has access to regular workplace

HIV testing. The rate of test uptake is set to 22% in men and 28% in women, based on the estimates of van der Borght *et al* [134]. (Although this is lower than the level of HIV testing observed by Corbett *et al* [133], the latter study expressed uptake as a cumulative fraction tested rather than as an annual rate, and was a randomized trial over a two-year term, making it less applicable in estimating longer-term annual uptake in routine settings.) The proportion of the workforce that might realistically have access to regular workplace testing is more difficult to estimate. In 2018 it was estimated that 69% of the employed population was working in the formal non-agricultural sector [136], and this might be considered an upper bound on the fraction that could be reached by workplace testing, since self-employed individuals, subsistence farmers and agricultural workers in rural areas would probably not access workplace testing. To represent the uncertainty around the fraction of the employed population that would have access to regular workplace testing, we assign a beta prior with a mean of 30% and a standard deviation of 18%. This distribution has 2.5 and 97.5 percentiles of 3% and 70% respectively, thus representing wide ranges of uncertainty, with the upper bound on the range being the fraction of the employed population in the formal non-agricultural sector.

2.3.9 Testing of male partners of pregnant women

Although partner notification interventions have been described previously, these interventions are applicable only when an individual has been diagnosed positive and there is an interest in making sure that their sexual partner is also tested for HIV. In the context of antenatal care, there is also interest in testing the partners of women who are HIV-negative, recognizing that women have high HIV incidence rates during the antenatal and postpartum periods, and that maternal HIV incidence during this period is associated with a high mother-to-child transmission risk [137, 138]. Table S2.7 summarizes the results of a number of African studies that have invited male partners for antenatal HIV screening. In studies in which men were offered a formal invitation letter, the average proportion of male partners tested was 33%, while in two studies in which women were encouraged verbally to bring their partners on their next antenatal visit (with no formal letter of invitation), the proportion of men who were tested was on average 14%. As noted in section 2.2.4, most of the studies have been conducted in settings in which there is a high rate of marriage or cohabitation. Because there is a strong positive association between marital status and partners seeking antenatal testing [63, 139, 140], the results of these studies may be less generalizable to South Africa, where a high proportion of pregnant women are not married or living with their partners.

Table S2.7: African studies of male antenatal screening

Form of invitation	Study	Location	% of couples married or cohabiting	% of men tested
Invitation letter	Krakowiak *et al* [141]	Kisumu, Kenya	100%	39%
	Osoti *et al* [142]	Nyanza, Kenya	100%	36%
	Jefferys *et al* [140]	Mbeya, Tanzania	74%	43%
	Byamugisha *et al* [143]	Mbale, Uganda	99%	15.5%
	Mohlala *et al* [144]	Cape Town, SA	67%	32%
Verbal offer	Katz *et al* [139]	Nairobi, Kenya	91%	16%
	Msuya *et al* [63]	Moshi, Tanzania	91%	12.5%

It is not clear if the woman's HIV status affects the probability of the male partner getting tested. Mohlala *et al* [144] found that the odds of male partner attendance was significantly

higher if the woman was HIV-positive (aOR 1.50, 95% CI: 1.11-2.02). In contrast, Jefferys *et al* [140] found that in women who self-reported that they were HIV-positive, there was a significantly lower proportion of their male partners who came to the antenatal clinic for HIV testing (when compared to women with a self-reported negative status). This may have been because most of the women who reported they were HIV-positive already knew that their partners were HIV-positive, or it may have been because this study promoted couple-based testing, which may have been less acceptable to HIV-positive women. In the absence of consistent evidence, it is assumed that the fraction of male partners attending the clinic for HIV testing is independent of the woman's HIV status. However, the model does allow implicitly for a lower rate of referral by HIV-positive women, (a) because HIV-positive women are less likely to be in marital relationships (which have higher referral probabilities associated), and (b) because HIV-positive women are more likely to have partners who have already been diagnosed HIV-positive (HIV-diagnosed individuals are assumed less likely to get tested).

Interventions may be effective in increasing the fraction of male partners who receive HIV screening. Two studies in the Nyanza province of Kenya found that home visits to contact male partners significantly increased the fraction of male partners tested (to 87% [141] and 85% [142], corresponding to odds ratios of 10.5 and 10.1 respectively). However, the intervention was limited to couples who were married or cohabiting, and the intervention would probably be less appropriate to other couples.

In our model it is assumed in the baseline scenario that there is no HIV testing of male partners of pregnant women. We consider two possible interventions to encourage partner testing. In the first, we consider the effect of providing self-testing kits to women to give to their male partners (discussed in section 2.3.10). In the second, we assume that providing invitation letters to pregnant women for their partners to get tested would lead to a proportion of partners getting tested, with the proportion depending on the partnership type. To represent the uncertainty around the proportion that applies in the context of marital relationships, we assign a beta prior with a mean of 33% (the average of the results in Table S2.7, in which almost all subjects were married) and a standard deviation of 10%. This distribution has 2.5 and 97.5 percentiles of 15% and 53% respectively (wide enough to include all of the estimates from the studies that included invitation letters in Table S2.7). The odds of a non-marital partner testing is assumed to be 0.25 times the odds of a marital partner testing [63, 139, 140]. For example, if the assumed testing probability in marital partners is 0.33, the assumed testing probability for non-marital partners is 0.11 ((0.11/(1 – 0.11))/(0.33/(1 – 0.33)) = 0.25).

2.3.10 Self-testing

A number of studies have examined the feasibility of self-testing strategies to increase the uptake of testing. In a Kenyan antenatal setting, Masters *et al* [145] compared the effect of encouraging women to refer their partners to the clinic for HIV testing and the effect of providing women with self-testing kits for their partners (a ‘secondary distribution’ strategy for self-testing). Of the latter group, 90.8% reported that their partner got tested, as compared to 51.7% in the former group. Secondary distribution of self-testing is also being explored in high risk groups. For example, Carballo-Diéguez *et al* [146] assessed the feasibility of providing self-testing kits to high-risk MSM for testing their prospective sexual partners in the US (‘point-of-sex testing’). In South Africa, Lippman *et al* [147] have also evaluated the potential impact of secondary distribution of self-testing kits through MSM, though their results

suggest that a high proportion of the self-testing kits were distributed to family and friends (not necessarily other MSM).

Self-testing has also been considered in the context of home-based testing (community-based distribution). In a Malawian study, Choko *et al* [148] assessed the uptake of self-testing when it was offered as part of a home-based testing intervention. Although a high proportion (91.7%) of household members agreed to self-testing, there was no comparison group in this study, and thus it is difficult to know whether this result is better than or poorer than the uptake that might be expected if conventional HIV testing were offered. Individuals in the study could choose to receive only conventional HIV testing, but none chose this option – which suggests that self-testing is more acceptable than conventional testing. However, in a Zambian trial, nested within the PopART study, only 45% of household members given the choice of a self-test or a rapid test chose the self-test [149]. In this study, the uptake of home-based testing was higher in men who were randomized to receive the offer of self-testing than in those who were offered only rapid testing (OR 1.30, 95% CI: 1.07-1.60), but there was no significant difference in uptake when comparing women offered rapid testing or the choice of rapid or self-testing [149].

In a more recent trial conducted in South Africa, young women were randomly assigned to receive an offer of free HIV testing at a nearby clinic (the control group) or to be offered the choice of a self-testing kit or a free HIV test at a nearby clinic (the intervention group) [150]. In the intervention group, 96% chose to receive self-testing kits rather than go for free testing at a clinic. After 3 months, the uptake of any HIV testing was 97% in the intervention arm compared to 48% in the control arm.

Another possible distribution strategy is to sex workers, through peer educators. Two recent trials in Zambia [151] and Uganda [152] both compared the effect of peer educators referring sex workers to testing at a nearby clinic against the effect of peer educators providing women with self-testing kits, or else providing them with vouchers that they could use to obtain self-testing kits. In the Zambian trial, the proportion of sex workers who reported testing for HIV after one month was 88.5% in the referral arm, compared to 94.9% in the secondary distribution arm (OR 2.4) and 84.4% in the coupon arm (OR 0.70). In the Ugandan trial, the proportion of sex workers who reported testing after 1 month was significantly lower in the referral arm (71.5%) than in the secondary distribution arm (95.2%) and the coupon arm (80.4%). Another study in Zimbabwe found that self-testing was acceptable when offered to sex workers attending a dedicated sex worker clinic, although 46% preferred to receive standard rapid testing [153].

Self-testing has also been provided to individuals who are receiving PrEP [154], and to health workers [135], though with no comparison group to assess the effect of self-testing on overall uptake of testing. The STAR project, currently under way in Malawi, Zambia and Zimbabwe, also aims to assess the feasibility of offering self-testing kits to patients attending health facilities and men attending MMC services (facility-based distribution).

The previously-mentioned self-testing strategies all build upon existing HCT strategies, i.e. strategies that were originally developed with the use of rapid HIV tests performed by health workers. In these situations, the impact of self-testing can be modelled by modifying the assumptions about the uptake of HIV testing rather than by introducing new ‘access channels’ into the model. A recent systematic review and meta-analysis assessed the effect of self-testing on the uptake of testing when compared to conventional testing approaches [155]. The pooled

relative risk (RR) for the effect of self-testing was 2.12 (95% CI: 1.51-2.98). However, for the purpose of parameterizing our model, it is more appropriate to work with an odds ratio (OR), since working on the OR scale prevents rates of uptake in excess of 100%. We calculate the pooled odds ratio as 8.17 (95% CI: 6.47-10.31), using a random effects meta-analysis in Stata 13.1. To represent the uncertainty around the effect of offering self-testing on the odds of testing, we assigned a gamma prior with a mean of 8.17 and a standard deviation of 1; this distribution has 2.5 and 97.5 percentiles of 6.33 and 10.24 respectively, similar to the confidence interval around the OR estimated in the meta-analysis.

We consider two possible self-test distribution strategies: to partners of pregnant women and to household members (strategies described previously). For example, in the case of husbands of pregnant women, if it is assumed that 33% would get tested for HIV if issued formal invitation letters via their partners, and if it is assumed that distributing self-testing kits instead of invitation letters increases the odds of testing 8-fold, the modelled probability of the husband self-testing is 80%, or $(1 + (1 - 0.33)/(0.33 \times 8))^{-1}$ (slightly less than the rate of 91% in the randomized controlled trial of Masters *et al* [145]). In the case of home-based testing, if it is assumed that the coverage of conventional rapid testing uptake is 56% in men and 84% in women (the means of the distributions specified in section 2.3.2), these proportions increase to 91% and 98% respectively if testing teams offer the choice of rapid testing or self-testing (with the latter being recommended for absent household members). These are similar to the uptake rates of 92% and 97% observed when community-based distribution of self-testing kits was evaluated in Malawi [148] and South Africa [150] respectively.

There are other self-testing distribution strategies that could be modelled as fundamentally new 'access channels'. Most importantly, the provision or sale of self-testing kits through pharmacies could potentially change the rate of HIV testing substantially. Legislation in South Africa has recently changed, making it possible for pharmacies to sell HIV self-testing kits. However, the cost of these kits is substantial and may be a barrier to individuals purchasing these tests. State subsidization of these testing kits, or the provision of vouchers to allow high-risk individuals to collect free tests from pharmacies might be strategies for overcoming the cost barrier. The effect of offering subsidized self-test kits to pharmacy clients was evaluated in a recent Kenyan study, which found that the kits were frequently purchased by individuals who had come to the pharmacy specifically for HIV testing, but there was very little demand for the kits among individuals coming to the pharmacy for other reasons [156]. Another study in the US assessed the effect of providing vouchers for self-test kits to MSM, which could be redeemed at local pharmacies, but found that only 19% of vouchers were redeemed [157]. In the absence of data to demonstrate a significant increase in demand for HIV testing as a result of such interventions, we have not attempted to model the impact of self-testing kit sales through pharmacies.

2.4 Test sensitivity and specificity

The MicroCOSM model makes an implicit allowance for imperfect test sensitivity by assuming that individuals in the acute phase of HIV infection (the first 3 months of HIV infection) do not test positive, while all HIV-positive individuals who test after acute infection would test positive. Although this is simplistic, a recent review found that rapid test sensitivity was heavily dependent on the fraction of HIV-positive individuals who had recently acquired HIV and were seronegative [158]. In this review, the average rapid test sensitivity was 93.7% when evaluated relative to nucleic acid amplification tests (NAATs), and this sensitivity was substantially

lower in high-income settings (85.2%), in which the proportion of acute infections was high (13.6%), when compared to the sensitivity in low-income settings (97.4%), in which the proportion of acute infections was low (4.7%). In our model, the simulated fraction of HIV-positive individuals testing for HIV who are in the acute phase of infection varies between 4% and 6% over the 2005-2017 period, consistent with the fraction of 4.7% in low-income settings [158]. This implies a sensitivity of 94-96%, again roughly consistent with the published review of sensitivity estimates [158].

The rapid HIV testing algorithm is assumed to have 100% specificity. Although there is a small risk that some HIV-negative individuals have false-positive results on both their initial test and the confirmatory test, such instances are expected to be rare, and evaluations of the rapid testing algorithms in South Africa have consistently found specificities of 99.6-100.0% [159-161].

Self-testing is assumed to have the same sensitivity and specificity as rapid testing. Although there is concern that self-testing may be less sensitive than rapid testing, particularly when performed by unassisted individuals, a recent review found good agreement between unassisted self-testing and health worker-administered testing in most studies [162]. Individuals who obtain a positive result on self-testing are assumed to seek confirmatory testing at a health facility, and thus even if self-test specificity is poor, the ultimate impact is likely to be minimal because of the high specificity of the confirmatory rapid testing algorithm.

2.5 Linkage to care after diagnosis

Similar to the Thembisa model, MicroCOSM simulates ART initiation as occurring either immediately after HIV diagnosis (if the individual is eligible to receive ART) or after a delay. The probability of starting ART immediately after HIV diagnosis is assumed to depend on the setting in which diagnosis occurs. Table S2.8 summarizes the assumed proportions of patients starting ART immediately after diagnosis, post-2016. South African studies have found high rates of ART initiation in pregnant women soon after diagnosis [50], which is likely to be due in part to the integration of ART into antenatal care, and the imperative to reduce the mother-to-child transmission risk by initiating ART as early in pregnancy as possible. High rates of ART initiation are also observed in patients with TB, presumably because patients who are attending health services regularly to receive TB treatment would find it easier to receive ART as well, and because health workers would urge them to start ART. However, rates of ART initiation in TB patients are somewhat lower than observed in pregnant women. Data from the most recent District Health Barometer indicate that 85% of TB patients with HIV were on ART [50], though this is likely to be an over-estimate of the rate of linkage to ART after TB diagnosis, given that the indicator includes patients who were already on ART prior to developing TB (Katherine Hildebrand, personal communication). In a Cape Town study, the fraction of TB patients diagnosed with HIV who received a CD4 count within 6 months after diagnosis was 16.5% lower than among pregnant women diagnosed with HIV [163]. In the same Cape Town study, rates of linkage were lowest in patients who were diagnosed through general VCT services. In a review of sub-Saharan African studies that examined linkages between HIV diagnostic services and ART services, half of studies included were from South Africa [164]. Restricting analysis to those studies conducted in South Africa, the median proportion of patients who received CD4 testing following HIV diagnosis was around 75% and the median proportion of those receiving CD4 testing who collected their test results was around 80%. Of those who were determined to be ART-eligible, the average proportion who started ART was around 67%. This suggests that of all individuals who are newly diagnosed

and ART-eligible, the proportion who actually start ART within a few months of diagnosis was only about 40% (0.75 × 0.80 × 0.67). Although it might be expected that a higher proportion would apply following the introduction of universal ART eligibility (since CD4 testing is no longer a requirement prior to ART initiation), the South African experience has suggested that the delay due to CD4 testing makes little difference to long-term ART enrolment [165], and we have therefore assumed conservatively that there has been no change in the 40% linkage rate among patients diagnosed through general VCT services. We have used the same assumption about the fraction of patients starting ART soon after diagnosis for other testing modalities for which data are lacking. In the case of partner notification and assisted partner notification, we assume that the probability of linkage (if the partner tests positive) is the same as for facility-based testing (i.e. 0.40).

Table S2.8: Assumed proportions of patients initiating ART immediately after a diagnosis

HIV testing modality	Linkage	Source
Antenatal care	0.93	[50]
TB and OI clinics	0.78	[50]
General VCT	0.40	[164]
STI clinics	0.40	[164]
Prisons	0.40	[164]
Men receiving MMC	0.40	[164]
PrEP patients	0.40	[164]
Family planning clinics	0.40	[164]
MSM-friendly clinics	0.40	[164]
Community-based/non-clinic settings		
Home-based testing	0.27	Based on RR* of 0.68 [103, 166-168]
Mobile clinics	0.27	Based on RR* of 0.68 [103, 166-168]
Sex workers	0.27	Based on RR* of 0.68 [103, 166-168]
Workplaces	0.27	Based on RR* of 0.68 [103, 166-168]
Schools	0.27	Based on RR* of 0.68 [103, 166-168]
Self-testing	0.27	Based on RR* of 0.68 [103, 166-168]

* The relative rate (RR) is allowed to vary in the uncertainty analysis.

Community-based models of HIV testing have lower rates of linkage to care when compared to other facility-based HIV testing approaches. For example, a recent study in rural Uganda found that individuals who were diagnosed positive at health facilities were much more likely to initiate ART (85%) than those diagnosed through home-based testing (56%) [103]. Other studies in Uganda found that only 13-26% of individuals diagnosed HIV-positive through home-based testing were actually evaluated for ART eligibility [169, 170], and a study of home-based HIV testing and mobile testing in Swaziland found that the fraction of newly-diagnosed individuals who subsequently received a CD4 count was only 23% (rates were similar for the two testing groups) [100]. Another study in Swaziland found that the cumulative fraction linked to care within 6 months after diagnosis through home-based testing was only 61% of that achieved in men diagnosed through MMC services [168]. A recent randomized trial in Zambian sex workers found that those who were diagnosed through self-testing were less likely to link to care than those who were diagnosed at clinics (RR 0.73) [151]. Another recent study of HIV-diagnosed sex workers in South Africa found that having been diagnosed through mobile services was associated with a significantly reduced probability of being on ART (aRR 0.71, 95% CI: 0.57-0.89) [166]. A low rate of linkage (32%) was also observed in a South African study of patients diagnosed through mobile clinics [171]. However, another

South Africa study found high rates of linkage to care following home-based testing, when HIV-positive individuals were followed up after diagnosis [172], and a recent trial in Lesotho found that high rates of linkage were possible when individuals were provided their first ART supply at home [173]. This suggests that the success of home-based testing in increasing ART initiation may be enhanced with additional modifications to the standard home-based testing model.

In our model, we apply a relative rate adjustment to the 40% linkage assumption for facility-based testing to obtain the linkage assumption for community-based testing. To represent the uncertainty around this relative rate, we assign a beta distribution with a mean of 0.68 and a standard deviation of 0.15. The mean of this distribution is the average of the relative rates of ART initiation in the Ugandan study (0.56/0.85 = 0.66), the Swazi study (0.61), the Zambian sex worker study (0.73) and the South African sex worker study (0.71). The 2.5 and 97.5 percentiles of the distribution are 0.36 and 0.93 respectively, wide enough to reflect substantial uncertainty.

The model also allows for the possibility that individuals who were previously diagnosed positive, and who did not start ART at the time of prior diagnosis, may start ART immediately after retesting. The probability of starting ART immediately after the retest is assumed to be the same as it would have been if they were being diagnosed for the first time, as a South African study found that previously-diagnosed individuals (who had not previously linked to care) had the same odds of linking to care after a retest as individuals who were newly diagnosed [174].

2.6 Cost model

2.6.1 Structure

We based the cost of HIV testing services on a cost model that was purpose-built for this analysis and integrated into MicroCOSM. Additionally, we calculated the impact of increased testing on the cost of the HIV programme overall, including potential increases in ART uptake resulting from increased knowledge of HIV status (see section 2.6.2).

The model combines different approaches to micro-costing, using bottom-up, ingredients-based and top-down approaches when appropriate. Bottom-up costing refers to the calculation of resource use for each activity or ingredient, most often based on the resources used by an actual patient sample, multiplied by the unit price for this component. Ingredient costing refers to the calculation of cost based on a theoretical list of ingredients and their quantities used, again multiplied by the relevant unit prices. In top-down cost allocation, costs are calculated on a per test basis by dividing the total cost by the number of HIV tests conducted.

All testing is assumed to follow a cascade or algorithm consisting of different steps, in keeping with the South African Department of Health’s testing cascade (Figure S2.3). This cascade includes, at the least, pre-test counselling, testing using rapid tests with or without confirmatory tests as appropriate, and post-test counselling. The test cascades in the model are divided into two groups, one for tests conducted in a facility, such as a primary healthcare clinic (PHC), and another for those conducted through a mobile modality. The cost for each step is calculated separately and then combined for a total cost per modality, allowing for patients to decline

proceeding at any stage while still including the costs incurred up to that point. The number of positive diagnoses found in each of the modalities is taken into account when calculating the number of patients which pass through each step.

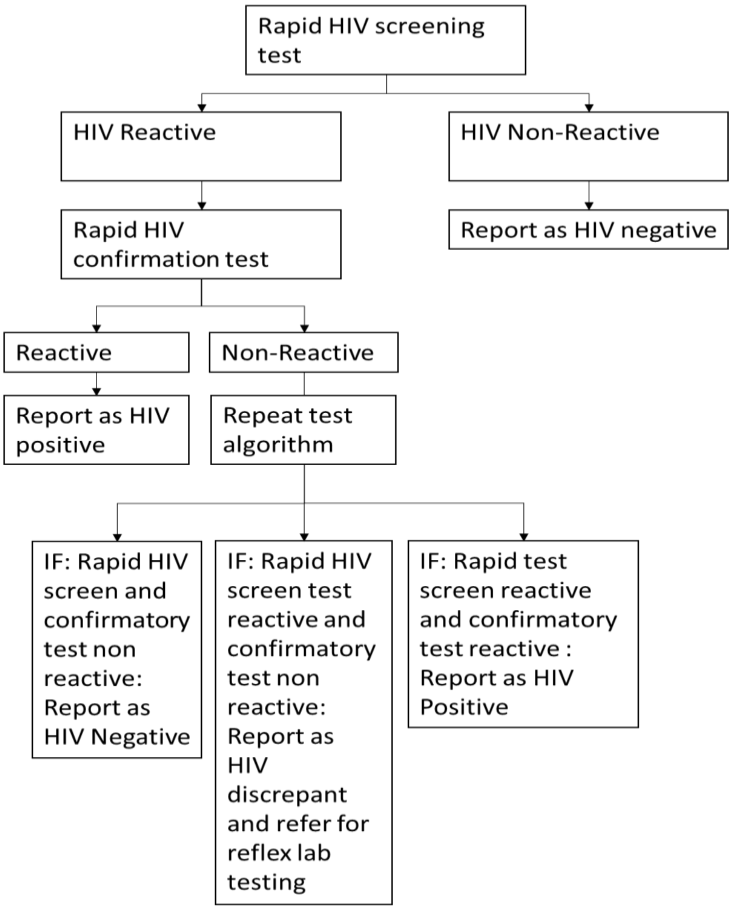

Figure S2.3: National HIV testing algorithm, based on National Department of Health: National HIV Testing Services: Policy 2016

We summarised cost and resource use along the following cost categories: staff, consumable and equipment, overhead, demand creation and targeting costs. Targeting includes all activities that help the service provider identify members of a specific sub-population to be targeted for testing, such as social media adverts to alert men who have sex with men to specific testing opportunities, or contacting companies to arrange workplace testing events. Demand creation on the other hand includes all activities that alert the members of the general population to a testing opportunity, such as flyers, invitations, or community campaigns.

Tests conducted in a facility use a bottom-up approach for the staff, consumable and equipment costs and a top-down approach for the overhead costs, demand creation and targeting. Staff costs are calculated based on the cost per minute of interaction, allowing for only a portion of a staff member’s day to be dedicated to testing. Consumables were costed based on the quantities used during the testing. Overhead costs were allocated as a proportion of the number of HIV tests per month to the total number of patients seen per month. Demand creation and targeting costs were divided across the total number of HIV tests.

Tests conducted through a mobile modality use a top-down approach for staff and equipment, because in a dedicated mobile testing service, staff time and equipment use is not split across other health activities. Overhead costs are also calculated through a top-down approach. Consumable costs are calculated from a bottom-up approach based on the activity.

Resource use was calculated from the perspective of the provider, the public health system. All costs were updated to 2016-17 public-sector prices and salaries and converted to USD using the 07/2016 to 06/2017 period average of 1 USD = 13.58 ZAR [175]. Costs are presented unadjusted for inflation and undiscounted, in order to facilitate the use of total costs results for programme planning and budgeting.

2.6.2 Calculations: Average and total testing cost and total programme cost

Based on the testing cascades, the cost per positive test and the cost per negative test of each modality are calculated. The cost per positive test differs from that of a negative test in two aspects: the need for additional confirmatory rapid tests once the first rapid test is positive, and the longer post-test counselling. The average cost per test is then calculated as a weighted average of the two outcomes based on the number of positive tests out of all tests per modality as calculated by MicroCOSM.

In order to evaluate each scenario's impact on downstream costs in the HIV programme, we furthermore calculated the impact of the testing scenario on the total cost of the South African HIV programme, based on cost inputs for 16 different HIV prevention and treatment interventions included in the South African HIV Investment Case [6]. The number of people receiving each intervention was generated by MicroCOSM, which allowed us to maintain coherence between costs and epidemiological and programme parameters, such as increased testing uptake in specific sub-populations leading to higher uptake of treatment and prevention interventions, and prevention interventions decreasing prevalence and, thus, testing yield.

2.6.3 Calculations: Cost effectiveness

Each modality's incremental cost effectiveness was calculated as two independent metrics: a) incremental cost per HIV infection averted, and b) incremental cost per life year saved. For a), the total cost was compared to the total number of HIV infections averted as calculated by MicroCOSM. For b), we calculated the total number of life years saved in the cohort relative to the West level 26 life table [176]. The cost effectiveness of each intervention scenario was then compared to the cost effectiveness of the base scenario to inform the incremental cost-effectiveness ratios (ICERs).

2.6.4 Cost inputs

The cost of each testing modality was estimated based on a) our own cost analysis (facility-based testing) in Themba Lethu Clinic (TLC) [177]; b) review of published cost estimates with relevance to South Africa [7-9]; and c) interviews with implementers of specific testing modalities (workplace and mobile testing, testing of MSM, FSW and in prisons). The cost of novel interventions such as self-testing was furthermore based on discussions with the HIV Testing Unit in the National Department of Health (NDOH).

Costs were specified per month. Each cost estimate was accompanied by an estimate of testing output, i.e. the number of tests done per month per testing unit (facility or mobile service) by a service set up with the same quantities of staff and other ingredients. For facility-based services, we also added information on the total number of visits (i.e. including those without an HIV test) in order to allocate overhead costs. Wherever possible, we used information on ingredients, cost and coverage from the same source that informed our estimates of testing uptake (Sections 2.2 and 2.3) or, failing that, a source representative of the same type of service.

The exact ingredients, prices and quantities included in the cost of each testing modality is available in Annex 1.

We made a number of adjustments to available cost estimates:

1. When facility-based testing was assumed to occur during a visit for another reason, testing was deemed to be integrated with other services, and the time that staff spent on counselling was reduced by 10 minutes, based on the difference in staff time spent on HCT between our own analysis of the cost of facility-based testing and an estimate of HCT testing integrated into family-planning services based on the study of Sweeney *et al* [178]. This applied to a number of baseline testing modalities (testing as part of STI, antenatal care services, testing as part of PrEP provision to female sex workers, testing as part of MMC, and testing of patients presenting with opportunistic infections).
2. Assuming that population density in urban areas would be higher, resulting in more tests per day through home-based and mobile testing, we adjusted the number of tests done per month taken from Smith *et al* [8] based on a trial in rural KwaZulu-Natal, by a factor of 1.6, the ratio of the proportion of the population living outside a 2 km radius of a clinic between the Formal Urban and Formal Rural categories in the Statistics South Africa Living Conditions Report 2008/9 [179], for home-based and mobile testing in urban areas.

Table S2.9 summarises the sources of and adjustments to cost and output data for each testing modality.

Table S2.9: Sources of cost and test output data for each HCT modality

		Source of service configuration	Source of cost data	Source of output data (number of tests per testing unit per month)
1	General	Themba Lethu Clinic (TLC)	TLC	TLC
2	Antenatal clinics	TLC	TLC[1]	TLC
3	OI patients	TLC	TLC	TLC
4	Prisons	[180] and personal communication, Claudine Hennessey, TB/HIV Care Association		
5	Partners of newly diagnosed	TLC	TLC	TLC
6	Men seeking MMC	TLC; personal communication, Steven Forsythe, Avenir Health	TLC[1]	TLC
7	PrEP	TLC	TLC[1]	TLC
8	STI patients	TLC	TLC[1]	TLC
9	Family-planning clinics	TLC	TLC[1]	TLC
10	Home-based HCT, urban	[7][2]		
11	Home-based HCT, rural	[7]	[7]	[7]
12	Mobile testing, urban	[8][2]		
13	Mobile testing, rural	[8]	[8]	[8]
14	MSM	[122]; TLC + targeting costs based on personal communication, Helen Struthers/James McIntyre, Anova Health Institute		Personal communication, Helen Struthers, Anova Health Institute
15	FSW	Personal communication, Joe Roussow, NACOSA, and Dianne Massawe, Sex Workers Education & Advocacy Taskforce/Red Umbrella programme		
16	Schools	[181]	[8]	[8]
17	Mobile + mobilisation	[6]	[8] + campaign costs from [6]	[8][3]
18	Workplace	[8]; personal communication, Dalene Blom/Jean Slabbert, Foundation for Professional Development		
19	Assisted partner notification	[61]	TLC + demand creation cost based on [9]	TLC
20	ANC partners	[144]	TLC + cost of invitation	TLC
21	Home-based testing + ST offer	Personal communication, Thato Matshaba, NDOH	Same as home-based HCT + cost of ST kits based on personal communication, Jorge Quevedo, Clinton Health Access Initiative South Africa	[8]
22	ANC partners + ST offer	Personal communication, Thato Matshaba, NDOH	Same as ANC partners + cost of ST kits based on personal communication, Jorge Quevedo, Clinton Health Access Initiative South Africa	TLC

[1] Cost of TLC HCT adjusted for integration of services based on [178]

[2] Adjustments to outputs based on the ratio of the proportion of the population living outside a 2km radius of a clinic between the Formal Urban and Formal Rural categories in the StatsSA Living Conditions Report 2008/9 [179]

[3] Adjustment to output based on difference in total tests between modalities 17 and 12/13

2.6.4.1 Cost of facility-based testing

The cost of facility-based testing was based on a bottom-up costing exercise conducted in 2014 at Themba Lethu HIV clinic (TLC) in central Johannesburg. TLC is a dedicated HIV treatment outpatient clinic based at Helen Joseph Hospital (HJH), a secondary hospital providing medical and surgical services to a large urban population. HJH has 12 inpatient medical wards with a total of 347 beds. TLC is one of the largest HCT and HIV/AIDS treatment clinics in the country, with currently more than 30,000 patients on ART.

We gathered descriptive information about the HCT service and model at TLC HCT site through informal meetings with the HCT staff to determine procedures, clinic flow and standard operating practice. This was confirmed by observation of the procedures on site. We defined the HCT costs to include all resource usage from the time of the patient first presenting for testing until the time the patient left the clinic with their confirmed HIV test result, including the pre-test information session, pre-test counselling, testing and post-test counselling.

For the cost analysis, we collated the number of consumables and supplies used for HCT based on our observations. For the calculation of overhead cost, the total space allocated for HCT was measured, and overhead costs were allocated based on the ratio between HCT space and total clinic space. Resource usage was then translated into costs per patient tested by applying unit costs obtained from the site and their suppliers using standard costing methods. Price data for test kits, short-term equipment and other variable inputs were recorded from existing invoices, the public-sector laboratory price list and the national drug tender price list. Salary levels and other types of personnel costs for employees involved in HCT were collected from publicly available public service salaries and benefits information.

Staff costs were calculated based on observed time per test by HIV status, for which we conducted a time-and-motion study to evaluate of the staff time required to provide the full HCT service from pre-test counselling to post-test counselling for a sample of 200 patients presenting for HCT between January and March 2014. HCT staff members completed time logs for each day with predefined fields and random study IDs for each patient interaction. At the beginning of the day, each staff member was given pre-printed logs which they kept with them for the entirety of the day, reporting the duration and nature of each patient interaction during the day. To determine what percentage of total salary cost should be attributed to activities relating to HCT, the average time per HCT session was used (see Table S2.10).

Table S2.10: Results of time-and-motion study for facility-based HCT

Number of patients	200
Number of positive tests	65
Number of negative tests	131
Number of inconclusive tests	1
Number with missing HIV status	3
Tests conducted by nurses	12
Tests conducted by counsellors	188
Average time per positive test (minutes)	39.94
Average time per negative test (minutes)	21.63
Average time per test (minutes)	27.98

Table S2.11 summarises the methods used in the cost analysis of facility-based HCT services.

Table S2.11: Methods used for estimating cost and resource use for facility-based HCT

Type of cost	Method for estimating cost
Variable costs (resources reported in subjects' medical record)	
Diagnostics, supplies, other services reported in HCT register and/or medical record	Actual costs to the site, based on the most recent invoices.
Clinical staff time for HCT	Estimate amount of time required from each level of professional (doctor, nurse, counsellor, etc.) for standard types of HCT visit at the study site and value at average total compensation rate for each level at study site.
Fixed costs (resources used for clinic operation, not allocated to individual subjects)	
Buildings, vehicles, equipment	Estimate total cost from market prices. Take proportion of depreciation plus maintenance and operating costs using standard step-down costing approach. Divide by total number of HCT performed to estimate a cost per HCT.
Management and administration costs, including staff not providing direct patient care to individual subjects	Estimate salaries and other personnel and non-personnel costs of non-clinical staff (e.g. data clerks, cleaners, managers, etc.) and allocate per HCT as described above.

2.6.4.2 Cost of mobile and home-based testing modalities

The cost of mobile and home-based testing was based on two recent economic evaluations of these modalities in South Africa [7, 8]. In each case we used the operational scenario constructed by the authors of these papers (i.e. scenarios that mimic the routine implementation of the intervention evaluated in a clinical trial, in order to correct for trial-induced resource use such as higher staff numbers and research infrastructure), and updated the cost of inputs such as test kits, mobile vans and salaries to 2016. For mobile testing, we assumed that 100% of staff time would be spent either in transport (including walking to households from a centrally parked van in the home-based testing modality) or in counselling and testing activities. For home-base testing, we assumed that only 12% of staff time was spent in counselling and testing activities, in keeping with both the operational scenario of the underlying paper [7] which assumed testing through ward-based outreach teams and with current plans by the Department of Health that favour a model of a mobile "PHC on wheels" that allows healthcare workers to offer a range of PHC services alongside HCT services at the community level.

We assumed a higher number of tests by the same team in an urban as compared to a rural setting, based on personal communication with Dalene Blom from the Foundation for Professional Development, a large-scale implementer of mobile testing in South Africa (see Table S2.9).

2.6.4.3 Cost of mobile testing modalities targeted to key populations

Both MSM and FSW testing were designed as mobile testing activities, in keeping with operational data from agencies currently implementing these services.

For both the MSM and FSW testing modalities we included the cost of targeting testing to these key populations as well as demand creation. In the case of MSM testing, this targeting includes identification of gatekeepers, and staff and consumables costs for outreach teams, support groups, and information and testing events. Since MSM are targeted for other services, not just

HCT, we allocated between 17% and 100% of the total cost of these activities to the testing intervention based on personal communication with Albert Manyuchi from Anova Health Institute.

For FSW testing, we included stipends, travel costs and overheads for peer motivators and project coordinators and the cost of risk reduction workshops.

2.6.4.4 Cost of partner testing modalities

Our analysis includes three different partner testing strategies.
a) Our baseline includes the testing of partners of recently tested individuals. For this, we used the cost of a standard facility-based test, as no additional resources were assumed to be required for the partner to come forward for testing.
Under the new testing modalities we included two partner notification strategies:
b) Unassisted notification of the partners of pregnant women presenting at the ANC. For this, we added the cost of an invitation letter to be handed by the ANC patient to her partner.
c) Assisted notification of the partners of all newly-diagnosed HIV individuals (index cases). We based the cost of this modality on an economic evaluation [9] of a trial that informed our uptake and efficacy assumptions [61]. We assumed that 50% of index cases would opt for contract tracing, in which they would be given a defined time period for their partner to present to the clinic for testing, after which we assumed 45% would be traced by a healthcare workers. The remaining 50% would opt to hand their partner an invitation letter similar to that in b).

2.6.4.5 Cost of community demand creation campaigns

For this modality, we assumed the addition of a demand creation campaign to the mobile testing modality, based the cost of the mobile testing in a rural setting. The campaign costs were based on available data from the South African government's 2016 application to the Global Fund to Fight AIDS, Malaria and Tuberculosis, as reviewed elsewhere [6], and included event and catering costs. We added the cost of covering an entire community with the campaign, though we assumed (for the purpose of calculating the demand creation cost per person tested) that only 60% of people targeted by the campaign come forward for testing, based on data from ANOVA Health Institute.

2.6.4.6 Cost of self-testing

For self-testing we assumed that two populations would access oral self-tests at the target price of \$2.40. This price was based on communication with Jorge Quevedo, CHAI South Africa, and Thato Matshaba, NDOH, and is part of the introduction price list in a tiered pricing structure, so is likely to be reduced further depending on future volumes. We included the cost of a demonstration of the steps involved in self-testing by testing staff, assuming an average of 7 minutes of health worker time for demonstration, per test kit distributed. We also included the cost of confirmatory testing at a facility for those with a positive self-test result, but in keeping with the assumptions in MicroCOSM, we assumed that only 27% of people with a positive self-test would present for confirmatory testing at a facility (Section 2.5). We did not include the costs of telephone calls to respond to user questions, or calls to follow up individuals who received test kits through secondary distribution.

3. Additional results

3.1 HIV testing and diagnosis up to 2016

Figure S3.1 shows the model estimates of the fraction of the HIV-positive adult population that is diagnosed, and compares these with the results from Thembisa, which has previously been fitted to South African HIV testing data [31]. The two models are roughly consistent, although the Thembisa model produces slightly higher levels of HIV diagnosis in the most recent years. This is probably because MicroCOSM simulates more realistically the heterogeneity in HIV testing rates (for example, the relatively low rates of HIV testing in less educated individuals) and hence the challenges in getting HIV testing services to "hard-to-reach" groups. In addition, the Thembisa model estimates a steeper decline in HIV incidence in recent years than MicroCOSM, and since the undiagnosed fraction is strongly dependent on recent HIV incidence, a higher diagnosed fraction would be expected when using the Thembisa model.

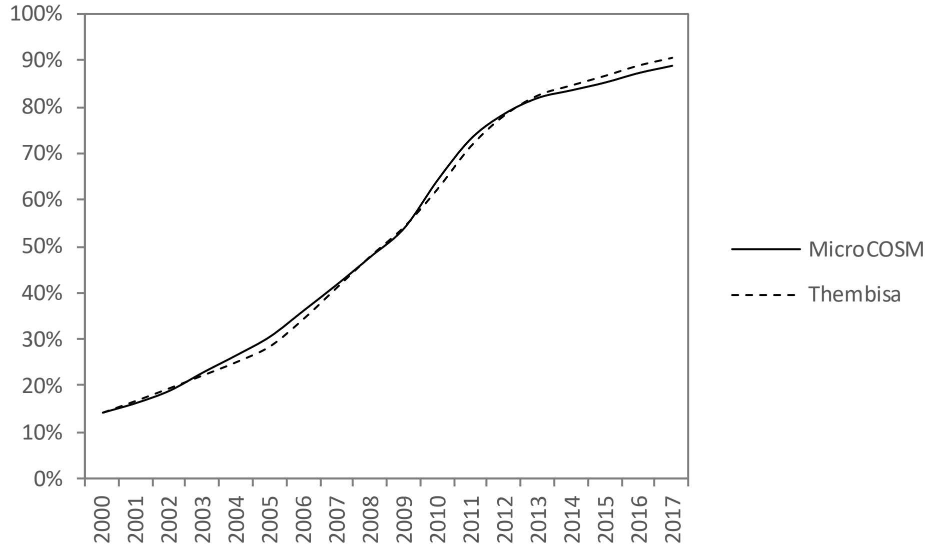

Figure S3.1: Fraction of HIV-positive adults who have been diagnosed positive

Figure S3.2 shows the age and sex differences in the fraction of the HIV-positive adult population that is diagnosed. In the early stages of the HIV epidemic, levels of HIV diagnosis were highest in young HIV-positive adults (aged <50) because of relatively low rates of HIV testing at older ages. However, over time, levels of HIV diagnosis in older adults have increased sharply as a result of the long-term survival of adults on ART. Although levels of HIV diagnosis in HIV-positive youth have also increased, they have not increased as steeply, because a high proportion of incident HIV infections occur among youth, and recently-acquired HIV infections are less likely to have been diagnosed than infections of longer duration. Levels of HIV diagnosis are also substantially lower among men than among women, in part because men are not tested through antenatal services, and in part because lower rates of HIV testing are assumed for men through the 'general' HIV testing modality.

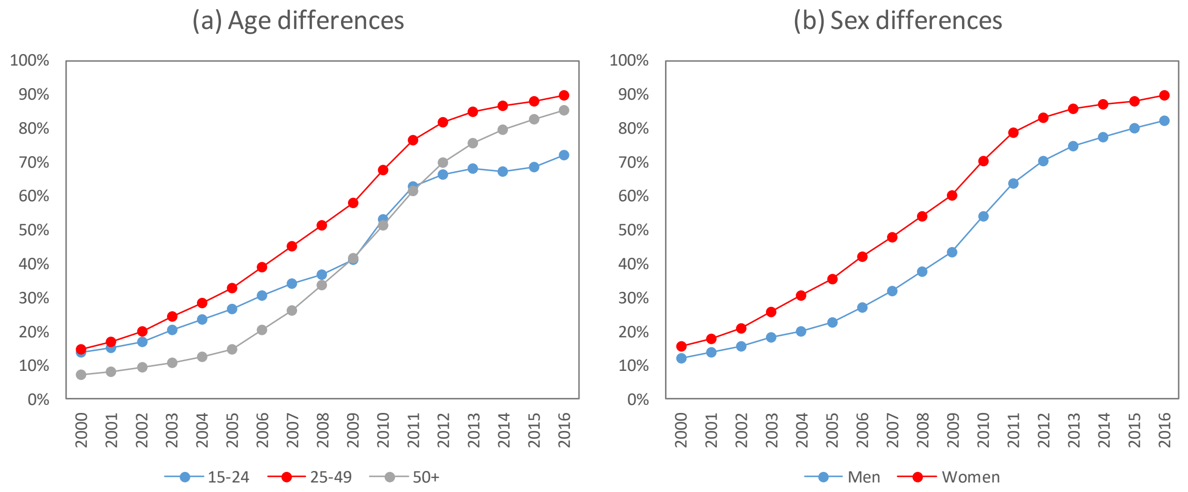

Figure S3.2: Fraction of HIV-positive adults who have been diagnosed positive

Figure S3.3 shows the proportions of individuals tested through each modality and the proportions newly diagnosed through each testing modality, over the period from mid-2010 to mid-2015. The 'general' HIV testing modality is estimated to have accounted for the majority of HIV tests and close to half of all new HIV-positive diagnoses. Patients with symptoms of HIV-related illness are estimated to have accounted for only 9.8% of all HIV tests, but comprise 32.5% of all new HIV diagnoses.

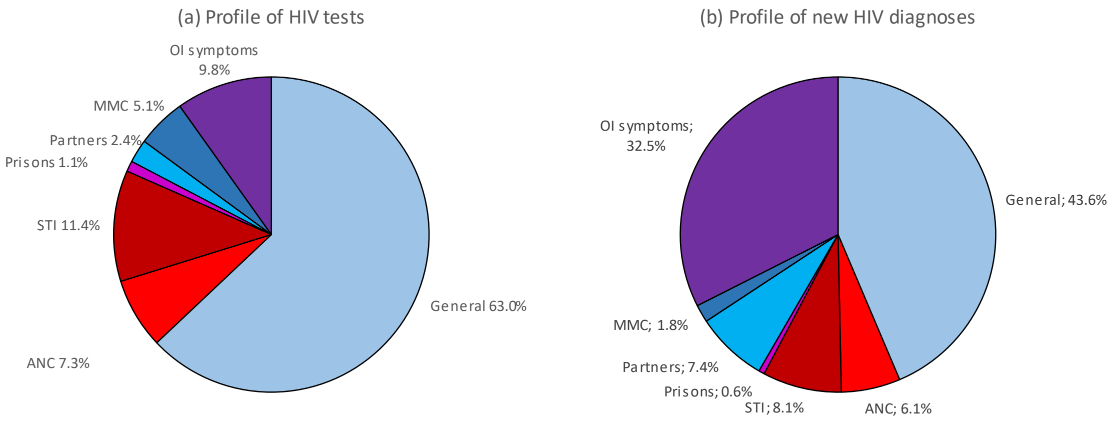

Figure S3.3: Breakdown of total tests and total new diagnoses by testing modality (2010-2015)
ANC = antenatal clinic, MMC = medical male circumcision, OI = opportunistic infections, STI = sexually transmitted infection patients.

These results can also be expressed in terms of the yield on HIV testing, shown in Table S3.1. HIV testing yields were highest in patients with OI symptoms and in partners of newly diagnosed individuals who sought testing as a result of their partner disclosing their HIV status to them. Yields in STI patients are similar to those in general HIV testing services. However, yields on HIV testing in antenatal clinics are lower than in general HIV testing because there is a high rate of repeat testing during pregnancy (i.e. women who test negative at their first antenatal visit are usually counselled to test again later in pregnancy). Yields are lowest in men seeking MMC, because most men who seek MMC are relatively young (close to 40% of all

MMC operations occur in the 10-14 age group [88]) and levels of HIV prevalence are relatively low in these young men. Yields are also relatively low for HIV testing in prisons, for the same reason; more than 40% of prisoners are aged 25 or younger [75, 182, 183], and this is a group with a relatively low HIV prevalence.

Table S3.1: HIV testing yields (new diagnoses per 100 tests) for different HIV testing modalities (2010-2015)

Modality	Yield (95% CI)
General	3.92 (3.89-3.95)
Antenatal clinics	3.19 (3.15-3.22)
STI patients	4.03 (3.98-4.07)
Prisons	3.12 (3.03-3.21)
Partners	17.37 (17.17-17.57)
Men seeking MMC	2.02 (1.98-2.06)
OI patients	18.62 (18.52-18.73)
Total	5.66 (5.62-5.70)

3.2 Future trends in HIV test uptake and diagnosis

Figure S3.4 shows the model projections of the fraction of HIV-positive adults who are expected to remain undiagnosed in 2030, under various HIV testing scenarios. In the absence of any change in policy, the undiagnosed fraction is expected to decline to 6.3% in 2030 (7.7% in HIV-positive men). Home-based testing is the strategy that is expected to have the greatest impact in terms of reducing the undiagnosed fraction, and its impact is expected to be enhanced if self-testing is offered in the context of home-based testing (3.5% undiagnosed in 2030, compared to 3.9% in the absence of a self-testing option). Mobile testing also has the potential to reduce the undiagnosed fraction substantially (to 5.7% in the absence of community mobilization, and to 5.3% when coupled with community mobilization). The promotion of HIV testing in family planning services could also substantially reduce the overall fraction undiagnosed (to 5.6%), although it would have little impact on the rate of HIV diagnosis in men. Workplace HIV testing and secondary distribution of self-testing kits to partners of pregnant women are expected to reduce the fraction of HIV-positive men who are undiagnosed, to 7.4% and 7.5% respectively (Figure S3.4b), but have relatively little impact on overall levels of HIV diagnosis. For all the other new HIV testing strategies considered, the projected impact on the fraction undiagnosed is expected to be relatively modest when compared to the fraction undiagnosed in the base scenario.

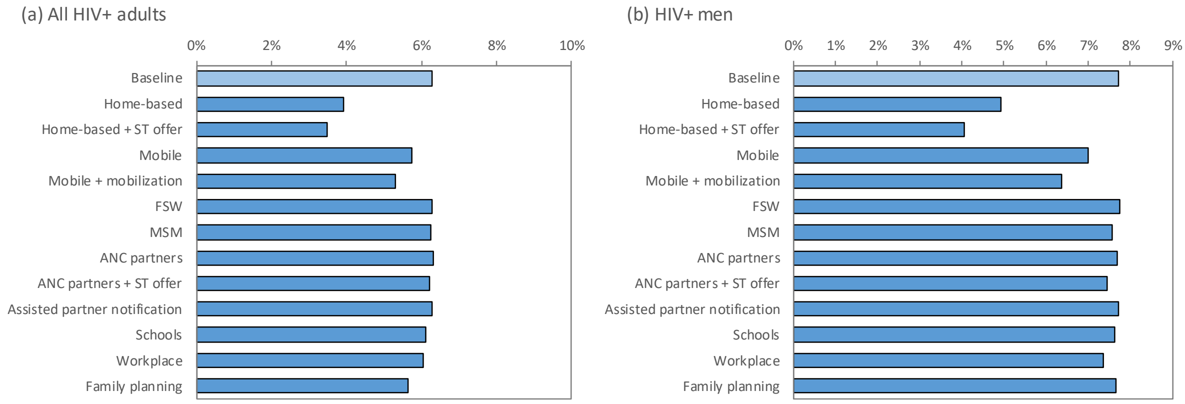

Figure S3.4: Fraction of HIV-positive adults who remain undiagnosed in 2030, under different testing scenarios

Figure S3.5 shows the projected changes in the fraction diagnosed in key populations, with and without special testing interventions targeted to these key populations. The fraction of sex workers who are diagnosed is expected to increase only slightly in the scenario in which dedicated mobile outreach is introduced for sex workers, because there is a high rate of sex worker 'turnover', i.e. women who are diagnosed through the intervention are likely to leave the sex worker population soon after diagnosis. In the absence of intervention, the level of HIV diagnosis is unlikely to increase much above 80%, as this is a relatively young population in which there is a high rate of HIV incidence, and the high HIV incidence rate implies that a relatively low fraction of HIV cases will be diagnosed. The fraction of HIV-positive MSM who are diagnosed is expected to increase substantially if the MSM-targeted intervention is introduced, although probably not enough to reach the 95% target by 2030. MSM have a younger age profile than men in the general population, and this explains why rates of HIV diagnosis are lower among MSM than among men in the general population.

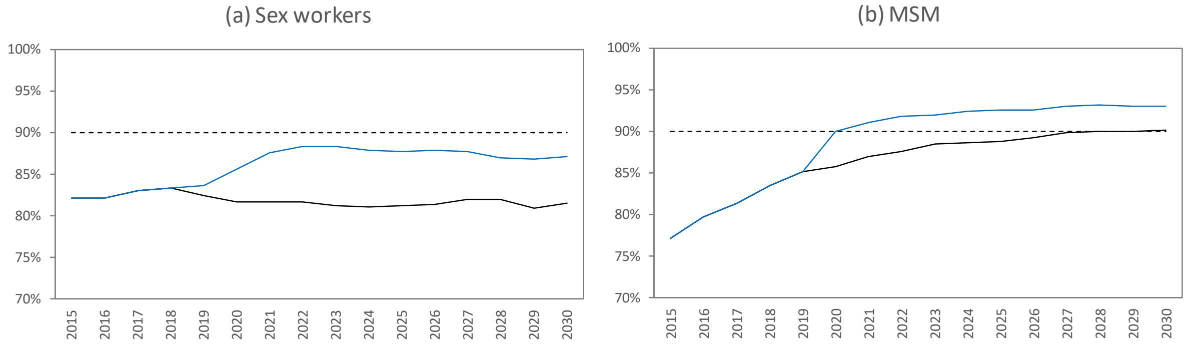

Figure S3.5: Projected future levels of HIV diagnosis in HIV-positive sex workers and MSM
Solid black line represents projections in the absence of any change to current HIV testing policy. Blue line represents projections under the assumption of testing targeted to key populations. Dashed lines represent the 90% target set by UNAIDS.

Figure S3.6 shows the model estimates of the numbers of HIV infections averted in each intervention scenario, over the period from mid-2019 to mid-2039. The testing strategies that have the greatest impact on levels of HIV diagnosis (Figure S3.4) are also the testing strategies that are expected to have the greatest impact on HIV incidence: reductions in new HIV

infections are expected to be greatest in the context of home-based HIV testing, mobile testing and testing in family planning clinics. However, the confidence intervals around these estimates are wide, and it is not possible to conclude that antenatal testing of partners would reduce HIV incidence significantly.

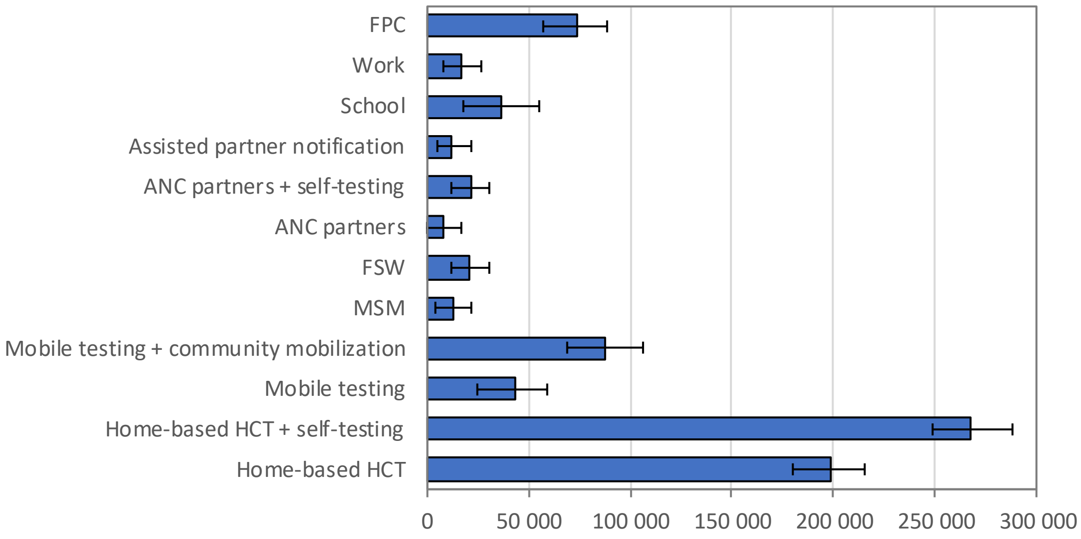

Figure S3.6: Number of HIV infections averted (relative to base intervention scenario), over 2019-2039 period, by HIV testing strategy

A similar ranking of interventions is obtained when considering life years saved as a result of HIV testing (Figure S3.7). Home-based testing, mobile testing and testing in family planning clinics are again the strategies that have the greatest impact, followed by workplace testing and school-based testing.

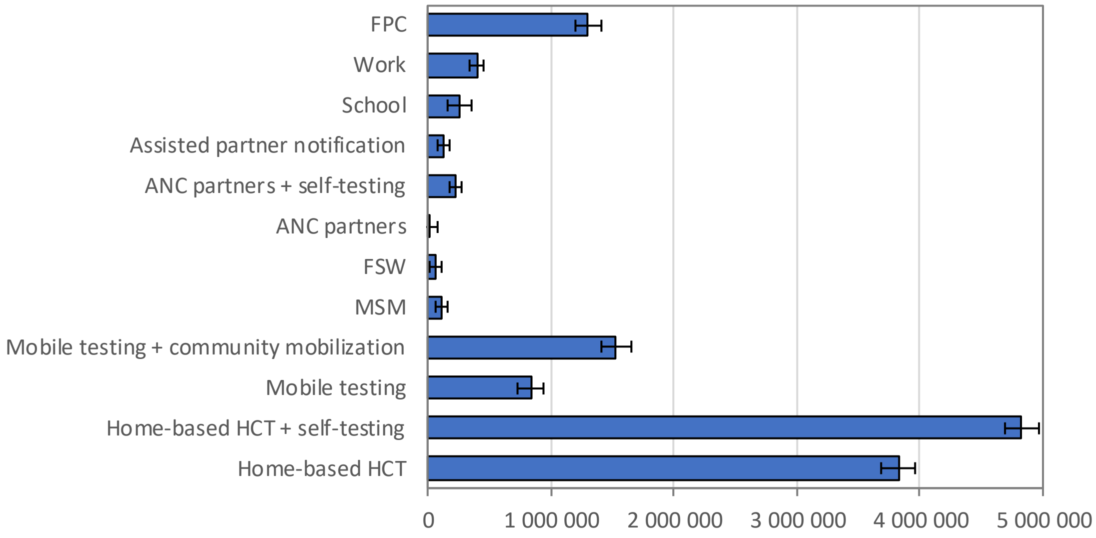

Figure S3.7: Number of life years saved (relative to base intervention scenario), over 2019-2039 period, by HIV testing strategy

3.3 Average and total cost, number of tests and additional diagnoses

The cost per item included in the cost of each testing modality is available in Annex 1.

The cost per test is higher for positive than for negative test results across all modalities due to the need for additional confirmatory tests and the longer post-test counselling (20 minutes instead of 5 minutes) (Table S3.2). Importantly, the costs of most mobile testing modalities are very close to those of facility-based testing modalities, with the exception of mobile testing with community mobilisation. Self-testing has the lowest average cost ($3.08 per test in the case of home-based testing and $3.14 in the case of secondary distribution of self-tests to partners of pregnanct women), as the cost of the test kit is more than offset by the saving in health worker time if the test result is negative (based on our crude cost analysis, excluding the cost of self-test distribution and demonstration). After self-testing, workplace testing is the next cheapest modality, with an average per test cost of $3.64, followed by testing in individuals receiving PrEP and testing in family planning clinics (at $3.97) and testing in men who seek MMC (at $3.99). Mobile testing with a community mobilisation campaign is the most expensive with a cost of $17.46. Partner notification strategies tend to be more expensive, per test performed.

Table S3.2: Average cost by HIV status and modality [2016-17 USD]

	Average cost for a positive test	Average cost for a negative test	Average cost across all tests
Baseline modalities			
Partners of newly diagnosed	6.59	4.73	4.86
Antenatal clinics	5.94	3.97	4.04
OI patients	5.93	3.93	4.40
Men seeking MMC	5.91	3.94	3.99
PrEP	5.92	3.94	3.97
General	6.42	4.57	4.64
STI patients	5.92	3.94	4.09
Prisons	7.50	5.10	5.18
New modalities			
Home-based HCT, urban	4.74	4.17	4.21
Home-based HCT, rural	6.15	5.59	5.62
Mobile testing, urban	5.69	4.16	4.26
Mobile testing, rural	7.63	6.10	6.20
MSM	4.64	4.68	4.67
FSW	6.17	4.64	4.92
Family planning	5.99	3.72	3.97
Assisted partner notification	8.65	6.81	6.95
Schools	7.63	7.07	7.08
Workplace	4.15	3.60	3.64
Home-based testing + ST offer	9.27	2.68	3.08
ANC partners	6.91	5.13	5.18
ANC partners + ST offer	9.25	2.94	3.14
Mobile + mobilization	17.98	17.43	17.46

Starting from an average number of 13.7 million HIV tests per year across the baseline modalities, and based on the uptake and yield results presented in Section 3.2, home-based testing adds the highest number of tests to the HIV programme - about 16 million tests per year (Table S3.3), with 70% of these tests performed in urban areas[1]. Pairing home-based testing with an offer of a self-test increases this number to 21 million tests and results in the highest number of additional tests across all modalities. Mobile testing leads to about 2.5 million additional tests across settings, with about 60% of tests performed in urban areas; this number can be doubled with an additional mobilisation campaign. The lowest number of additional tests is yielded by testing FSW and assisted partner notification.

In terms of the number of newly diagnosed HIV positive people, over 20 years all modalities lead to reductions in HIV incidence, and these reductions in incidence mean that fewer individuals can be diagnosed, with the result that there is a reduction in new diagnoses in some scenarios – and in most scenarios the change in new diagnoses is not significantly different from zero (Table S3.3). The reduction in incidence is due to the combined effect of increased condom use after HIV diagnosis and earlier enrolment in care of HIV-positive clients (i.e. reduced risk of HIV transmission after individuals start ART). This meant that it was not possible to calculate an incremental cost-effectiveness ratio based on incremental cost per additional person found HIV positive, a common metric in economic analyses of HCT interventions. However, over the short term, almost all interventions are expected to lead to increases in new diagnoses. Examples are shown in Figure S3.8 for the home-based testing (with self-test offer) and family planning clinic testing scenarios.

---

[1] Note that we present the results for home-based testing and for mobile testing by locale (urban vs. rural) but also as a combined scenario which is separate from the urban and rural scenarios, so the numbers in the urban and rural scenarios do not add up to the combined scenario.

Table S3.3: Incremental number of tests and number of people found HIV positive by modality

	Incremental tests			Incremental number of people found HIV positive		
	**Median**	**Lower CI**	**Upper CI**	**Median**	**Lower CI**	**Upper CI**
A. Average per year						
Total baseline	13 678 400	13 646 344	13 710 456	292 455	289 492	295 417
New modalities (incremental to baseline)						
Home-based HCT, urban	11 201 238	11 092 050	11 310 426	3 836	2 772	4 899
Home-based HCT, rural	4 767 210	4 720 829	4 813 591	1 196	80	2 312
Home-based HCT (combined)	15 951 888	15 797 802	16 105 974	5 772	4 693	6 850
Mobile testing, urban	1 753 473	1 698 921	1 808 025	645	-397	1 688
Mobile testing, rural	739 628	715 748	763 508	496	-568	1 560
Mobile testing (combined)	2 495 605	2 418 188	2 573 022	1 562	529	2 594
MSM	124 241	115 575	132 906	-447	-1 513	619
FSW	66 079	58 751	73 407	-1 103	-2 231	25
Family planning	2 279 135	2 233 090	2 325 180	389	-663	1 442
Assisted partner notification	38 771	33 668	43 874	484	-434	1 403
Schools	2 918 260	2 869 236	2 967 284	-752	-1 874	369
Workplace	1 004 650	949 901	1 059 399	829	-280	1 938
Home-based testing + ST offer	21 464 300	21 376 171	21 552 429	5 166	4 039	6 292
ANC partners	161 487	153 193	169 781	-52	-1 106	1 001
ANC partners + ST offer	482 438	472 121	492 755	-31	-1 159	1 097
Mobile + mobilization	4 949 550	4 797 385	5 101 715	2 536	1 444	3 628
B. Over 20 years						
Total baseline	273 568 000	272 926 882	274 209 118	5 849 090	5 789 841	5 908 339
New modalities (incremental to baseline)						
Home-based HCT, urban	224 024 760	221 841 006	226 208 514	76 712	55 450	97 975
Home-based HCT, rural	95 344 200	94 416 589	96 271 811	23 922	1 597	46 247
Home-based HCT (combined)	319 037 760	315 956 032	322 119 488	115 435	93 866	137 005
Mobile testing, urban	35 069 460	33 978 418	36 160 502	12 907	-7 950	33 763
Mobile testing, rural	14 792 560	14 314 957	15 270 163	9 916	-11 360	31 191
Mobile testing (combined)	49 912 100	48 363 753	51 460 447	31 233	10 577	51 888
MSM	2 484 810	2 311 497	2 658 123	-8 949	-30 267	12 370
FSW	1 321 580	1 175 010	1 468 150	-22 062	-44 615	492
Family planning	45 582 700	44 661 808	46 503 592	7 783	-13 265	28 831
Assisted partner notification	775 423	673 364	877 482	9 687	-8 681	28 056
Schools	58 365 200	57 384 714	59 345 686	-15 048	-37 482	7 385
Workplace	20 093 000	18 998 011	21 187 989	16 580	-5 593	38 753
Home-based testing + ST offer	429 286 000	427 523 417	431 048 583	103 316	80 782	125 850
ANC partners	3 229 740	3 063 865	3 395 615	-1 048	-22 118	20 023
ANC partners + ST offer	9 648 760	9 442 417	9 855 103	-626	-23 182	21 930
Mobile + mobilization	98 991 000	95 947 708	102 034 292	50 722	28 876	72 567

Uncertainty intervals reflect the standard errors around the outputs of interest, calculated from the 500 scenarios.

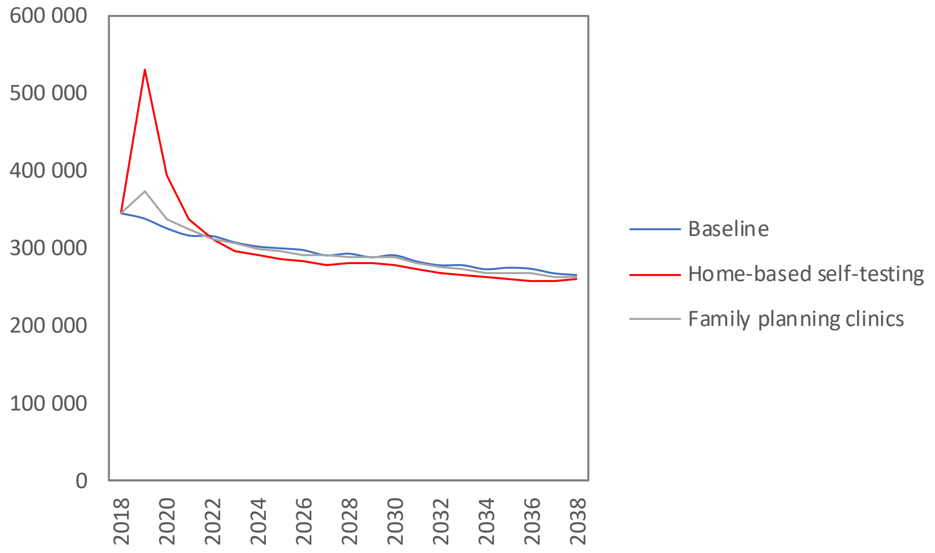

Figure S3.8: Annual new diagnoses in three scenarios

In our base case scenario, the baseline testing modalities cost about $64 million per year on average over the next twenty years (Table S3.4). Each of the new testing modalities will add between $307 000 (FSW testing) and $85 million (mobile testing with community mobilization) per year to this cost, based on the uptake and average cost assumptions of each modality.

Table S3.4: Total and incremental testing cost by modality [2016-17 USD]

	**Total cost**			**Incremental cost**		
	**Base case**	**Lower CI**	**Upper CI**	**Base case**	**Lower CI**	**Upper CI**
A. Average per year						
Total baseline	63 981 326	63 827 368	64 122 320			
New modalities						
Home-based HCT, urban	111 078 532	110 563 155	111 593 909	47 097 790	46 664 966	47 558 773
Home-based HCT, rural	90 632 744	90 329 220	90 985 890	26 649 402	26 423 699	26 945 152
Home-based HCT (combined)	137 694 418	137 055 549	138 333 286	73 713 676	72 934 231	74 442 667
Mobile testing, urban	71 438 910	71 107 451	71 770 369	7 458 169	7 229 333	7 692 983
Mobile testing, rural	68 634 413	68 519 595	68 748 193	4 651 051	4 573 123	4 722 012
Mobile testing (combined)	75 965 674	75 613 212	76 318 136	11 984 933	11 608 766	12 376 791
MSM	64 542 710	64 472 194	64 621 945	561 616	543 114	580 573
FSW	64 287 151	64 216 744	64 365 473	306 709	288 486	324 707
Family planning	72 998 821	72 757 873	73 238 574	9 018 321	8 829 358	9 212 020
Assisted partner notification	64 774 575	64 699 500	64 841 425	792 884	781 349	805 992
Schools	84 153 308	83 785 745	84 562 965	20 163 320	19 847 564	20 513 556
Workplace	67 800 992	67 679 515	67 921 845	3 822 234	3 724 630	3 916 518
Home-based testing + ST offer	130 156 057	129 513 943	130 767 256	66 177 137	65 580 110	66 744 761
ANC partners	64 828 569	64 751 487	64 901 326	846 416	826 708	866 433
ANC partners + ST offer	65 493 824	65 422 918	65 560 150	1 512 016	1 495 631	1 529 568
Mobile + mobilization	148 694 736	146 145 223	151 185 651	84 719 751	82 209 790	87 177 379
B. Over 20 years						
Total baseline	1 279 626 518	1 276 547 359	1 282 446 396			
New modalities						
Home-based HCT, urban	2 221 570 633	2 231 878 174	2 211 263 091	941 955 808	933 299 311	951 175 453
Home-based HCT, rural	1 812 654 886	1 806 584 406	1 819 717 796	532 988 038	528 473 976	538 903 041
Home-based HCT (combined)	2 753 888 354	2 741 110 989	2 766 665 719	1 474 273 529	1 458 684 629	1 488 853 337
Mobile testing, urban	1 428 778 203	1 422 149 023	1 435 407 383	149 163 378	144 586 668	153 859 656
Mobile testing, rural	1 372 688 260	1 370 391 893	1 374 963 851	93 021 016	91 462 458	94 440 232
Mobile testing (combined)	1 519 313 486	1 512 264 244	1 526 362 727	239 698 661	232 175 324	247 535 820
MSM	1 290 854 198	1 289 443 878	1 292 438 899	11 232 316	10 862 273	11 611 460
FSW	1 285 743 017	1 284 334 884	1 287 309 457	6 134 187	5 769 710	6 494 131
Family planning	1 459 976 430	1 455 157 458	1 464 771 485	180 366 423	176 587 156	184 240 400
Assisted partner notification	1 295 491 507	1 293 989 997	1 296 828 510	15 857 671	15 626 973	16 119 838
Schools	1 683 066 163	1 675 714 896	1 691 259 298	403 266 408	396 951 289	410 271 125
Workplace	1 356 019 830	1 353 590 310	1 358 436 909	76 444 687	74 492 597	78 330 365
Home-based testing + ST offer	2 603 121 148	2 590 278 853	2 615 345 119	1 323 542 734	1 311 602 199	1 334 895 211
ANC partners	1 296 571 370	1 295 029 742	1 298 026 527	16 928 326	16 534 169	17 328 655
ANC partners + ST offer	1 309 876 487	1 308 458 350	1 311 203 001	30 240 313	29 912 611	30 591 368
Mobile + mobilization	2 973 894 718	2 922 904 466	3 023 713 022	1 694 395 012	1 644 195 807	1 743 547 576

Uncertainty intervals reflect the standard errors around the outputs of interest, calculated from the 500 scenarios.

Table S3.4 only reports the cost of the testing modality itself. Additionally we evaluated the impact of each testing modality on the cost of the entire government HIV programme, based on cost and uptake data from the South African HIV Investment Case [6] (Table S3.5). In most scenarios, total costs are expected to increase significantly, reflecting both the increased HIV testing costs and the resulting increase in the number of individuals on ART. However, in some

scenarios (for example, testing partners of women attending antenatal clinics), the increase in total HIV costs is less than the increase in testing costs, suggesting a saving in treatment costs due to averted HIV infections, and in some scenarios (testing in FSWs and assisted partner notification) there may even be a reduction in total HIV costs due to the resulting ART cost savings completely offsetting the HIV testing costs. However, the confidence intervals around the cost savings in the latter scenarios are wide enough to include zero.

Table S3.5: Total and incremental HIV programme cost by modality [2016-17 USD]

	**Total cost of HIV programme**	**Incremental cost of HIV programme**	**% change in incremental programme cost** (median, 95% CI)
Total baseline	37 668 700 000		
New modalities			
Home-based HCT, urban	38 946 144 888	1 277 444 888	3.39% (3.27-3.51%)
Home-based HCT, rural	38 318 583 909	658 925 596	1.75% (1.63-1.86%)
Home-based HCT (combined)	39 638 442 989	1 969 742 989	5.23% (5.11-5.36%)
Mobile testing, urban	37 873 108 217	204 408 217	0.54% (0.44-0.65%)
Mobile testing, rural	37 769 899 269	105 989 223	0.28% (0.23-0.34%)
Mobile testing (combined)	37 981 620 854	312 920 854	0.83% (0.72-0.93%)
MSM	37 663 110 750	2 156 105	0.01% (-0.05-0.06%)
FSW	37 656 571 781	-12 128 219	-0.03% (-0.08-0.02%)
Family planning	38 054 524 228	383 234 510	1.02% (0.92-1.13%)
Assisted partner notification	37 658 972 240	-12 971 297	-0.03% (-0.08-0.02%)
Schools	38 063 993 262	393 779 208	1.05% (0.94-1.16%)
Workplace	37 779 400 853	113 202 300	0.3% (0.24-0.35%)
Home-based testing + ST offer	39 572 851 191	1 905 909 328	5.06% (4.94-5.19%)
ANC partners	37 674 502 950	8 036 277	0.02% (-0.03-0.07%)
ANC partners + ST offer	37 697 033 967	29 382 161	0.08% (0.02-0.13%)
Mobile + mobilization	39 532 264 289	1 859 220 089	4.94% (4.75-5.12%)

Uncertainty intervals reflect the standard errors around the outputs of interest, calculated from the 500 scenarios.

3.4 Effectiveness

Starting from a total of about 5.5 million new HIV infections over 20 years, all modalities are expected to reduce the number of new infections, by between 8600 (0.16%, testing of ANC partners) and 268 000 (4.8%, home-based testing with an offer of self-testing) (Table S3.6 and Figure S3.6). The range around this impact straddles zero in the case of testing ANC partners, suggesting that the change over baseline might not be significant.

At baseline we project that a total of 68 million life years will be lost to HIV over 20 years in South Africa (Table S3.6). All modalities reduce this number, although the increase is not significantly different from zero in the antenatal partner testing scenario.

Table S3.6: Infections averted and life years saved by modality

	New HIV infections			Life years lost		
	**Median**	**Lower CI**	**Upper CI**	**Median**	**Lower CI**	**Upper CI**
Total baseline	5 531 610	5 473 463	5 589 757	67 591 000	67 117 840	68 064 160
	**Infections averted**			**Life years saved**		
	**Median**	**Lower CI**	**Upper CI**	**Median**	**Lower CI**	**Upper CI**
New modalities						
Home-based HCT, urban	147 912	128 105	164 132	2 691 009	2 579 361	2 793 876
Home-based HCT, rural	57 320	38 086	75 160	1 143 671	1 032 632	1 258 601
Home-based HCT (combined)	198 398	179 966	215 632	3 826 446	3 690 054	3 960 093
Mobile testing, urban	37 563	17 485	53 802	548 919	442 557	658 698
Mobile testing, rural	10 419	868	18 778	194 132	143 575	250 190
Mobile testing (combined)	43 903	25 203	59 265	842 441	732 180	942 946
MSM	13 120	3 787	22 060	111 187	61 637	162 180
FSW	21 120	12 394	31 058	54 816	5 971	107 368
Family planning	73 702	57 033	89 040	1 304 086	1 198 681	1 405 131
Assisted partner notification	12 410	4 758	21 426	123 329	74 882	168 492
Schools	36 457	18 121	55 586	254 166	157 973	347 935
Workplace	16 948	7 873	26 426	395 833	340 267	448 935
Home-based testing + ST offer	267 701	248 564	288 318	4 829 368	4 695 784	4 975 000
ANC partners	8 582	-935	17 242	20 490	-29 643	72 172
ANC partners + ST offer	21 681	12 456	30 508	230 738	178 349	278 945
Mobile + mobilization	87 224	68 614	106 023	1 532 343	1 415 755	1 653 551

Uncertainty intervals reflect the standard errors around the outputs of interest, calculated from the 500 scenarios.

3.5 Cost effectiveness

Following standard methodology, only modalities with a positive impact on HIV infections averted and/or life years saved were included in the cost-effectiveness analysis. We have excluded the antenatal partner testing scenario from this comparison, as the non-significant savings in life years and HIV infections renders incremental cost-effectiveness ratios (ICERs) difficult to interpret.

When comparing testing cost only with each modality's impact on averting HIV infections, FSW testing appears to be the most cost-effective intervention, followed by MSM testing and assisted partner notification (Table S3.7). The least cost-effective modalities are mobile testing when coupled with community mobilization, school testing and home-based testing campaigns in rural areas. Similar rankings apply when considering HCT costs per life year saved, although the least cost effective is school testing (likely due to the fact that youth who acquire HIV have relatively low mortality rates). However, the ranges around the ICERs are overlapping in many cases, making the selection of the "best buys" difficult.

Table S3.7: Incremental cost effectiveness by modality (testing cost only) [2016-17 USD]

	**Incremental cost per infection averted**			**Incremental cost per life year saved**		
	**Median**	**Lower CI**	**Upper CI**	**Median**	**Lower CI**	**Upper CI**
New modalities						
Home-based HCT, urban	6 372	5 734	7 300	350	337	365
Home-based HCT, rural	9 308	7 079	13 970	466	424	518
Home-based HCT (combined)	7 415	6 846	8 139	385	373	398
Mobile testing, urban	3 971	2 787	8 559	271	229	335
Mobile testing, rural	8 840	4 472	40 885	478	372	648
Mobile testing (combined)	5 467	4 054	9 452	285	253	322
MSM	851	500	2 542	101	69	183
FSW	290	199	484	109	55	504
Family planning	2 450	2 010	3 180	138	128	150
Assisted partner notification	1 276	738	3 327	128	94	211
Schools	11 005	7 286	22 152	1 588	1 161	2 547
Workplace	4 509	2 897	9 661	193	170	223
Home-based testing + ST offer	4 942	4 618	5 296	274	267	282
ANC partners + ST offer	1 398	988	2 425	131	109	169
Mobile + mobilization	19 393	15 999	24 983	1 105	1 029	1 195

Uncertainty intervals reflect the standard errors around the outputs of interest, calculated from the 500 scenarios.

Table S3.8 shows the cost-effectiveness when considering HIV testing costs combined with other HIV programme costs. Some of the testing modalities (particularly FSW testing and assisted partner notification) are predicted to be cost-saving, with the result that ICERs are negative. Other relatively cost-effective testing strategies including testing in MSM, testing in family planning clinics, secondary distribution of self-testing kits to partners of pregnant women, and workplace testing. The least cost-effective strategies are mobile testing when coupled with community mobilization, testing in schools and home-based testing campaigns in rural areas. Mobile testing with community mobilization and school testing both have estimated costs per life year saved of more than the $547-872 threshold identified as fundable within current HIV budget constraints in South Africa [184]. As before, however, confidence intervals around the model estimates are wide, making it difficult to state with confidence which testing strategies represent the best value for money.

Table S3.8: Incremental cost effectiveness by modality (total costs) [2016-17 USD]

	Incremental cost per infection averted			Incremental cost per life year saved		
	**Median**	**Lower CI**	**Upper CI**	**Median**	**Lower CI**	**Upper CI**
New modalities						
Home-based HCT, urban	8 639	7 574	10 177	474	447	508
Home-based HCT, rural	11 480	8 351	17 856	577	492	672
Home-based HCT (combined)	9 926	9 038	11 022	515	491	540
Mobile testing, urban	5 378	3 252	13 957	370	261	537
Mobile testing, rural	10 031	4 236	53 900	549	357	848
Mobile testing (combined)	7 161	4 641	13 239	373	294	470
MSM	160	-1 139	3 493	20	-125	320
FSW	-562	-1 259	584	-233	-785	466
Family planning	5 217	3 876	7 199	294	251	350
Assisted partner notification	-1 046	-2 332	1 219	-105	-226	95
Schools	10 674	6 502	22 781	1 549	1 037	2 686
Workplace	6 668	3 625	15 820	283	203	378
Home-based testing + ST offer	7 118	6 512	7 766	394	379	410
ANC partners + ST offer	1 368	314	3 816	130	34	263
Mobile + mobilization	21 285	17 280	27 675	1 209	1 105	1 340

Uncertainty intervals reflect the standard errors around the outputs of interest, calculated from the 500 scenarios.

3.6 Sensitivity analysis

Table S3.9 shows the effects of the 'baseline parameters' on the cost per life year saved. The 'baseline parameters' are the parameters that were varied in the process of calibrating the model to the South African HIV prevalence data. The table includes the median and inter-quartile ranges of these baseline parameters to illustrate the typical values of these parameters and to facilitate interpretation of the regression parameters. The regression parameters are estimated by regressing the cost per life year saved on all of the parameters shown in the column headings (as well as the intervention parameters, which are presented later). To illustrate, consider the effect of the acute infectivity parameter on the cost per life year saved in the home-based testing scenario. As shown in Table S3.8, the median cost per life year saved in this scenario is $515. Table S3.9 shows that the effect of the acute infectivity parameter in this scenario is -19. This means that when comparing a simulation in which the acute infectivity is 19.3 (the 75th percentile) to a simulation in which the acute infectivity is 16.3 (the median value), we would expect the cost per life year saved to be lower in the former scenario by $57 (i.e. 19 × (19.3 – 16.3)). In other words, if we were to increase the acute infectivity parameter from the median value to its 75th percentile, we would expect to see the cost per life year in the home-based testing scenario reduce from $515 to $458. The parameters that were statistically significant in the regression model are highlighted in bold. Table S3.10 shows a similar analysis of the effects of the different baseline parameters on the cost per HIV infection averted.

Table S3.9: Effects of baseline parameters on incremental cost per life year saved

Parameter	Acute infectivity	Transmission prob. Client-to-FSW	Transmission prob. M-to-F, non-spousal	Transmission prob. F-to-M, non-spousal	RR transmission if spousal (M-to-F)	RR transmission if spousal (F-to-M)
Median	16.3	0.00102	0.00214	0.00107	0.62	0.51
Interquartile range	13.7-19.3	0.00080-0.00125	0.00192-0.00238	0.00093-0.00122	0.39-0.84	0.28-0.76
Effect of parameter on cost per life year saved						
Home-based HCT, urban	**-20 (-29 to -11)**	**-1158 (-2243 to -925)**	**-781 (-1246 to -453)**	**-766 (-882 to -662)**	-78 (-150 to 8)	**-117 (-177 to -37)**
Home-based HCT, rural	**-36 (-60 to -16)**	**-1236 (-2763 to -912)**	-979 (-3136 to 884)	**-939 (-1293 to -767)**	-192 (-334 to 33)	-192 (-333 to 85)
Home-based HCT (combined)	**-19 (-26 to -11)**	**-895 (-1138 to -727)**	-482 (-681 to 229)	**-637 (-717 to -500)**	**-76 (-134 to -13)**	**-136 (-182 to -83)**
Mobile testing, urban	**-82 (-108 to -50)**	**-957 (-1423 to -792)**	-531 (-8813 to 8925)	**-891 (-1550 to -689)**	-202 (-337 to 57)	-219 (-387 to 135)
Mobile testing, rural	**-241 (-276 to -197)**	**-1221 (-1486 to -1060)**	-1224 (-13396 to 16068)	**-1099 (-1323 to -968)**	**-316 (-478 to -61)**	**-601 (-699 to -474)**
Mobile testing (combined)	**-43 (-64 to -25)**	**-1054 (-2820 to -727)**	-160 (-10336 to 7989)	**-941 (-3175 to -590)**	**-201 (-300 to -43)**	-1 (-201 to 385)
MSM	**-105 (-135 to -69)**	**-628 (-834 to -512)**	-174 (-2428 to 1694)	**-506 (-777 to -375)**	-83 (-180 to 77)	**-467 (-1157 to -231)**
FSW	**-60 (-123 to -2)**	**-356 (-661 to -219)**	343 (-1637 to 3452)	**-421 (-2275 to -174)**	**238 (84 to 541)**	**-192 (-472 to -28)**
Family planning	**-35 (-49 to -21)**	**-824 (-1229 to -623)**	-541 (-1109 to 102)	**-687 (-967 to -547)**	-121 (-205 to 18)	-55 (-175 to 127)
Assisted partner notification	**-64 (-93 to -33)**	**-746 (-1752 to -431)**	525 (-1354 to 4101)	**-603 (-1269 to -361)**	**107 (19 to 211)**	**-174 (-417 to -6)**
Schools	**-261 (-423 to -74)**	-2450 (-5203 to 1497)	-1672 (-5472 to 2225)	**-1993 (-2798 to -1688)**	**-1224 (-1449 to -773)**	-645 (-16023 to 6628)
Workplace	**-64 (-85 to -43)**	**-937 (-1182 to -799)**	-101 (-13269 to 18336)	**-725 (-926 to -610)**	**-210 (-294 to -91)**	**-222 (-321 to -58)**
Home-based testing + ST offer	**-9 (-14 to -5)**	**-946 (-1894 to -710)**	**-380 (-529 to -153)**	**-479 (-551 to -382)**	-34 (-74 to 18)	**-73 (-112 to -29)**
ANC partners	**-561 (-619 to -489)**	**-1151 (-1488 to -968)**	-424 (-5973 to 6881)	**-754 (-1007 to -603)**	**-588 (-718 to -415)**	-1064 (-6987 to 1548)
ANC partners + ST offer	**-113 (-135 to -89)**	**-844 (-1111 to -693)**	-100 (-21633 to 11699)	**-572 (-765 to -462)**	14 (-128 to 305)	**-275 (-392 to -119)**
Mobile + mobilization	**-64 (-97 to -31)**	-2267 (-15439 to 5035)	-1772 (-20782 to 11841)	**-1966 (-3629 to -1491)**	-219 (-433 to 106)	**-330 (-523 to -56)**

Values shown are median estimates (with 95% confidence intervals shown in brackets), calculated from bootstrapping the 500 simulations and running a multivariable regression model on each bootstrapped sample. Values formatted in bold are statistically significant. ANC = antenatal clinic attender; FSW = female sex worker; F-to-M = female-to-male; HCT = HIV counselling and testing; MSM = men who have sex with men; M-to-F = male-to-female; M-to-M = male-to-male; RR = relative rate; ST = self-testing.

Table S3.9 (continued): Effects of baseline parameters on incremental cost per life year saved

Parameter	Initial HIV prevalence in high risk women	Sexual mixing	Increased infectivity at CD4 <200 cells/µl	Transmission prob. M-to-M, non-spousal	RR transmission if spousal (M-to-M)
Median	0.0233	0.53	1.52	0.00494	0.51
Interquartile range	0.0175-0.0266	0.34-0.73	1.24-1.95	0.00433-0.00543	0.28-0.76
Effect of parameter on cost per life year saved					
Home-based HCT, urban	-986 (-5087 to 1666)	-22 (-123 to 93)	**-58 (-111 to -1)**	-1045 (-12224 to 8775)	50 (-61 to 201)
Home-based HCT, rural	-1070 (-3586 to 1111)	-133 (-315 to 167)	-89 (-210 to 108)	-1117 (-6062 to 5139)	**641 (111 to 2286)**
Home-based HCT (combined)	-760 (-7473 to 2763)	-64 (-134 to 10)	**-50 (-85 to -10)**	-698 (-6820 to 4928)	75 (-18 to 175)
Mobile testing, urban	-799 (-3105 to 643)	-130 (-327 to 329)	66 (-144 to 426)	-661 (-2551 to 1778)	573 (-17669 to 14317)
Mobile testing, rural	**-952 (-1424 to -752)**	-388 (-576 to 45)	64 (-294 to 2448)	**-953 (-1855 to -621)**	-2361 (-40498 to 27581)
Mobile testing (combined)	**-720 (-1155 to -522)**	-103 (-290 to 263)	-66 (-186 to 127)	-652 (-3212 to 2165)	263 (-83 to 1447)
MSM	**-383 (-494 to -284)**	-177 (-385 to 157)	324 (-123 to 2787)	9 (-8403 to 8490)	-805 (-5697 to 1294)
FSW	**-148 (-355 to -28)**	34 (-104 to 260)	152 (-269 to 1505)	-64 (-261 to 184)	**-228 (-427 to -100)**
Family planning	**-726 (-1344 to -525)**	7 (-136 to 278)	-40 (-116 to 67)	-583 (-5823 to 2361)	230 (-34 to 839)
Assisted partner notification	**-250 (-486 to -128)**	189 (-2756 to 3030)	36 (-1045 to 1284)	-873 (-5426 to 3770)	**-439 (-1045 to -241)**
Schools	**-2008 (-3360 to -1047)**	**-1173 (-1480 to -196)**	-519 (-997 to 1488)	-1881 (-4818 to 2019)	-11 (-17724 to 16413)
Workplace	**-725 (-1045 to -578)**	-208 (-335 to 34)	-104 (-199 to 40)	**-573 (-784 to -361)**	1394 (-24465 to 33990)
Home-based testing + ST offer	-845 (-3862 to 2496)	-27 (-77 to 35)	**-47 (-70 to -21)**	-665 (-3413 to 2625)	24 (-28 to 80)
ANC partners	-399 (-619 to 254)	**-597 (-758 to -376)**	-496 (-3416 to 4049)	-846 (-7970 to 7383)	**-886 (-1227 to -729)**
ANC partners + ST offer	-241 (-469 to 201)	**-343 (-447 to -199)**	181 (-50 to 787)	-696 (-5508 to 3926)	-1746 (-27326 to 15114)
Mobile + mobilization	-1096 (-15358 to 8187)	-254 (-464 to 94)	-75 (-260 to 131)	**-1364 (-2640 to -630)**	238 (-136 to 908)

Values shown are median estimates (with 95% confidence intervals shown in brackets), calculated from bootstrapping the 500 simulations and running a multivariable regression model on each bootstrapped sample. Values formatted in bold are statistically significant. ANC = antenatal clinic attender; FSW = female sex worker; F-to-M = female-to-male; HCT = HIV counselling and testing; MSM = men who have sex with men; M-to-F = male-to-female; M-to-M = male-to-male; RR = relative rate; ST = self-testing.

Table S3.10: Effects of baseline parameters on incremental cost per HIV infection averted

Parameter	Acute infectivity	Transmission prob. Client-to-FSW	Transmission prob. M-to-F, non-spousal	Transmission prob. F-to-M, non-spousal	RR transmission if spousal (M-to-F)	RR transmission if spousal (F-to-M)
Median	16.3	0.00102	0.00214	0.00107	0.62	0.51
Interquartile range	13.7-19.3	0.00080-0.00125	0.00192-0.00238	0.00093-0.00122	0.39-0.84	0.28-0.76
Effect of parameter on cost per life year saved						
Home-based HCT, urban	-295 (-659 to 98)	-14845 (-55754 to 12527)	-12239 (-36186 to 20158)	**-12263 (-18401 to -10925)**	-2097 (-4036 to 2261)	**-3408 (-4720 to -1055)**
Home-based HCT, rural	-954 (-2059 to 268)	**-15629 (-26522 to -5729)**	**-13699 (-26938 to -1939)**	**-14202 (-20768 to -12738)**	-6086 (-8815 to 3468)	-5526 (-8764 to 9941)
Home-based HCT	**-478 (-789 to -140)**	**-14011 (-25683 to -5096)**	-10040 (-39437 to 16254)	**-11497 (-13060 to -9518)**	-1767 (-4091 to 1731)	**-3183 (-4673 to -935)**
Mobile testing, urban	**-1526 (-2089 to -700)**	**-9937 (-20982 to -8327)**	**-7414 (-15436 to -4148)**	-9473 (-30443 to 615)	-3128 (-5551 to 19070)	-3896 (-5695 to 5507)
Mobile testing, rural	**-5652 (-6644 to -4450)**	**-16293 (-20226 to -14662)**	**-13970 (-26104 to -4699)**	**-14276 (-15985 to -13366)**	**-8551 (-9997 to -4688)**	**-11227 (-12244 to -9265)**
Mobile testing	-751 (-1590 to 165)	-14558 (-107929 to 193962)	-9019 (-69387 to 57610)	-13241 (-97011 to 69371)	**-5566 (-6685 to -3233)**	-1973 (-151092 to 256007)
MSM	**-1060 (-1357 to -722)**	**-6156 (-12400 to -4507)**	-622 (-4546 to 6534)	**-5646 (-14750 to -3486)**	-608 (-1346 to 582)	**-2474 (-4691 to -1245)**
FSW	**-445 (-596 to -259)**	**-4889 (-13450 to -3046)**	182 (-42534 to 30488)	**-2938 (-12052 to -1540)**	608 (-133 to 2008)	**-1604 (-2678 to -767)**
Family planning	**-635 (-983 to -161)**	**-10146 (-18616 to -8028)**	-6915 (-11231 to 1949)	**-8207 (-11125 to -7248)**	-2241 (-4017 to 3198)	-1884 (-3830 to 4160)
Assisted partner notification	**-1132 (-1690 to -755)**	-10363 (-239624 to 96628)	3600 (-42577 to 35108)	-7631 (-67935 to 31886)	**1062 (248 to 2791)**	-2323 (-21094 to 10679)
Schools	**-2074 (-3231 to -772)**	**-13926 (-17240 to -12762)**	-11943 (-24985 to 11305)	**-12793 (-15616 to -11573)**	**-8215 (-10156 to -1393)**	-6340 (-53657 to 20673)
Workplace	**-1737 (-2583 to -602)**	**-11038 (-13246 to -10004)**	-9712 (-60646 to 22932)	**-11750 (-23300 to -9720)**	**-5925 (-6813 to -3777)**	**-5949 (-7129 to -2540)**
Home-based + ST offer	**-165 (-337 to -7)**	**-10464 (-14994 to -9058)**	**-6949 (-8517 to -2098)**	**-8041 (-8671 to -7280)**	-1114 (-2388 to 583)	**-1817 (-2884 to -337)**
ANC partners	**-1758 (-2190 to -1323)**	**-6087 (-9500 to -4621)**	-502 (-10361 to 14081)	-5238 (-20941 to 20711)	**-2279 (-3454 to -964)**	**-2700 (-4365 to -1490)**
ANC partners + ST offer	**-1188 (-1402 to -945)**	**-5600 (-7349 to -4744)**	-3805 (-10642 to 1127)	**-3803 (-4701 to -3218)**	43 (-1331 to 5067)	**-2658 (-3780 to -1260)**
Mobile + mobilization	-606 (-1987 to 840)	**-27645 (-57646 to -3597)**	-25429 (-101508 to 99576)	-30425 (-116131 to 64784)	-1809 (-10872 to 44931)	**-10461 (-14204 to -1846)**

Values shown are median estimates (with 95% confidence intervals shown in brackets), calculated from bootstrapping the 500 simulations and running a multivariable regression model on each bootstrapped sample. Values formatted in bold are statistically significant. ANC = antenatal clinic attender; FSW = female sex worker; F-to-M = female-to-male; HCT = HIV counselling and testing; MSM = men who have sex with men; M-to-F = male-to-female; M-to-M = male-to-male; RR = relative rate; ST = self-testing.

Table S3.10 (continued): Effects of baseline parameters on incremental cost per HIV infection averted

Parameter	Initial HIV prevalence in high risk women	Sexual mixing	Increased infectivity at CD4 <200 cells/µl	Transmission prob. M-to-M, non-spousal	RR transmission if spousal (M-to-M)
Median	0.0233	0.53	1.52	0.00494	0.51
Interquartile range	0.0175-0.0266	0.34-0.73	1.24-1.95	0.00433-0.00543	0.28-0.76
Effect of parameter on cost per life year saved					
Home-based HCT, urban	**-9963 (-11377 to -7924)**	-2236 (-4331 to 1713)	-25 (-2079 to 4086)	-11634 (-35897 to 23519)	2914 (-2207 to 22103)
Home-based HCT, rural	**-13090 (-15339 to -11232)**	-6356 (-8760 to 2629)	4935 (-77842 to 181820)	-9721 (-73015 to 25119)	-24678 (-475596 to 413525)
Home-based HCT (combined)	-11012 (-32197 to 10095)	-2796 (-4736 to 186)	1614 (-684 to 6201)	-10232 (-30039 to 33797)	1546 (-2019 to 9758)
Mobile testing, urban	**-7171 (-10090 to -5161)**	**-3840 (-5213 to -15)**	-16225 (-219134 to 133423)	-6782 (-76309 to 22462)	-791 (-203337 to 92634)
Mobile testing, rural	**-12034 (-12911 to -11209)**	**-9731 (-10729 to -7857)**	**-15089 (-29557 to -13001)**	**-13177 (-19263 to -8601)**	-20515 (-156337 to 149482)
Mobile testing (combined)	**-8956 (-10285 to -8035)**	-2641 (-75365 to 84703)	3215 (-260627 to 305393)	-9198 (-28834 to 3835)	2107 (-143359 to 166776)
MSM	**-1909 (-2498 to -1409)**	-837 (-1642 to 419)	**-1491 (-2728 to -339)**	-3483 (-42743 to 42440)	-4273 (-19796 to 5485)
FSW	**-1591 (-2689 to -783)**	-227 (-744 to 660)	886 (-9043 to 16191)	-2032 (-6159 to 3127)	**-2937 (-7279 to -1773)**
Family planning	**-7134 (-8638 to -6094)**	-1414 (-3659 to 5353)	1093 (-1786 to 12467)	-7095 (-41616 to 16272)	3352 (-319627 to 161681)
Assisted partner notification	-398 (-1128 to 160)	**1345 (164 to 4862)**	**1227 (119 to 2926)**	-6377 (-154044 to 118479)	-6147 (-124840 to 68693)
Schools	**-12417 (-14106 to -10820)**	**-8911 (-10328 to -4425)**	-1215 (-17560 to 46032)	-12402 (-29198 to 13923)	-2500 (-83201 to 87336)
Workplace	**-8373 (-9078 to -7738)**	**-6217 (-7056 to -4603)**	-12941 (-76299 to 42506)	-10301 (-33324 to 15361)	**-12481 (-27575 to -10257)**
Home-based testing + ST offer	**-8556 (-10687 to -5308)**	-1146 (-2531 to 883)	79 (-1024 to 1464)	-8702 (-40918 to 31780)	2064 (-231 to 7863)
ANC partners	-821 (-1872 to 995)	**-1888 (-2763 to -681)**	-1014 (-1854 to 606)	-2951 (-14021 to 13393)	-5832 (-27956 to 16092)
ANC partners + ST offer	-2080 (-3583 to 630)	**-2203 (-2699 to -1612)**	-6364 (-61596 to 32835)	-4522 (-23283 to 15642)	**-5947 (-14819 to -4227)**
Mobile + mobilization	**-22715 (-26648 to -20201)**	-9013 (-13462 to 6140)	20678 (-3919 to 226868)	**-22170 (-27949 to -17013)**	13887 (-333633 to 254036)

Values shown are median estimates (with 95% confidence intervals shown in brackets), calculated from bootstrapping the 500 simulations and running a multivariable regression model on each bootstrapped sample. Values formatted in bold are statistically significant. ANC = antenatal clinic attender; FSW = female sex worker; F-to-M = female-to-male; HCT = HIV counselling and testing; MSM = men who have sex with men; M-to-F = male-to-female; M-to-M = male-to-male; RR = relative rate; ST = self-testing.

Almost all of the statistically significant effects shown in Tables S3.9 and S3.10 are negative effects. This is because almost all of the baseline parameters are positively associated with the projected HIV incidence over the period 2019-2039 period, and thus higher parameter values are associated with a greater potential reduction (in absolute terms) in numbers of new HIV infections and hence a greater potential number of life years saved. The greater impact of the intervention (in absolute terms) in turn implies a lower cost per HIV infection averted (or per life year saved), hence the negative relationship. There are, however, some exceptions to the general finding of negative associations. In the assisted partner notification scenario, there are some parameters that are significantly positively associated with the cost per infection averted or the cost per life year saved. As it is assumed that partner referral is more common in the context of cohabiting or marital relationships, the partners referred for testing tend to be older. The age pattern of HIV incidence changes as HIV incidence rates change, with the fraction of incident HIV occurring in older adults being lowest when HIV incidence is highest. This in turn implies that interventions that tend to reach older individuals may be relatively less cost-effective as HIV incidence increases.

Table S3.11 shows the effects of the parameters related to the new testing modalities on the cost per life year saved. The regression coefficients are interpreted in the same way as before. For example, consider the effect of the relative rate of retesting in previously-diagnosed, ART-naïve individuals, on the cost per life year saved in the home-based testing scenario. If the relative rate of retesting were to increase from 0.5 to 0.7, this would imply a reduction in the cost per life year saved of \$29 (-144 × (0.7 – 0.5)) relative to the original estimate of \$515 (Table S3.8). Table S3.12 shows corresponding effects of parameters on the cost per HIV infection averted. As noted in the main text, the relative rate of retesting in previously-diagnosed ART-naïve individuals is generally negatively associated with the cost per life year saved and cost per infection averted, which is because repeat testers are assumed to be more likely to start ART than they would have been if they had not repeated testing. However, the cost per life year saved is in some cases significantly positively associated with the relative rate of testing in patients on ART, which is because there is assumed to be no epidemiological effect of testing people who are already on ART (i.e. there is only a cost with no benefit). Relative rates of linkage to ART when diagnosis occurs through community-based testing modalities are also generally negatively related to the cost per life year saved and cost per HIV infection averted, i.e. relatively more life years are saved when more individuals link to ART after diagnosis, thus reducing the average cost per diagnosis.

Table S3.11: Effects of new HIV testing parameters on incremental cost per life year saved

Parameter	RR testing in previously-diagnosed, ART-naïve	RR testing in ART patients	RR linkage to ART from community-based testing
Home-based HCT, urban	**-78 (-134 to -8)**	**286 (57 to 674)**	-100 (-191 to 12)
Home-based HCT, rural	-126 (-262 to 50)	191 (-190 to 1418)	-13 (-284 to 693)
Home-based HCT (combined)	**-144 (-181 to -103)**	103 (-18 to 263)	**-128 (-194 to -48)**
Mobile testing, urban	193 (-138 to 1305)	-19 (-459 to 2200)	-97 (-383 to 1018)
Mobile testing, rural	21 (-352 to 955)	-311 (-581 to 747)	304 (-23791 to 24183)
Mobile testing (combined)	9 (-162 to 327)	647 (-9373 to 9466)	-20 (-297 to 884)
MSM	-134 (-252 to 17)	-123 (-8162 to 5106)	N/A
FSW	-30 (-324 to 317)	-22 (-4822 to 4504)	-41 (-3528 to 2755)
Family planning	-23 (-132 to 95)	**494 (52 to 2348)**	N/A
Assisted partner notification	40 (-695 to 1412)	18 (-3616 to 6685)	-221 (-5533 to 4178)
Schools	-1064 (-1381 to 25)	-3371 (-19720 to 10149)	-2826 (-32548 to 12818)
Workplace	**-222 (-297 to -111)**	44 (-230 to 1347)	288 (-112 to 1841)
Home-based testing + ST offer	**-78 (-105 to -47)**	**161 (51 to 290)**	**-75 (-133 to -15)**
ANC partners	**-632 (-847 to -334)**	-802 (-13567 to 7660)	-919 (-6193 to 3897)
ANC partners + ST offer	-170 (-358 to 309)	105 (-285 to 3000)	-906 (-10937 to 14493)
Mobile + mobilization	**-422 (-566 to -229)**	363 (-185 to 1988)	-383 (-655 to 86)

Values shown are median estimates (with 95% confidence intervals shown in brackets), calculated from bootstrapping the 500 simulations and running a multivariable regression model on each bootstrapped sample. Values formatted in bold are statistically significant. ANC = antenatal clinic attender; ART = antiretroviral treatment; FSW = female sex worker; HCT = HIV counselling and testing; MSM = men who have sex with men; RR = relative rate; ST = self-testing.

Table S3.12: Effects of new HIV testing parameters on incremental cost per HIV infection averted

Parameter	RR testing in previously-diagnosed, ART-naïve	RR testing in ART patients	RR linkage to ART from community-based testing
Home-based HCT, urban	1044 (-1592 to 7693)	15706 (-291305 to 512959)	-569 (-4031 to 11313)
Home-based HCT, rural	1663 (-45907 to 70186)	-1313 (-149119 to 147372)	-1028 (-134864 to 130227)
Home-based HCT (combined)	**-2205 (-3551 to -230)**	3991 (-1996 to 32775)	-1865 (-4461 to 4531)
Mobile testing, urban	-13842 (-187763 to 94992)	-4249 (-47378 to 78548)	-8246 (-63372 to 51222)
Mobile testing, rural	-3980 (-85314 to 214567)	-8405 (-40438 to 32581)	-17740 (-246256 to 96455)
Mobile testing (combined)	2043 (-157272 to 175102)	-13199 (-144402 to 129137)	-3126 (-106547 to 86301)
MSM	-2773 (-18839 to 13254)	-871 (-30974 to 40257)	N/A
FSW	-1370 (-8851 to 1813)	-396 (-19020 to 24580)	138 (-20870 to 28665)
Family planning	864 (-2628 to 14668)	3204 (-320493 to 151312)	N/A
Assisted partner notification	302 (-10194 to 13782)	-5 (-14553 to 22450)	-581 (-30151 to 19498)
Schools	-6175 (-34121 to 25851)	-21819 (-200075 to 97380)	-17664 (-100941 to 78698)
Workplace	**-5587 (-6903 to -608)**	-3340 (-56294 to 70209)	-1779 (-228630 to 180736)
Home-based testing + ST offer	-1170 (-2280 to 172)	3271 (-863 to 11497)	-1480 (-3368 to 1394)
ANC partners	**-2391 (-4934 to -274)**	-3345 (-37249 to 36873)	-2985 (-74718 to 59489)
ANC partners + ST offer	-1603 (-2737 to 1990)	-1267 (-53334 to 56491)	-5206 (-39773 to 25296)
Mobile + mobilization	-7964 (-225147 to 274047)	1247 (-173416 to 284281)	-3126 (-106547 to 86301)

Values shown are median estimates (with 95% confidence intervals shown in brackets), calculated from bootstrapping the 500 simulations and running a multivariable regression model on each bootstrapped sample. Values formatted in bold are statistically significant. ANC = antenatal clinic attender; ART = antiretroviral treatment; FSW = female sex worker; HCT = HIV counselling and testing; MSM = men who have sex with men; RR = relative rate; ST = self-testing.

Table S3.13 shows the effect of intervention-specific parameters on the cost per life year saved and cost per HIV infection averted in the scenarios that they relate to. The majority of these associations are negative, although only two are significantly negative and one (for FSW testing) is significantly positive. All of these parameters determine the extent of test uptake

under the new testing modalities, and thus the findings of negative associations with the ICERs would suggest *increasing* returns to scale for new HIV testing strategies. This may seem paradoxical, since one might expect diminishing marginal returns from testing as increasingly high levels of testing coverage are achieved. However, infectious disease dynamics are highly non-linear; for example, increasing vaccine coverage from 60% to 70% might be expected to have more impact on incidence (in absolute terms) than increasing vaccine coverage from 50% to 60%, because there is greater herd immunity implied in the former scenario [185]. Although there may well be a degree of 'saturation' in terms of knowledge of HIV status as testing volumes increase, this does not necessarily imply saturation of epidemiological impacts if retesting previously-diagnosed individuals increases their chance of starting ART. The exception is HIV testing in sex workers, where saturation effects do become important at very high rates of testing.

Table S3.13: Effects of intervention-specific parameters on cost-effectiveness ratios

Parameter	Scenario	Effect of parameter: Incremental cost per life year saved	Effect of parameter: Incremental cost per infection averted
Fraction of population tested in each round of home-based testing	Home-based HCT, urban	-48 (-188 to 159)	-3207 (-5742 to 2014)
	Home-based HCT, rural	-10 (-523 to 3897)	-7210 (-103647 to 72436)
	Home-based HCT	-6 (-135 to 160)	-1940 (-5394 to 7121)
	Home-based testing + ST	-107 (-226 to 66)	**-4096 (-5664 to -507)**
Annual rate of testing through mobile testing	Mobile testing, urban	-161 (-311 to 59)	-3149 (-4948 to 2258)
	Mobile testing, rural	-724 (-1486 to 380)	**-11745 (-18349 to -5438)**
	Mobile testing	-33 (-4135 to 3712)	-6175 (-89091 to 53306)
	Mobile + mobilization	21 (-632 to 2454)	-7964 (-225147 to 274047)
Increase in annual testing rate in MSM	MSM	66 (-166 to 993)	523 (-9908 to 13019)
Increase in annual testing rate in FSW	FSW	430 (-1596 to 4312)	**902 (203 to 1989)**
Annual testing rate in family planning clinics	Family planning	-21 (-302 to 1672)	-2658 (-55727 to 77421)
Effect of assisted partner notification on partner referral	Assisted partner notification	102 (-38 to 327)	817 (-2015 to 6976)
RR of school testing in virgins	Schools	-1153 (-14833 to 11534)	-9237 (-92562 to 41522)
Fraction of employed reachable by workplace testing	Workplace	37 (-50 to 158)	4102 (-1902 to 43984)
Fraction of husbands of married pregnant women tested	ANC partners	-91 (-465 to 1933)	1272 (-16092 to 25139)
	ANC partners + ST	33 (-213 to 1418)	-26 (-26697 to 16999)
Effect of self-testing offer on uptake of testing	Home-based testing + ST	-1 (-15 to 14)	-90 (-666 to 524)
	ANC partners + ST	27 (-160 to 421)	-640 (-9526 to 15830)

Values shown are median estimates (with 95% confidence intervals shown in brackets), calculated from bootstrapping the 500 simulations and running a multivariable regression model on each bootstrapped sample. Values formatted in bold are statistically significant. ANC = antenatal clinic attender; FSW = female sex worker; F-to-M = female-to-male; HCT = HIV counselling and testing; MSM = men who have sex with men; M-to-F = male-to-female; M-to-M = male-to-male; RR = relative rate; ST = self-testing.

To better understand the effects of the baseline parameters on the cost per life year saved, we consider a regression specific to the home-based testing with self-testing scenario (since this is the intervention that has the greatest epidemiological impact and thus generates the most statistically significant results). Instead of fitting a single regression model (as in Table S3.9), we consider two regressions: one in which we assess the effect of the baseline HIV parameters on the projected number of new infections over the 2019-39 period (in the absence of any change to current testing policy) and one in which we assess the effect of the number of new infections over the 2019-39 period (in the absence of any change to current testing policy) on

the cost per life year saved (also controlling for the parameters specific to the home-based testing with self-testing scenario). Table S3.14 shows the results of the first regression model. As noted previously, most of the baseline parameters are positively associated with the projected number of new infections over the 2019-39 period. One exception is the initial HIV prevalence in high risk women, which is strongly negatively associated with projected future incidence because a higher initial prevalence implies an epidemic that peaks earlier (and thus declines more over the longer term).

Table S3.14: Effects of baseline parameters on projected numbers of new HIV infections, 2019-2039, in the absence of changes to HIV testing policy

Parameter	Effect (95% CI)
Acute infectivity	56 013 (49 170 to 63 270)
Transmission prob. Client-to-FSW	393 million (322 to 476 million)
Transmission prob. M-to-F, non-spousal	1160 million (1080 to 1270 million)
Transmission prob. F-to-M, non-spousal	2950 million (2770 to 3120 million)
RR transmission if spousal (M-to-F)	562 334 (480 071 to 643 201)
RR transmission if spousal (F-to-M)	867 765 (793 635 to 945 292)
Initial HIV prevalence in high risk women	-6.87 million (-11.40 to -2.67 million)
Sexual mixing	108 196 (18 851 to 202 810)
Increased infectivity at CD4 <200 cells/µl	924 177 (880 687 to 969 362)
Transmission prob. M-to-M, non-spousal	13.7 million (-19.7 to 39.4 million)
RR transmission if spousal (M-to-M)	-123 559 (-203 273 to -46 928)

The results of the second regression are shown in Table S3.15. As suggested previously, the projected future HIV incidence (in the absence of any change to HIV testing policy) is strongly negatively related to the cost per life year saved: for each additional increase of 1000 new infections over the 2019-39 period, the cost per life year saved reduces by $0.22. The intervention-specific parameters have similar effects to those shown previously (in Tables S3.11 and S3.13), where the regression model controlled for the individual baseline parameters rather than the total number of new infections over the 2019-39 period.

Table S3.15: Effects of new infections and intervention parameters on the incremental cost per life year saved, for home-based testing with an offer of self-testing

Parameter	Effect (95% CI)
New HIV infections (2019-39) if no change in policy	-0.00022 (-0.00025 to -0.00019)
RR testing in previously-diagnosed, ART-naïve	-238 (-273 to -202)
RR testing in ART patients	173 (48 to 338)
RR linkage to ART from community-based testing	-210 (-279 to -130)
Fraction of population tested in each round of home-based testing	-82 (-248 to 159)
Effect of self-testing offer on uptake of testing	-2 (-24 to 19)

As an additional sensitivity analysis, we assessed the sensitivity of the results to the levels of HIV testing in the baseline scenario. PEPFAR, which funds roughly half of the South African HIV testing programme, has called for a scaling back of HIV testing efforts, particularly in the context of general provider-initiated counselling and testing [186]. We have therefore assessed how results would change in the baseline scenario and the home-based testing with self-testing scenario if rates of ‘general’ HIV testing were halved from 2019 onward. This implies reducing

the $b(t)$ parameter to 0.0724 in men and 0.1411 in women, over the 2019-39 period (compare with the default values of 0.1448 and 0.2821 respectively in Table S2.1). Table S3.16 compares the results in the main analyses (presented previously) with the results in the sensitivity analysis. In the baseline scenario, the 50% reduction in the rate of general HIV testing would result in a 33% reduction in the total number of HIV tests conducted over the 2019-2039 period (from 274 million to 185 million) but only a modest 2% reduction in the number of new HIV diagnoses and a 3% increase in the total number of new HIV infections. The net effect of the increase in HIV incidence and reduction in new diagnoses is a reduction in the fraction of the HIV-positive population that is diagnosed by 2030 (from 93.9% in the main analysis to 91.8% in the sensitivity analysis). Results change in the home-based testing with self-testing scenario: the increase in the number of new diagnoses (relative to baseline) changes from 103 000 in the main analysis to 187 000 in the sensitivity analysis, i.e. the total number of new diagnoses remains unchanged in the home-based testing with self-testing scenario (at around 5.95 million) but relatively more of the testing occurs through home-based testing in the sensitivity analysis. Since home-based testing with self-testing is significantly cheaper than provider-initiated testing in facilities, this implies a gain in efficiency and thus a reduction in the cost per life year saved (from $394 to $316 for home-based testing with self-testing) when there is a reduction in provider-initiated testing in health facilities. Nevertheless, there are more new HIV infections forecast when the frequency of provider-initiated testing is reduced (5.32 million versus 5.26 million).

Table S3.16: Comparison of results in main analysis and sensitivity analysis with 50% reduction in 'general' HIV testing in the baseline scenario, 2019-2039

	Main analysis	Sensitivity analysis
Baseline scenario		
Total HIV tests (million)	273.6 (272.9-274.2)	184.5 (184.1-185.0)
New HIV diagnoses (million)	5.85 (5.79-5.91)	5.77 (5.71-5.83)
New HIV infections (million)	5.53 (5.47-5.96)	5.68 (5.62-5.74)
Fraction diagnosed by 2030	93.9%	91.8%
Total cost of HIV programme (billion USD)	37.7 (37.4-37.9)	36.9 (36.7-37.2)
Home-based testing + self-testing scenario		
Increase in total tests (million)*	429.3 (427.5-431.0)	428.3 (426.5-430.0)
Increase in new diagnoses (thousand)*	103 (81-126)	187 (165-210)
New HIV infections (million)	5.26 (5.20-5.31)	5.32 (5.26-5.37)
Fraction diagnosed by 2030	96.5%	96.1%
Total cost of HIV programme (billion USD)	39.6 (39.4-39.8)	39.0 (38.8-39.3)
Cost-effectiveness of home-based testing + self-testing*		
Life years saved (millions)	4.83 (4.70-4.98)	6.74 (6.58-6.91)
HIV cases averted (thousands)	268 (249-288)	354 (336-372)
Cost per life year saved (USD)	394 (379-410)	316 (305-325)
Cost per HIV infected averted (USD)	7118 (6512-7766)	6006 (5642-6385)

* Relative to baseline.

4. Comparison with other modelling studies

To our knowledge, only one previous study has modelled a wide range of different HIV testing strategies [187]. Table S4.1 compares the features and results of this modelling exercise, by Avenir Health, with our own. The two models are structurally very different, with the Avenir Health model dividing the population into 12 different possible testing populations (defined in terms of the channel through which individuals are likely to access testing), while our model is individual-based, allowing for individuals to access HIV testing through multiple channels. Despite substantial methodological differences, the Avenir Health study came to similar conclusions as ours regarding the relative efficiency of different HIV testing strategies. Both studies concluded that the most efficient strategies would include testing partners of newly-diagnosed individuals, patients with HIV symptoms, sex workers and MSM, and both studies concluded that although community-based HIV testing strategies were not generally very cost-effective, self-testing could be a relatively cost-effective strategy to reach individuals with high HIV risk in community settings, because of the cost savings if healthworkers have fewer tests to perform.

Table S4.1: Comparison of models of HIV testing strategies

	Avenir Health model	MicroCOSM
Settings	Mozambique, Senegal, Nigeria, Bolivia	South Africa
Model type	Deterministic, compartmental	Agent-based, network
Simulation start	2015	1985
HIV transmission	Not modelled. Within each model compartment, HIV prevalence is assumed to remain constant over time.	Modelled dynamically, based on assumed sexual behaviour and HIV transmission probabilities
HIV disease progression	No modelling of CD4 stage	Individual-level variation in HIV viral load and CD4 count, which determines mortality risk and rate of HIV symptoms
Calibration to HIV testing data	DHS/AIS data: % of adults ever tested for HIV, or tested in last year	Total annual numbers of HIV tests performed, HIV prevalence in testers
HIV testing modalities	Antenatal clinics<br>Family planning clinics<br>STI clinics<br>Patients with HIV symptoms<br>General health services<br>Sex workers<br>Men who have sex with men<br>Home-based testing*<br>Partners of HIV-diagnosed<br>TB patients<br>Injecting drug users	Antenatal clinics<br>Family planning clinics<br>STI clinics<br>Patients with HIV symptoms<br>General health services<br>Sex workers<br>Men who have sex with men<br>Home-based testing<br>Partners of HIV-diagnosed<br>Mobile testing<br>Schools<br>Workplaces<br>Men seeking MMC<br>Prisons<br>Partners of pregnant women<br>PrEP recipients
Self-testing	Modelled as a variation of home-based testing*, with higher yield assumed	Modelled in the context of home- based testing, testing partners of pregnant women
Test sensitivity and specificity	100% sensitivity and specificity	100% sensitivity and specificity, except in acute infection (0% sensitivity)
Retesting of previously diagnosed	Not modelled	For baseline testing modalities, diagnosed untreated are assumed to have a 50% relative rate of HIV testing.
ART initiation after diagnosis	90%, but varying between 50% and 80% in the case of community-based testing	Rate of ART initiation varies (27-93%), depending on the HIV testing modality
Highest yield testing modalities	Partners of HIV-diagnosed<br>TB patients<br>Sex workers<br>Patients with HIV symptoms<br>STI patients<br>Men who have sex with men	Partners of HIV-diagnosed<br>Patients with HIV symptoms<br>Sex workers<br>Partners of HIV+ pregnant women<br>Workplaces<br>PrEP recipients<br>Men who have sex with men
Lowest yield testing modalities	Home-based testing*<br>Family planning	Home-based testing<br>Schools<br>Prisons

* The authors use the term 'community-based testing', which encompasses home-based testing and other testing modalities (e.g. mobile testing and multi-disease screening interventions).

Cambiano *et al* [188] have also modelled the impact of self-testing in the Zimbabwean setting. Although they estimated that self-testing could be cost saving, as a result of reductions in health worker time required for HIV testing through other modalities, they noted that their conclusions were sensitive to assumptions about the cost of self-testing kits, the sensitivity and specificity of the testing kits, and the relative rate of linkage to care in individuals who are self-diagnosed.

Bassett *et al* [189] have used the CEPAC model to evaluate the potential impact of mobile testing in the South African setting. Their results suggest that the addition of mobile HIV testing to a standard model of HIV testing in fixed health facilities is likely to be very cost-effective relative to South Africa's per capita GDP. A limitation of this model is that it does not consider the effect of HIV diagnosis and increased ART initiation on HIV transmission, and the benefits of HIV testing may therefore be understated.

Several models have been developed to simulate the potential impact of home-based testing. Although a number of these studies have concluded that home-based testing would be cost-effective [8, 190], the absence of any comparison to other HIV testing strategies makes it difficult to assess the relative merit of home-based testing. Most of these models assume that linkage to care following home-based testing is the same as that in facility-based HIV testing. However, Olney *et al* [191] developed a model that assumed a lower rate of linkage to care in individuals who were diagnosed through home-based testing, and estimated that home-based HIV testing would have relatively low cost-effectiveness in this scenario.

References

1. World Health Organization. Guidance on provider-initiated HIV testing and counselling in health facilities. Geneva; 2007. Available: http://www.who.int/hiv/topics/vct/en/index.html. Accessed 10 Dec 2008
2. Lissouba P, Taljaard D, Rech D, Doyle S, Shabangu D, Nhlapo C, *et al.* A model for the roll-out of comprehensive adult male circumcision services in African low-income settings of high HIV incidence: the ANRS 12126 Bophelo Pele Project. *PLoS Med* 2010; **7**:e1000309.
3. Richter M, Venter WD, Gray A. Home self-testing for HIV: AIDS exceptionalism gone wrong. *S Afr Med J* 2010; **100**:636-642.
4. Meehan SA, Naidoo P, Claassens MM, Lombard C, Beyers N. Characteristics of clients who access mobile compared to clinic HIV counselling and testing services: a matched study from Cape Town, South Africa. *BMC Health Serv Res* 2014; **14**:658.
5. Meyer-Rath G, van Rensburg C, Chiu C, Cohen S. The per-patient costs of HIV services in South Africa: Systematic review and application in the South African HIV Investment Case (forthcoming). 2017.
6. Department of Health, South African National AIDS Council. South African HIV and TB Investment Case - Summary Report Phase 1. 2016. Available: http://sanac.org.za/wp-content/uploads/2016/03/1603-Investment-Case-Report-LowRes-18-Mar.pdf. Accessed 31 May 2016
7. Tabana H, Nkonki L, Hongoro C, Doherty T, Ekstrom AM, Naik R, *et al.* A Cost-Effectiveness Analysis of a Home-Based HIV Counselling and Testing Intervention versus the Standard (Facility Based) HIV Testing Strategy in Rural South Africa. *PLoS One* 2015; **10**:e0135048.
8. Smith JA, Sharma M, Levin C, Baeten JM, van Rooyen H, Celum C, *et al.* Cost-effectiveness of community-based strategies to strengthen the continuum of HIV care in rural South Africa: a health economic modelling analysis. *Lancet HIV* 2015; **2**:e159-e168.
9. Rutstein SE, Brown LB, Biddle AK, Wheeler SB, Kamanga G, Mmodzi P, *et al.* Cost-effectiveness of provider-based HIV partner notification in urban Malawi. *Health Policy Plan* 2014; **29**:115-126.
10. Johnson LF, Kubjane M, Moolla H. MicroCOSM: a model of social and structural drivers of HIV and interventions to reduce HIV incidence in high-risk populations in South Africa. *BioRxiv* 2018.
11. Johnson LF, Geffen N. A comparison of two mathematical modeling frameworks for evaluating sexually transmitted infection epidemiology. *Sex Transm Dis* 2016; **43**:139-146.
12. Actuarial Society of South Africa. ASSA2008 AIDS and Demographic Model. 2011. Available: http://aids.actuarialsociety.org.za. Accessed 5 April 2011
13. Shisana O, Rehle T, Simbayi LC, Parker W, Zuma K, Bhana A, *et al.* South African National HIV Prevalence, HIV Incidence, Behaviours and Communication Survey, 2005. Cape Town: HSRC Press; 2005. Available: http://www.hsrcpress.ac.za. Accessed 1 Dec 2005
14. Shisana O, Rehle T, Simbayi LC, Zuma K, Jooste S, Pillay-van Wyk V, *et al.* South African national HIV prevalence, incidence, behaviour and communication survey, 2008: A turning tide among teenagers? Cape Town: Human Sciences Research Council; 2009. Available: http://www.hsrcpress.ac.za. Accessed 9 June 2009

15. Shisana O, Rehle T, Simbayi LC, Zuma K, Jooste S, Zungu N, *et al.* South African National HIV Prevalence, Incidence, and Behaviour Survey, 2012. Cape Town: Human Sciences Research Council; 2014. Available: http://www.hsrc.ac.za/en/research-outputs/view/6871. Accessed 16 April 2014
16. Department of Health. The 2012 National Antenatal Sentinel HIV and Herpes Simplex Type-2 Prevalence Survey in South Africa. 2014. Available: http://www.health.gov.za/reports.php. Accessed 14 May 2014
17. Johnson LF, Mulongeni P, Marr A, Lane T. Age bias in survey sampling and implications for estimating HIV prevalence in men who have sex with men: insights from mathematical modelling. *Epidemiol Infect* 2018; **146**:1036-1042.
18. Lim'uvune Consulting. Department of Correctional Services HIV Prevalence Survey 2006. 2008.
19. Telisinghe L, Fielding KL, Malden JL, Hanifa Y, Churchyard GJ, Grant AD, *et al.* High tuberculosis prevalence in a South African prison: the need for routine tuberculosis screening. *PLoS One* 2014; **9**:e87262.
20. Skiti V, Goodman E, Gribble P, Hausler H. Screening and testing for tuberculosis and HIV in correctional facilities in the Western Cape, South Africa [Abstract PC-451-01]. *44th World Conference on Lung Health*. Paris, France; 2013.
21. Johnson LF, Dorrington RE, Moolla H. Progress towards the 2020 targets for HIV diagnosis and antiretroviral treatment in South Africa. *South Afr J HIV Med* 2017; **18**:a694.
22. Johnson LF, Rehle TM, Jooste S, Bekker LG. Rates of HIV testing and diagnosis in South Africa, 2002-2012: successes and challenges. *AIDS* 2015; **29**:1401-1409.
23. Maughan-Brown B, Lloyd N, Bor J, Venkataramani AS. Changes in self-reported HIV testing during South Africa's 2010/2011 national testing campaign: gains and shortfalls. *J Int AIDS Soc* 2016; **19**:20658.
24. Venkatesh KK, Madiba P, De Bruyn G, Lurie MN, Coates TJ, Gray GE. Who gets tested for HIV in a South African urban township? Implications for test and treat and gender-based prevention interventions. *J Acquir Immun Defic Syndr* 2011; **56**:151-165.
25. Hutchinson PL, Mahlalela X. Utilization of voluntary counseling and testing services in the Eastern Cape, South Africa. *AIDS Care* 2006; **18**:446-455.
26. Mfundisi C, Chiranjan N, Rodrigues C, Kirchner L, Bock P, Myer L. Availability of antiretroviral therapy is associated with increased uptake of HIV testing services. *S Afr Med J* 2005; **95**:483-485.
27. Mitchell S, Cockcroft A, Lamothe G, Andersson N. Equity in HIV testing: evidence from a cross-sectional study in ten Southern African countries. *BMC Int Health Hum Rights* 2010; **10**:23.
28. Pettifor A, MacPhail C, Suchindran S, Delany-Moretlwe S. Factors associated with HIV testing among public sector clinic attendees in Johannesburg, South Africa. *AIDS Behav* 2010; **14**:913-921.
29. Dalal S, Lee CW, Farirai T, Schilsky A, Goldman T, Moore J, *et al.* Provider-initiated HIV testing and counseling: increased uptake in two public community health centers in South Africa and implications for scale-up. *PLoS One* 2011; **6**:e27293.
30. Luseno WK, Wechsberg WM. Correlates of HIV testing among South African women with high sexual and substance-use risk behaviours. *AIDS Care* 2009; **21**:178-184.
31. Johnson LF, Dorrington RE. Thembisa version 4.1: A model for evaluating the impact of HIV/AIDS in South Africa. 2018.

32. Holmes CB, Wood R, Badri M, Zilber S, Wang B, Maartens G, *et al.* CD4 decline and incidence of opportunistic infections in Cape Town, South Africa: implications for prophylaxis and treatment. *J Acquir Immun Defic Syndr* 2006; **42**:464-469.
33. Anglaret X, Minga A, Gabillard D, Ouassa T, Messou E, Morris B, *et al.* AIDS and non-AIDS morbidity and mortality across the spectrum of CD4 cell counts in HIV-infected adults before starting antiretroviral therapy in Côte d'Ivoire. *Clin Infect Dis* 2012; **54**:714-723.
34. World Health Organization. Global Tuberculosis Report 2016. 2016. Available: http://www.who.int/tb/publications/global_report/en/. Accessed 9 Aug 2017
35. Badri M, Bekker LG, Orrell C, Pitt J, Cilliers F, Wood R. Initiating highly active antiretroviral therapy in sub-Saharan Africa: an assessment of the revised World Health Organization scaling-up guidelines. *AIDS* 2004; **18**:1159-1168.
36. Murphy E, Collier A, Kalish L, Assman S, Para M, Flanigan T, *et al.* Highly active antiretroviral therapy decreases mortality and morbidity in patients with advanced HIV disease. *Ann Intern Med* 2001; **135**:17-26.
37. Palella F, Delaney K, Moorman A, Loveless M, Fuhrer J, Satten G, *et al.* Declining morbidity and mortality among patients with advanced human immunodeficiency virus infection. *N Engl J Med* 1998; **338**:853-860.
38. McCoy D, Besser M, Visser R, Doherty T. Interim findings on the national PMTCT pilot sites: lessons and recommendations. Durban: Health Systems Trust; 2002. Available: http://www.hst.org.za/publications/478. Accessed 9 April 2006
39. Ramkissoon A, Kleinschmidt I, Beksinska M, Smit J, Hlazo J, Mabude Z. National Baseline Assessment of Sexually Transmitted Infection and HIV Services in South African Public Sector Health Facilities. Durban: Reproductive Health Research Unit; 2004. Available: http://www.rhru.co.za. Accessed 13 February 2004
40. Reagon G, Irlam J, Levin J. The National Primary Health Care Facilities Survey 2003. Durban: Health Systems Trust; 2004. Available: http://www.hst.org.za/publications/617. Accessed 6 Aug 2010
41. World Health Organization. Global Tuberculosis Control 2009: Epidemiology, Strategy, Financing. Geneva; 2009. Available: http://www.who.int/tb/publications/global_report/2009/pdf/full_report.pdf. Accessed 5 March 2012
42. Barron P, Day C, Loveday M, Monticelli F. The District Health Barometer Year 1: January-December 2004. Durban: Health Systems Trust; 2005. Available: http://www.hst.org.za/publications/689. Accessed 29 Dec 2008
43. World Health Organization. Global Tuberculosis Control 2010. Geneva; 2010. Available: http://www.who.int/tb/publications/global_report/2010/en/index.html. Accessed 12 March 2012
44. Barron P, Day C, Monticelli F, Vermaak K, Okorafor O, Moodley K, *et al.* District Health Barometer 2005/06. Health Systems Trust; 2006. Available: http://www.hst.org.za/publications/701. Accessed 15 March 2007
45. Barron P, Day C, Monticelli F. The Disrict Health Barometer - Year 2006/07. Health Systems Trust; 2008. Available: http://www.hst.org.za/publications/717. Accessed 22 Feb 2008
46. Kigozi NG, Heunis JC, Wouters E, van den Berg HS. Tuberculosis patients' reasons for, and suggestions to address non-uptake of HIV testing: a cross-sectional study in the Free State Province, South Africa. *BMC Health Serv Res* 2011; **11**:110.

47. Day C, Barron P, Monticelli F, Sello E. District Health Barometer 2007/08. Health Systems Trust; 2009. Available: http://www.hst.org.za/publications/850. Accessed 10 July 2009
48. Orie EF, Songca PP, Moodley J. An audit of PMTCT services at a regional hospital in South Africa. *S Afr Fam Pract* 2009; **51**:492-495.
49. Day C, Monticelli F, Barron P, Haynes R, Smith J, Sello E. District Health Barometer: Year 2008/09. Durban: Health Systems Trust; 2010. Available: http://www.hst.org.za/publications/864. Accessed 25 June 2010
50. Massyn N, Peer N, English R, Padarath A, Barron P, Day C. District Health Barometer 2015/16. Durban; 2016. Available: http://www.hst.org.za/publications/district-health-barometer-201516-0. Accessed 5 March 2017
51. Grimwood A, Fatti G, Mothibi E, Eley B, Jackson D. Progress of preventing mother-to-child transmission of HIV at primary healthcare facilities and district hospitals in three South African provinces. *S Afr Med J* 2012; **102**:81-83.
52. World Health Organization. Global Tuberculosis Control 2011. Geneva; 2011. Available: http://whqlibdoc.who.int/publications/2011/9789241564380_eng.pdf. Accessed 12 March 2012
53. Goga AE, Dinh TH, Jackson DJ. Evaluation of the Effectiveness of the National Prevention of Mother-to-Child Transmission (PMTCT) Programme Measured at Six Weeks Postpartum in South Africa, 2010. South African Medical Research Council, National Department of Health of South Africa and PEPFAR/US Centers for Disease Control and Prevention; 2012. Available: http://www.doh.gov.za/docs/reports/2012/pmtcteffectiveness.pdf. Accessed 12 June 2012
54. Massyn N, Day C, Barron P, Haynes R, English R, Padarath A. District Health Barometer 2011/12. Durban: Health Systems Trust; 2013. Available: http://www.hst.org.za/publications/district-health-barometer-201112. Accessed 23 Oct 2013
55. Makin JD, Forsyth BW, Visser MJ, Sikkema KJ, Neufeld S, Jeffery B. Factors affecting disclosure in South African HIV-positive pregnant women. *AIDS Patient Care STDs* 2008; **22**:907-916.
56. Vu L, Andrinopoulos K, Mathews C, Chopra M, Kendall C, Eisele TP. Disclosure of HIV status to sex partners among HIV-infected men and women in Cape Town, South Africa. *AIDS Behav* 2012; **16**:132-138.
57. Mkwanazi NB, Rochat TJ, Bland RM. Living with HIV, disclosure patterns and partnerships a decade after the introduction of HIV programmes in rural South Africa. *AIDS Care* 2015; **27 (Suppl 1)**:65-72.
58. Haberlen SA, Nakigozi G, Gray RH, Brahmbhatt H, Ssekasanvu J, Serwadda D, *et al.* Antiretroviral therapy availability and HIV disclosure to spouse in Rakai, Uganda: a longitudinal population-based study. *J Acquir Immun Defic Syndr* 2015; **69**:241-247.
59. Myer L, Morroni C, Rebe K. Prevalence and determinants of fertility intentions of HIV-infected women and men receiving antiretroviral therapy in South Africa. *AIDS Patient Care STDs* 2007; **21**:278-285.
60. Ostermann J, Pence B, Whetten K, Yao J, Itemba D, Maro V, *et al.* HIV serostatus disclosure in the treatment cascade: evidence from Northern Tanzania. *AIDS Care* 2015; **27 (Suppl 1)**:59-64.

61. Brown LB, Miller WC, Kamanga G, Nyirenda N, Mmodzi P, Pettifor A, *et al.* HIV partner notification is effective and feasible in sub-Saharan Africa: opportunities for HIV treatment and prevention. *J Acquir Immun Defic Syndr* 2011; **56**:437-442.
62. Kiene SM, Bateganya M, Wanyenze R, Lule H, Nantaba H, Stein MD. Initial outcomes of provider-initiated routine HIV testing and counseling during outpatient care at a rural Ugandan hospital: risky sexual behavior, partner HIV testing, disclosure, and HIV care seeking. *AIDS Patient Care STDs* 2010; **24**:117-126.
63. Msuya SE, Mbizvo EM, Hussain A, Uriyo J, Sam NE, Stray-Pedersen B. Low male partner participation in antenatal HIV counselling and testing in northern Tanzania: implications for preventive programs. *AIDS Care* 2008; **20**:700-709.
64. Aluisio A, Richardson BA, Bosire R, John-Stewart G, Mbori-Ngacha D, Farquhar C. Male antenatal attendance and HIV testing are associated with decreased infant HIV infection and increased HIV-free survival. *J Acquir Immun Defic Syndr* 2011; **56**:76-82.
65. Rosenberg NE, Mtande TK, Saidi F, Stanley C, Jere E, Paile L, *et al.* Recruiting male partners for couple HIV testing and counselling in Malawi's option B+ programme: an unblinded randomised controlled trial. *Lancet HIV* 2015; **2**:e483-491.
66. Department of Health. Protocols for the management of a person with a sexually transmitted disease. Pretoria; 1997. Available: http://www.kznhealth.gov.za/STDprotocols.pdf. Accessed 16 April 2008
67. Department of Health. Standard Treatment Guidelines and Essential Drugs List for South Africa. 3rd ed. Pretoria; 2003. Available: http://www.kznhealth.gov.za/edlphc2003.pdf. Accessed 16 April 2008
68. Department of Health. First Line Comprehensive Management and Control of Sexually Transmitted Infections (STIs). Pretoria; 2009. Available: http://www.nicd.ac.za/units/stirc. Accessed 10 July 2009
69. Kohler PK, Marumo E, Jed SL, Mema G, Galagan S, Tapia K, *et al.* A national evaluation using standardised patient actors to assess STI services in public sector clinical sentinel surveillance facilities in South Africa. *Sex Transm Infect* 2017; **93**:247-252.
70. Weaver MR, Pillay E, Jed SL, de Kadt J, Galagan S, Gilvydis J, *et al.* Three methods of delivering clinic-based training on syndromic management of sexually transmitted diseases in South Africa: a pilot study. *Sex Transm Infect* 2016; **92**:135-141.
71. Leon N, Naidoo P, Mathews C, Lewin S, Lombard C. The impact of provider-initiated (opt-out) HIV testing and counseling of patients with sexually transmitted infection in Cape Town, South Africa: a controlled trial. *Implement Sci* 2010; **5**:8.
72. Kharsany AB, Karim QA, Karim SS. Uptake of provider-initiated HIV testing and counseling among women attending an urban sexually transmitted disease clinic in South Africa - missed opportunities for early diagnosis of HIV infection. *AIDS Care* 2010; **22**:533-537.
73. Schneider H, Blaauw D, Dartnall E, Coetzee DJ, Ballard RC. STD care in the South African private health sector. *S Afr Med J* 2001; **91**:151-156.
74. Department of Correctional Services. Annual report for the 2007/2008 financial year. 2008. Available: http://www.dcs.gov.za/Publications/AnnualReports.aspx. Accessed 14 Oct 2016
75. Department of Correctional Services. Annual report for the 2009/2010 financial year. 2010. Available: http://www.dcs.gov.za/Publications/Annual%20Reports/DCS%20Annual%20Report%202010.pdf. Accessed 15 Feb 2013

76. Department of Correctional Services. Annual Report 2012/2013. 2013. Available: http://www.dcs.gov.za/Publications/AnnualReports.aspx. Accessed 23 June 2017
77. Department of Correctional Services. Annual Report 2013/2014. 2014. Available: http://www.dcs.gov.za/Publications/AnnualReports.aspx. Accessed 23 June 2017
78. Department of Correctional Services. Annual Report: 2014/2015 financial year. http://www.dcs.gov.za/Publications/AnnualReports.aspx. Accessed 14 Oct 2016; 2015.
79. Johnson LF, Dorrington RE, Bradshaw D, Coetzee DJ. The effect of syndromic management interventions on the prevalence of sexually transmitted infections in South Africa. *Sex Reprod Healthc* 2011; **2**:13-20.
80. World Health Organization. Operational guidance for scaling up male circumcision services for HIV prevention. 2008. Available: http://www.who.int/hiv/pub/malecircumcision/op_guidance/en/index.html. Accessed 25 Jan 2013
81. Kikaya V, Skolnik L, Garcia MC, Nkonyana J, Curran K, Ashengo TA. Voluntary medical male circumcision programs can address low HIV testing and counseling usage and ART enrollment among young men: lessons from Lesotho. *PLoS One* 2014; **9**:e83614.
82. Phili R, Abdool Karim Q, Tlou B. Experiences in the implementation of provider-initiated counselling and testing and linkage to HIV services at urban public sector health facilities in KwaZulu-Natal. *Southern African Journal of Infectious Diseases* 2015; **30**:77-81.
83. Jean K, Puren A, Cutler E, Singh B, Bouscaillou J, Rain-Taljaard R, *et al.* Level of viral suppression and the cascade of HIV care in a South African semi-urban setting in 2012. *AIDS* 2016; **30**:2107-2116.
84. Johnson LF, Chiu C, Myer L, Davies MA, Dorrington RE, Bekker LG, *et al.* Prospects for HIV control in South Africa: a model-based analysis. *Glob Health Action* 2016; **9**:30314.
85. Department of Health. Annual Report 2015/16. Pretoria; 2016. Available: http://www.health.gov.za/index.php/2014-03-17-09-09-38/2014-03-17-09-24-31. Accessed 6 Feb 2017
86. Department of Health. Annual Report 2014/15. Pretoria; 2015. Available: http://www.health.gov.za/index.php/2014-03-17-09-09-38/2014-03-17-09-24-31/category/239-ar2015. Accessed 1 Jan 2016
87. Department of Health. Annual Report 2013-2014. 2014. Available: http://www.health.gov.za/annualreports.php. Accessed 25 Jan 2015
88. Kripke K, Chen PA, Vazzano A, Thambinayagam A, Pillay Y, Loykissoonlal D, *et al.* Cost and impact of voluntary medical male circumcision in South Africa: focusing the program on specific age groups and provinces. *PLoS One* 2016; **11**:e0157071.
89. Eakle R, Gomez GB, Naicker N, Bothma R, Mbogua J, Cabrera Escobar MA, *et al.* HIV pre-exposure prophylaxis and early antiretroviral treatment among female sex workers in South Africa: Results from a prospective observational demonstration project. *PLoS Med* 2017; **14**:e1002444.
90. Bekker LG, Rebe K, Venter F, Maartens G, Moorhouse M, Conradie F, *et al.* South African guidelines on the safe use of pre-exposure prophylaxis in persons at risk of acquiring HIV-1 infection. *South Afr J HIV Med* 2016; **17**:455.
91. Fuente-Soro L, Lopez-Varela E, Augusto O, Sacoor C, Nhacolo A, Honwana N, *et al.* Monitoring progress towards the first UNAIDS target: understanding the impact of

people living with HIV who re-test during HIV-testing campaigns in rural Mozambique. *J Int AIDS Soc* 2018; **21**:e25095.
92. Cherutich P, Golden MR, Wamuti B, Richardson BA, Ásbjörnsdóttir KH, Otieno FA, *et al.* Assisted partner services for HIV in Kenya: a cluster randomised controlled trial. *Lancet HIV* 2017; **4**:e74-e82.
93. Henley C, Forgwei G, Welty T, Golden M, Adimora A, Shields R, *et al.* Scale-up and case-finding effectiveness of an HIV partner services program in Cameroon: an innovative HIV prevention intervention for developing countries. *Sex Transm Dis* 2013; **40**:909-914.
94. Dalal S, Johnson C, Fonner V, Kennedy CE, Siegfried N, Figueroa C, *et al.* Improving HIV test uptake and case finding with assisted partner notification services. *AIDS* 2017; **31**:1867-1876.
95. Naik R, Tabana H, Doherty T, Zembe W, Jackson D. Client characteristics and acceptability of a home-based HIV counselling and testing intervention in rural South Africa. *BMC Public Health* 2012; **12**:824.
96. Maheswaran H, Thulare H, Stanistreet D, Tanser F, Newell ML. Starting a home and mobile HIV testing service in a rural area of South Africa. *J Acquir Immun Defic Syndr* 2012; **59**:e43-46.
97. González R, Munguambe K, Aponte J, Bavo C, Nhalungo D, Macete E, *et al.* High HIV prevalence in a southern semi-rural area of Mozambique: a community-based survey. *HIV Med* 2012; **13**:581-588.
98. Doherty T, Tabana H, Jackson D, Naik R, Zembe W, Lombard C, *et al.* Effect of home based HIV counselling and testing intervention in rural South Africa: cluster randomised trial. *BMJ* 2013; **346**:f3481.
99. Sharma M, Ying R, Tarr G, Barnabas R. Systematic review and meta-analysis of community and facility-based HIV testing to address linkage to care gaps in sub-Saharan Africa. *Nature* 2015; **528**:S77-85.
100. Parker LA, Jobanputra K, Rusike L, Mazibuko S, Okello V, Kerschberger B, *et al.* Feasibility and effectiveness of two community-based HIV testing models in rural Swaziland. *Trop Med Int Health* 2015; **20**:893-902.
101. Tumwebaze H, Tumwesigye E, Baeten JM, Kurth AE, Revall J, Murnane PM, *et al.* Household-based HIV counseling and testing as a platform for referral to HIV care and medical male circumcision in Uganda: a pilot evaluation. *PLoS One* 2012; **7**:e51620.
102. Sekandi JN, Sempeera H, List J, Mugerwa MA, Asiimwe S, Yin X, *et al.* High acceptance of home-based HIV counseling and testing in an urban community setting in Uganda. *BMC Public Health* 2011; **11**:730.
103. Bogart LM, Wagner GJ, Musoke W, Naigino R, Linnemayr S, Maistrellis E, *et al.* A comparison of home-based versus outreach event-based community HIV testing in Ugandan fisherfolk communities. *AIDS Behav* 2017; **21**:547-560.
104. Shanaube K, Schaap A, Floyd S, Phiri M, Griffith S, Chaila J, *et al.* What works - reaching universal HIV testing: lessons from HPTN 071 (PopART) trial in Zambia. *AIDS* 2017; **31**:1555-1564.
105. Fylkesnes K, Sandøy IF, Jürgensen M, Chipimo PJ, Mwangala S, Michelo C. Strong effects of home-based voluntary HIV counselling and testing on acceptance and equity: a cluster randomised trial in Zambia. *Soc Sci Med* 2013; **86**:9-16.
106. Helleringer S, Mkandawire J, Reniers G, Kalilani-Phiri L, Kohler HP. Should home-based HIV testing and counseling services be offered periodically in programs of

ARV treatment as prevention? A case study in Likoma (Malawi). *AIDS Behav* 2013; **17**:2100-2108.

107. Granich RM, Gilks CF, Dye C, De Cock KM, Williams BG. Universal voluntary HIV testing with immediate antiretroviral therapy as a strategy for elimination of HIV transmission: a mathematical model. *Lancet* 2009; **373**:48-57.

108. Dodd PJ, Garnett GP, Hallett TB. Examining the promise of HIV elimination by 'test and treat' in hyperendemic settings. *AIDS* 2010; **24**:729-735.

109. Department of Health. National HIV counselling and testing policy guidelines. 2015. Available: https://www.scribd.com/doc/268967963/Guidelines-National-HIV-Counselling-and-Testing-HCT-Policy-Guidelines-2015#download. Accessed 10 Aug 2015

110. Liambila W, Askew I, Mwangi J, Ayisi R, Kibaru J, Mullick S. Feasibility and effectiveness of integrating provider-initiated testing and counselling within family planning services in Kenya. *AIDS* 2009; **23 (Suppl 1)**:S115-121.

111. Church K, Warren CE, Birdthistle I, Ploubidis GB, Tomlin K, Zhou W, *et al.* Impact of integrated services on HIV testing: A nonrandomized trial among Kenyan family planning clients. *Stud Fam Plann* 2017; **48**:201-218.

112. Kranzer K, Govindasamy D, van Schaik N, Thebus E, Davies N, Zimmermann M, *et al.* Incentivized recruitment of a population sample to a mobile HIV testing service increases the yield of newly diagnosed cases, including those in need of antiretroviral therapy. *HIV Med* 2012; **13**:132-137.

113. Bassett IV, Regan S, Mbonambi H, Blossom J, Bogan S, Bearnot B, *et al.* Finding HIV in hard to reach populations: mobile HIV testing and geospatial mapping in Umlazi township, Durban, South Africa. *AIDS Behav* 2015; **19**:1888-1895.

114. Sweat M, Morin S, Celentano D, Mulawa M, Singh B, Mbwambo J, *et al.* Community-based intervention to increase HIV testing and case detection in people aged 16-32 years in Tanzania, Zimbabwe, and Thailand (NIMH Project Accept, HPTN 043): a randomised study. *Lancet Infect Dis* 2011; **11**:525-532.

115. Ostermann J, Reddy EA, Shorter MM, Muiruri C, Mtalo A, Itemba DK, *et al.* Who tests, who doesn't, and why? Uptake of mobile HIV counseling and testing in the Kilimanjaro region of Tanzania. *PLoS One* 2011; **6**:e16488.

116. Kranzer K, Lawn SD, Johnson LF, Bekker LG, Wood R. Community viral load and CD4 count distribution among people living with HIV in a South African township: implications for treatment as prevention. *J Acquir Immun Defic Syndr* 2013; **63**:498-505.

117. Kawichai S, Celentano DD, Chariyalertsak S, Visrutaratna S, Short O, Ruangyuttikarn C, *et al.* Community-based voluntary counseling and testing services in rural communities of Chiang Mai Province, Northern Thailand. *AIDS Behav* 2007; **11**:770-777.

118. Lippman SA, Neilands TB, MacPhail C, Peacock D, Maman S, Rebombo D, *et al.* Community mobilization for HIV testing uptake: results from a community randomized trial of a theory-based intervention in rural South Africa. *J Acquir Immun Defic Syndr* 2017; **74 (Suppl 1)**:S44-S51.

119. Adebajo S, Eluwa G, Njab J, Oginni A, Ukwuije F, Ahonsi B, *et al.* Evaluating the effect of HIV prevention strategies on uptake of HIV counselling and testing among male most-at-risk-populations in Nigeria; a cross-sectional analysis. *Sex Transm Infect* 2015; **91**:555-560.

120. Geibel S, King'ola N, Temmerman M, Luchters S. The impact of peer outreach on HIV knowledge and prevention behaviours of male sex workers in Mombasa, Kenya. *Sex Transm Infect* 2012; **88**:357-362.
121. Lane T, Osmand T, Marr A, Shade SB, Dunkle K, Sandfort T, *et al.* The Mpumalanga Men's Study (MPMS): Results of a baseline biological and behavioral HIV surveillance survey in two MSM communities in South Africa. *PLoS One* 2014; **9**:e111063.
122. Lane T, Osmand T, Marr A, Struthers H, McIntyre JA, Shade SB. High HIV incidence in a South African community of men who have sex with men: results from the Mpumalanga Men's Study, 2012-2015. *J Acquir Immun Defic Syndr* 2016; **73**:609-611.
123. Shahmanesh M, Patel V, Mabey D, Cowan F. Effectiveness of interventions for the prevention of HIV and other sexually transmitted infections in female sex workers in resource poor setting: a systematic review. *Trop Med Int Health* 2008; **13**:659-679.
124. Ghys PD, Diallo MO, Ettiègne-Traoré V, Kalé K, Tawil O, Caraël M, *et al.* Increase in condom use and decline in HIV and sexually transmitted diseases among female sex workers in Abidjan, Côte d'Ivoire, 1991-1998. *AIDS* 2002; **16**:251-258.
125. Cowan FM, Davey C, Fearon E, Mushati P, Dirawo J, Chabata S, *et al.* Targeted combination prevention to support female sex workers in Zimbabwe accessing and adhering to antiretrovirals for treatment and prevention of HIV (SAPPH-IRe): a cluster-randomised trial. *Lancet HIV* 2018; **5**:e417-426.
126. Bekker LG, Johnson L, Cowan F, Overs C, Besada D, Hillier S, *et al.* Combination HIV prevention for female sex workers: what is the evidence? *Lancet* 2015; **385**:72-87.
127. Lafort Y, Greener L, Lessitala F, Chabeda S, Greener R, Beksinska M, *et al.* Effect of a 'diagonal' intervention on uptake of HIV and reproductive health services by female sex workers in three sub-Saharan African cities. *Trop Med Int Health* 2018; **23**:774-784.
128. Chow EP, Tung K, Tucker JD, Muessig KE, Su S, Zhang X, *et al.* Behavioral interventions improve condom use and HIV testing uptake among female sex workers in China: a systematic review and meta-analysis. *AIDS Patient Care STDs* 2015; **29**:454-460.
129. Lawrence E, Struthers P, van Hove G. HIV counselling and testing in secondary schools: What students want. *South Afr J HIV Med* 2016; **16**:390.
130. Pfaff C, de Beer J. Expanding access to HIV counselling and testing at schools - the Manguzi experience. *South Afr J HIV Med* 2011; **12**:16-18.
131. Madiba S, Mokgatle M. "Students want HIV testing in schools" a formative evaluation of the acceptability of HIV testing and counselling at schools in Gauteng and North West provinces in South Africa. *BMC Public Health* 2015; **15**:388.
132. Meinck F, Carty C, Cluver L. Predictors for HIV testing in South African adolescents: schools-based testing, pregnancy and sexual experience. *INTEREST Workshop*. Yaoundé, Cameroon; 2016.
133. Corbett EL, Dauya E, Matambo R, Cheung YB, Makamure B, Bassett MT, *et al.* Uptake of workplace HIV counselling and testing: a cluster-randomised trial in Zimbabwe. *PLoS Med* 2006; **3**:e238.
134. Van der Borght SF, Schim van der Loeff MF, Clevenbergh P, Kabarega JP, Kamo E, van Cranenburgh K, *et al.* Long-term voluntary counseling and testing (VCT) uptake dynamics in a multicountry HIV workplace program in sub-Saharan Africa. *AIDS Care* 2010; **22**:195-205.

135. Kalibala S, Tun W, Cherutich P, Nganga A, Oweya E, Oluoch P. Factors associated with acceptability of HIV self-testing among health care workers in Kenya. *AIDS Behav* 2014; **18 (Suppl 4)**:S405-414.
136. Statistics South Africa. Quarterly Labour Force Survey, Quarter 1: 2018. Pretoria; 2018. Available: http://www.statssa.gov.za/publications/P0211/P02111stQuarter2018.pdf. Accessed 20 Dec 2018
137. Drake AL, Wagner A, Richardson B, John-Stewart G. Incident HIV during pregnancy and postpartum and risk of mother-to-child HIV transmission: a systematic review and meta-analysis. *PLoS Med* 2014; **11**:e1001608.
138. Johnson LF, Stinson K, Newell ML, Bland RM, Moultrie H, Davies MA, *et al.* The contribution of maternal HIV seroconversion during late pregnancy and breastfeeding to mother-to-child transmission of HIV. *J Acquir Immun Defic Syndr* 2012; **59**:417-425.
139. Katz DA, Kiarie JN, John-Stewart GC, Richardson BA, John FN, Farquhar C. Male perspectives on incorporating men into antenatal HIV counseling and testing. *PLoS One* 2009; **4**:e7602.
140. Jefferys LF, Nchimbi P, Mbezi P, Sewangi J, Theuring S. Official invitation letters to promote male partner attendance and couple voluntary HIV counselling and testing in antenatal care: an implementation study in Mbeya Region, Tanzania. *Reproductive Health* 2015; **12**:95.
141. Krakowiak D, Kinuthia J, Osoti AO, Asila V, Gone MA, Mark J, *et al.* Home-based HIV testing among pregnant couples increases partner testing and identification of serodiscordant partnerships. *Journal of Acquired Immune Deficiency Syndrome* 2016; **72 (Suppl 2)**:S167-173.
142. Osoti AO, John-Stewart G, Kiarie J, Richardson B, Kinuthia J, Krakowiak D, *et al.* Home visits during pregnancy enhance male partner HIV counselling and testing in Kenya: a randomized clinical trial. *AIDS* 2014; **28**:95-103.
143. Byamugisha R, Åstrøm AN, Ndeezi G, Karamagi CA, Tylleskär T, Tumwine JK. Male partner antenatal attendance and HIV testing in eastern Uganda: a randomized facility-based intervention trial. *J Int AIDS Soc* 2011; **14**:43.
144. Mohlala BK, Boily MC, Gregson S. The forgotten half of the equation: randomized controlled trial of a male invitation to attend couple voluntary counselling and testing. *AIDS* 2011; **25**:1535-1541.
145. Masters SH, Agot K, Obonyo B, Napierala Mavedzenge S, Maman S, Thirumurthy H. Promoting partner testing and couples testing through secondary distribution of HIV self-tests: A randomized clinical trial. *PLoS Med* 2016; **13**:e1002166.
146. Carballo-Diéguez A, Frasca T, Balan I, Ibitoye M, Dolezal C. Use of a rapid HIV home test prevents HIV exposure in a high risk sample of men who have sex with men. *AIDS Behav* 2012; **16**:1753-1760.
147. Lippman SA, Lane T, Rabede O, Gilmore H, Chen YH, Mlotshwa N, *et al.* High acceptability and increased HIV testing frequency following introduction of HIV self-testing and network distribution among South African MSM. *Journal of Acquired Immune Deficiency Syndrome* 2018; **77**:279-287.
148. Choko AT, Desmond N, Webb EL, Chavula K, Napierala-Mavedzenge S, Gaydos CA, *et al.* The uptake and accuracy of oral kits for HIV self-testing in high HIV prevalence setting: a cross-sectional feasibility study in Blantyre, Malawi. *PLoS Med* 2011; **8**:e1001102.

149. Ayles H, Floyd S, Mulubwa C, Hensen B, Schaap A, Phiri M, *et al.* Increasing knowledge of HIV status among men: a cluster-randomised trial of community-based distribution of oral HIV self-test kits nested in four HPTN 071 communities in Zambia [Abstract TUAC0406LB]. *9th International AIDS Society Conference*. Paris, France; 2017.
150. Pettifor A, Kahn K, Kimaru L, Myakayaka Z, Selin A, Haber N, *et al.* HIV self-testing increases testing in young South African women: results of an RCT [Abstract 992]. *25th Conference on Retroviruses and Opportunistic Infections*. Boston, USA; 2018.
151. Chanda MM, Ortblad KF, Mwale M, Chongo S, Kanchele C, Kamungoma N, *et al.* HIV self-testing among female sex workers in Zambia: A cluster randomized controlled trial. *PLoS Med* 2017; **14**:e1002442.
152. Ortblad K, Kibuuka Musoke D, Ngabirano T, Nakitende A, Magoola J, Kayiira P, *et al.* Direct provision versus facility collection of HIV self-tests among female sex workers in Uganda: A cluster-randomized controlled health systems trial. *PLoS Med* 2017; **14**:e1002458.
153. Mavedzenge S, Sibanda E, Dirawo J, Hatzold K, Mugurungi O, Cowan F. Feasibility of HIV self-test programming among female sex workers in Zimbabwe [Abstract MOAX0104]. *9th International AIDS Society Conference*. Paris, France; 2017.
154. Ngure K, Heffron R, Mugo N, Irungu E, Njuguna N, Mwaniki L, *et al.* Uptake of self-testing among people receiving PrEP in Kenya. *Research for Prevention Conference*. Cape Town, South Africa; 2014.
155. Johnson CC, Kennedy C, Fonner V, Siegfried N, Figueroa C, Dalal S, *et al.* Examining the effects of HIV self-testing compared to standard HIV testing services: a systematic review and meta-analysis. *J Int AIDS Soc* 2017; **20**:21594.
156. Mugo PM, Micheni M, Shangala J, Hussein MH, Graham SM, Rinke de Wit TF, *et al.* Uptake and acceptability of oral HIV self-testing among community pharmacy clients in Kenya: A feasibility study. *PLoS One* 2017; **12**:e0170868.
157. Marlin RW, Young SD, Bristow CC, Wilson G, Rodriguez J, Ortiz J, *et al.* Piloting an HIV self-test kit voucher program to raise serostatus awareness of high-risk African Americans, Los Angeles. *BMC Public Health* 2014; **14**:1226.
158. Tan WS, Chow EP, Fairley CK, Chen MY, Bradshaw CS, Read TR. Sensitivity of HIV rapid tests compared with fourth-generation enzyme immunoassays or HIV RNA tests. *AIDS* 2016; **30**:1951-1960.
159. Bock P, Phiri C, Piwowar-Manning E, Kosloff B, Mandla N, Young A, *et al.* Understanding low sensitivity of community-based HIV rapid testing: experiences from the HPTN 071 (PopART) trial in Zambia and South Africa. *J Int AIDS Soc* 2017; **20**:21780.
160. Kufa T, Kharsany AB, Cawood C, Khanyile D, Lewis L, Grobler A, *et al.* Misdiagnosis of HIV infection during a South African community-based survey: implications for rapid HIV testing. *J Int AIDS Soc* 2017; **20 (Suppl 6)**:21753.
161. Jackson D, Naik R, Tabana H, Pillay M, Madurai S, Zembe W, *et al.* Quality of home-based rapid HIV testing by community lay counsellors in a rural district of South Africa. *J Int AIDS Soc* 2013; **16**:18744.
162. Figueroa C, Johnson C, Ford N, Sands A, Dalal S, Meurant R, *et al.* Reliability of HIV rapid diagnostic tests for self-testing compared with testing by health-care workers: a systematic review and meta-analysis. *Lancet HIV* 2018; **5**:e277-290.

163. Kranzer K, Zeinecker J, Ginsberg P, Orrell C, Kalawe NN, Lawn SD, *et al.* Linkage to HIV care and antiretroviral therapy in Cape Town, South Africa. *PLoS One* 2010; **5**:e13801.
164. Rosen S, Fox MP. Retention in HIV care between testing and treatment in sub-Saharan Africa: a systematic review. *PLoS Med* 2011; **8**:e1001056.
165. Stevens WS, Gous NM, MacLeod WB, Long LC, Variava E, Martinson NA, *et al.* Multidisciplinary point-of-care testing in South African primary health care clinics accelerates HIV ART initiation but does not alter retention in care. *Journal of Acquired Immune Deficiency Syndrome* 2017; **76**:65-73.
166. Schwartz S, Lambert A, Phaswana-Mafuya N, Kose Z, McIngana M, Holland C, *et al.* Engagement in the HIV care cascade and barriers to antiretroviral therapy uptake among female sex workers in Port Elizabeth, South Africa: findings from a respondent-driven sampling study. *Sex Transm Infect* 2017; **93**:290-296.
167. Chanda M, Ortblad K, Mwale M, Chongo S, Kanchele C, Kamungoma N, *et al.* HIV self-testing among female sex workers in Zambia: a randomized controlled trial [Abstract MOAX0105LB]. *9th Internal AIDS Society Conference*. Paris, France; 2017.
168. MacKellar DA, Williams D, Storer N, Okello V, Azih C, Drummond J, *et al.* Enrollment in HIV care two years after HIV diagnosis in the kingdom of Swaziland: An evaluation of a national program of new linkage procedures. *PLoS One* 2016; **11**:e0150086.
169. Tumwesigye E, Wana G, Kasasa S, Muganzi E, Nuwaha F. High uptake of home-based, district-wide, HIV counseling and testing in Uganda. *AIDS Patient Care STDs* 2010; **24**:735-741.
170. Ruzagira E, Grosskurth H, Kamali A, Baisley K. Brief counselling after home-based HIV counselling and testing strongly increases linkage to care: a cluster-randomized trial in Uganda. *J Int AIDS Soc* 2017; **20**.
171. Dorward J, Mabuto T, Charalambous S, Fielding KL, Hoffmann CJ. Factors associated with poor linkage to HIV care in South Africa: secondary analysis of data from the Thol'impilo trial. *Journal of Acquired Immune Deficiency Syndrome* 2017; **76**:453-460.
172. van Rooyen H, Barnabas RV, Baeten JM, Phakathi Z, Joseph P, Krows M, *et al.* High HIV testing uptake and linkage to care in a novel program of home-based HIV counseling and testing with facilitated referral in KwaZulu-Natal, South Africa. *J Acquir Immun Defic Syndr* 2013; **64**:e1-8.
173. Labhardt ND, Ringera I, Lejone TI, Klimkait T, Muhairwe J, Amstutz A, *et al.* Effect of offering same-day ART vs usual health facility referral during home-based HIV testing on linkage to care and viral suppression among adults with HIV in Lesotho: the CASCADE randomized clinical trial. *JAMA* 2018; **319**:1103-1112.
174. Plazy M, Farouki KE, Iwuji C, Okesola N, Orne-Gliemann J, Larmarange J, *et al.* Access to HIV care in the context of universal test and treat: challenges within the ANRS 12249 TasP cluster-randomized trial in rural South Africa. *J Int AIDS Soc* 2016; **19**:20913.
175. South African Reserve Bank. Mid-year average USD-ZAR conversion rates. 2017. Available: https://www.resbank.co.za/publications/detail-item-view/pages/publications.aspx?sarbweb=3b6aa07d-92ab-441f-b7bf-bb7dfb1bedb4&sarblist=21b5222e-7125-4e55-bb65-56fd3333371e&sarbitem=7921. Accessed 20 Aug 2017

176. Murray CJ. Quantifying the burden of disease: the technical basis for disability-adjusted life years. *Bull WHO* 1994; **72**:429-445.
177. Fox MP, Maskew M, MacPhail AP, Long L, Brennan AT, Westreich D, *et al.* Cohort profile: the Themba Lethu Clinical Cohort, Johannesburg, South Africa. *Int J Epidemiol* 2013; **42**:430-439.
178. Sweeney S, Obure CD, Terris-Prestholt F, Darsamo V, Michaels-Igbokwe C, Muketo E, *et al.* The impact of HIV/SRH service integration on workload: analysis from the Integra Initiative in two African settings. *Human Resoures for Health* 2014; **12**:42.
179. Statistics South Africa. Poverty Profile of South Africa: Application of the poverty lines on the LCS 2008/2009. Pretoria; 2012.
180. Department of Health. Guidelines for the management of Tuberculosis, Human Immunodeficiency Virus and Sexually-Transmitted Infections in Correctional Centres Pretoria; 2013.
181. Department of Basic Education. National Education Policy Act, 1996 (Act No 27 of 1996)- Call for Written Submissions from Stakeholder Bodies and Members of the Public on Department of Basic Education Draft National Policy on HIV, STIs And TB. Notice 395 of 2015 Government Gazette 38763;3-35.; 2015.
182. Department of Correctional Services. Annual report for the 2003/04 financial year. 2004. Available: http://www.dcs.gov.za/Publications/AnnualReports.aspx. Accessed 14 Oct 2016
183. Parry CD, Pluddemann A, Louw A, Leggett T. The 3-metros study of drugs and crime in South Africa: findings and policy implications. *American Journal of Drug and Alcohol Abuse* 2004; **30**:167-185.
184. Meyer-Rath G, van Rensburg C, Larson B, Jamieson L, Rosen S. Revealed willingness-to-pay versus standard cost-effectiveness thresholds: Evidence from the South African HIV Investment Case. *PLoS One* 2017; **12**:e0186496.
185. Garnett G. An introduction to mathematical models in sexually transmitted disease epidemiology. *Sex Transm Infect* 2002; **78**:7-12.
186. U.S. President's Emergency Plan for AIDS Relief. PEPFAR 2019 Country Operational Plan Guidance for all PEPFAR Countries. 2019. Available: https://www.pepfar.gov/documents/organization/288160.pdf. Accessed 20 Feb 2019
187. Korenromp E, Stover J. HIV testing: program pathways for scale-up to the 90% knowledge target - epidemiological projections and country typologies. 2015.
188. Cambiano V, Ford D, Mabugu T, Napierala Mavedzenge S, Miners A, Mugurungi O, *et al.* Assessment of the potential impact and cost-effectiveness of self-testing for HIV in low-income countries. *J Infect Dis* 2015; **212**:570-577.
189. Bassett IV, Govindasamy D, Erlwanger AS, Hyle EP, Kranzer K, van Schaik N, *et al.* Mobile HIV screening in Cape Town, South Africa: clinical impact, cost and cost-effectiveness. *PLoS One* 2014; **9**:e85197.
190. Ying R, Sharma M, Celum C, Baeten JM, van Rooyen H, Hughes JP, *et al.* Home testing and counselling to reduce HIV incidence in a generalised epidemic setting: a mathematical modelling analysis. *Lancet HIV* 2016; **3**:e275-282.
191. Olney JJ, Braitstein P, Eaton JW, Sang E, Nyambura M, Kimaiyo S, *et al.* Evaluating strategies to improve HIV care outcomes in Kenya: a modelling study. *Lancet HIV* 2016; **3**:e592-e600.

Annex 1: Average cost and cost per category by testing modality

"%" denotes the percent of the population that this cost applies to.

Partner testing

Average test cost	Staff	Equipment	Consumables	Overheads	Total	%
Positive test	3.44	0.01	2.89	0.24	6.59	7%
Negative test	2.18	0.01	2.32	0.22	4.73	93%
Average all test	2.27	0.01	2.36	0.22	4.86	100%
**Cost per category**	**Staff**	**Equipment**	**Consumables**	**Overheads**	**Total**	**%**
Arrival/registration	0.60	0.01	0.37	0.20	1.18	100%
Information session	0.32	-	-	-	0.32	100%
Pre-test counselling	0.63	-	0.04	0.01	0.69	100%
Test 1	0.32	0.00	0.94	0.01	1.27	100%
Test 2	0.32	-	0.55	-	0.05	6%
Test 3	0.32	-	0.53	-	0.01	1%
Test 4	0.32	-	0.55	-	0.01	1%
Whole blood draw	0.44	0.00	3.18	0.02	0.00	0.02%
Post-test counselling - Positive	1.26	-	0.98	0.02	2.26	100%
Negative result counselling	0.32	-	0.97	-	1.28	100%

Testing of STI patients

Average test cost	Staff	Equipment	Consumables	Overheads	Total	%
Positive test	2.94	0.01	2.76	0.22	5.92	8%
Negative test	1.55	0.01	2.19	0.20	3.94	92%
Average all test	1.66	0.01	2.23	0.20	4.09	100%
**Cost per category**	**Staff**	**Equipment**	**Consumables**	**Overheads**	**Total**	**%**
Arrival/registration	0.60	0.01	0.37	0.20	1.18	100%
Information session	0.32	-	-	-	0.32	100%
Pre-test counselling	0.13	0.00	0.04	-	0.17	100%
Test 1	0.32	-	0.80	-	1.12	100%
Test 2	0.32	-	0.54	-	0.14	16%
Test 3	0.32	-	0.52	-	0.01	1%
Test 4	0.32	-	0.54	-	0.01	1%
Whole blood draw	0.44	0.00	3.17	0.02	0.00	0%
Post-test counselling - Positive	1.26	-	0.98	0.02	2.26	100%
Negative result counselling	0.19	-	0.97	-	1.16	100%

Antenatal clinic clients testing*

Average test cost	Staff	Equipment	Consumables	Overheads	Total	%
Positive test	2.94	0.01	2.75	0.24	5.94	3%
Negative test	1.55	0.01	2.19	0.22	3.97	97%

Average all test	1.60	0.01	2.21	0.22	4.04	100%
**Cost per category**	**Staff**	**Equipment**	**Consumables**	**Overheads**	**Total**	**%**
Arrival/registration	0.60	0.01	0.37	0.20	1.18	100%
Information session	0.32	-	-	-	0.32	100%
Pre-test counselling	0.13	-	0.04	0.01	0.18	100%
Test 1	0.32	0.00	0.80	0.01	1.13	100%
Test 2	0.32	-	0.54	-	0.05	6%
Test 3	0.32	-	0.52	-	0.01	1%
Test 4	0.32	-	0.54	-	0.01	1%
Whole blood draw	0.44	0.00	3.17	0.02	0.00	0%
Post-test counselling - Positive	1.26	-	0.98	0.02	2.26	100%
Negative result counselling	0.19	-	0.97	-	1.16	100%

* Note that this includes the costs of retesting in late pregnancy, for women who test negative at their first antenatal visit.

OI patients

**Average test cost**	**Staff**	**Equipment**	**Consumables**	**Overheads**	**Total**	**%**
Positive test	2.94	0.01	2.75	0.22	5.92	23%
Negative test	1.55	0.01	2.18	0.20	3.93	77%
Average all test	1.88	0.01	2.32	0.20	4.40	100%
**Cost per category**	**Staff**	**Equipment**	**Consumables**	**Overheads**	**Total**	**%**
Arrival/registration	0.60	0.01	0.37	0.20	1.18	100%
Information session	0.32	-	-	-	0.32	100%
Pre-test counselling	0.13	-	0.04	-	0.17	100%
Test 1	0.32	-	0.80	-	1.11	100%
Test 2	0.32	-	0.55	-	0.06	7%
Test 3	0.32	0.00	0.53	0.01	0.01	1%
Test 4	0.32	0.00	0.55	0.06	0.01	1%
Whole blood draw	0.44	0.00	3.18	0.02	0.00	0%
Post-test counselling - Positive	1.26	-	0.98	0.02	2.26	100%
Negative result counselling	0.19	0.00	0.97	0.00	1.16	100%

Men seeking MMC

**Average test cost**	**Staff**	**Equipment**	**Consumables**	**Overheads**	**Total**	**%**
Positive test	2.94	0.01	2.75	0.22	5.91	2%
Negative test	1.55	0.01	2.19	0.20	3.94	98%
Average all test	1.58	0.01	2.20	0.20	3.99	100%
**Cost per category**	**Staff**	**Equipment**	**Consumables**	**Overheads**	**Total**	**%**
Arrival/registration	0.60	0.01	0.37	0.20	1.18	100%
Information session	0.32	-	-	-	0.32	100%
Pre-test counselling	0.13	0.00	0.04	-	0.17	100%
Test 1	0.32	-	0.80	-	1.12	100%

Test 2	0.32	-	0.54	-	0.02	2%
Test 3	0.32	-	0.52	-	0.00	1%
Test 4	0.32	-	0.52	-	0.00	1%
Whole blood draw	0.44	0.00	3.17	0.02	0.00	0%
Post-test counselling - Positive	1.26	-	0.98	0.02	2.26	100%
Negative result counselling	0.19	-	0.97	-	1.16	100%

PrEP clients

Average test cost	Staff	Equipment	Consumables	Overheads	Total	%
Positive test	2.94	0.01	2.75	0.22	5.92	2%
Negative test	1.55	0.01	2.19	0.20	3.94	98%
Average all test	1.57	0.01	2.20	0.20	3.97	100%
**Cost per category**	**Staff**	**Equipment**	**Consumables**	**Overheads**	**Total**	**%**
Arrival/registration	0.60	0.01	0.37	0.20	1.18	100%
Information session	0.32	-	-	-	0.32	100%
Pre-test counselling	0.13	0.00	0.04	-	0.17	100%
Test 1	0.32	-	0.80	-	1.12	100%
Test 2	0.32	-	0.54	-	0.03	3%
Test 3	0.32	-	0.52	-	0.00	1%
Test 4	0.73	-	0.54	-	0.01	1%
Whole blood draw	0.44	0.00	3.17	0.02	0.00	0%
Post-test counselling - Positive	1.26	0.00	0.98	0.02	2.26	100%
Negative result counselling	0.19	-	0.97	-	1.16	100%

Prisoners

Average test cost	Staff	Equipment	Consumables	Overheads	Total	%
Positive test	4.70	0.00	2.78	0.02	7.50	3%
Negative test	2.88	0.00	2.22	0.00	5.10	97%
Average all test	2.94	0.00	2.24	0.00	5.18	100%
**Cost per category**	**Staff**	**Equipment**	**Consumables**	**Overheads**	**Total**	**%**
Arrival/registration	0.60	-	0.37	-	0.98	100%
Information session	0.45	-	-	-	0.45	100%
Pre-test counselling	0.91	-	-	-	0.91	100%
Test 1	0.45	-	0.88	-	1.33	100%
Test 2	0.45	-	0.54	-	0.06	6%
Test 3	0.45	-	0.52	-	0.01	1%
Test 4	0.45	-	0.54	-	0.01	1%
Whole blood draw	0.52	0.00	3.17	0.02	0.00	0%
Post-test counselling - Positive	1.82	-	0.98	0.02	2.81	100%
Negative result counselling	0.45	0.00	0.97	0.00	1.42	100%

General testing

Average test cost	Staff	Equipment	Consumables	Overheads	Total	%
Positive test	3.44	0.01	2.75	0.22	6.42	4%
Negative test	2.18	0.01	2.19	0.20	4.57	96%
Average all test	2.23	0.01	2.21	0.20	4.64	100%
**Cost per category**	**Staff**	**Equipment**	**Consumables**	**Overheads**	**Total**	**%**
Arrival/registration	0.60	0.01	0.37	0.20	1.18	100%
Information session	0.32	-	-	-	0.32	100%
Pre-test counselling	0.63	0.00	0.04	-	0.68	100%
Test 1	0.32	-	0.80	-	1.12	100%
Test 2	0.32	-	0.54	-	0.02	2%
Test 3	0.32	-	0.52	-	0.00	1%
Test 4	0.32	0.00	0.54	0.06	0.01	1%
Whole blood draw	0.19	0.00	3.17	0.02	0.00	0%
Post-test counselling - Positive	1.26	-	0.98	0.02	2.26	100%
Negative result counselling	0.32	-	0.97	-	1.28	100%

Home-based HCT (urban)

Average test cost	Staff	Equipment	Consumables	Overheads	Total	%
Positive test	2.12	0.01	2.39	0.23	4.74	6%
Negative test	2.12	0.01	1.82	0.23	4.17	94%
Average all test	2.12	0.01	1.85	0.23	4.21	100%
**Cost per category**	**Staff**	**Equipment**	**Consumables**	**Overheads**	**Total**	**%**
Arrival/registration	2.12	-	0.00	0.19	2.31	100%
Pre-test counselling	-	0.01	0.04	0.04	0.09	100%
Test 1	-	-	0.80	-	0.80	100%
Test 2	-	-	0.55	-	0.03	5.59%
Test 3	-	-	0.53	-	0.00	1%
Test 4	-	-	0.55	-	0.00	1%
Whole blood draw	-	-	3.18	-	0.00	0%
Post-test counselling - Positive	-	-	0.98	-	0.98	100%
Negative result counselling	-	-	0.97	-	0.97	100%

Home-based HCT (rural)

Average test cost	Staff	Equipment	Consumables	Overheads	Total	%
Positive test	3.39	0.02	2.38	0.36	6.15	6%
Negative test	3.39	0.02	1.82	0.36	5.59	94%
Average all test	3.39	0.02	1.85	0.36	5.62	100%
**Cost per category**	**Staff**	**Equipment**	**Consumables**	**Overheads**	**Total**	**%**
Arrival/registration	3.39	-	-	0.30	3.69	100%
Pre-test counselling	-	0.02	0.04	0.06	0.12	100%
Test 1	-	-	0.80	-	0.80	100%

Test 2	-	-	0.54	-	0.03	6%
Test 3	-	-	0.52	-	0.00	1%
Test 4	-	-	0.54	-	0.00	1%
Whole blood draw	-	-	3.17	-	0.00	0%
Post-test counselling - Positive	-	-	0.98	-	0.98	100%
Negative result counselling	-	-	0.97	-	0.97	100%

Mobile testing (rural)

Average test cost	Staff	Equipment	Consumables	Overheads	Total	%
Positive test	5.13	0.00	2.45	0.05	7.63	6%
Negative test	5.13	0.00	0.92	0.05	6.10	94%
Average all test	5.13	0.00	1.02	0.05	6.20	100%
**Cost per category**	**Staff**	**Equipment**	**Consumables**	**Overheads**	**Total**	**%**
Arrival/registration	1.67	-	-	0.04	1.71	100%
Pre-test counselling	2.01	0.00	0.04	0.018025348	2.07	100%
Test 1	1.45	-	0.88	-	2.33	100%
Test 2	-	-	0.54	-	0.03	6%
Test 3	-	-	0.52	-	0.00	1%
Test 4	-	-	0.54	-	0.00	1%
Whole blood draw	-	-	3.17	-	0.00	0%
Post-test counselling - Positive	-	-	0.98	-	0.98	100%
Negative result counselling	-	-	-	-	-	100%

Mobile testing (urban)

Average test cost	Staff	Equipment	Consumables	Overheads	Total	%
Positive test	3.20	0.00	2.45	0.03	5.69	6%
Negative test	3.20	0.00	0.92	0.03	4.16	94%
Average all test	3.20	0.00	1.02	0.03	4.26	100%
**Cost per category**	**Staff**	**Equipment**	**Consumables**	**Overheads**	**Total**	**%**
Arrival/registration	1.05	-	-	0.02	1.07	100%
Pre-test counselling	1.25	0.00	0.04	0.01	1.31	100%
Test 1	0.90	-	0.88	-	1.78	100%
Test 2	-	-	0.54	-	0.03	6%
Test 3	-	-	0.52	-	0.00	1%
Test 4	-	-	0.54	-	0.00	1%
Whole blood draw	-	-	3.17	-	0.00	0%
Post-test counselling - Positive	-	-	0.98	-	0.98	100%
Negative result counselling	-	-	-	-	-	100%

MSM testing

Average test cost	Staff	Equipment	Consumables	Overheads	Demand	Targeting	Total	%

Positive test	2.00	0.00	2.17	0.29	0.02	0.16	4.64	15%
Negative test	2.32	0.00	1.89	0.29	0.02	0.16	4.68	85%
Average all test	2.27	0.00	1.93	0.29	0.02	0.16	4.67	100%

**Cost per category**	**Staff**	**Equipment**	**Consumables**	**Overheads**	**Total**	**%**
Arrival/registration	2.00	-	-	0.19	2.19	100%
Pre-test counselling	-	0.00	0.04	0.10	-	0%
Test 1	-	-	0.88	-	0.88	100%
Test 2	-	-	0.54	-	0.03	6%
Test 3	-	-	0.52	-	0.01	1%
Test 4	-	-	0.54	-	0.01	1%
Whole blood draw	-	-	3.17	-	-	0%
Post-test counselling - Positive	-	-	0.69	-	0.69	100%
Negative result counselling	0.32	-	0.97	-	1.28	100%

FSW testing

**Average test cost**	**Staff**	**Equipment**	**Consumables**	**Overheads**	**Targeting**	**Total**	**%**
Positive test	3.03	0.00	2.46	0.04	0.64	6.17	18%
Negative test	3.03	0.00	0.92	0.04	0.64	4.64	82%
Average all test	3.03	0.00	1.21	0.04	0.64	4.92	100%

**Cost per category**	**Staff**	**Equipment**	**Consumables**	**Overheads**	**Total**	**%**
Targeting				0.64		100%
Arrival/registration	1.33	-	-	0.03	1.36	100%
Pre-test counselling	0.42	0.00	0.04	0.01	0.48	100%
Test 1	1.28	-	0.88	-	2.16	100%
Test 2	-	-	0.54	-	0.03	6%
Test 3	-	-	0.52	-	0.01	1%
Test 4	-	-	0.54	-	0.01	1%
Whole blood draw	-	-	3.17	-	0.00	0%
Post-test counselling - Positive	-	-	0.98	-	0.98	100%
Negative result counselling	-	-	-	-	-	100%

Workplace testing

**Average test cost**	**Staff**	**Equipment**	**Consumables**	**Overheads**	**Targeting**	**Total**	**%**
Positive test	2.25	-	1.41	0.13	0.38	4.15	8%
Negative test	2.25	-	0.85	0.13	0.38	3.60	92%
Average all test	2.25	-	0.89	0.13	0.38	3.64	100%

**Cost per category**	**Staff**	**Equipment**	**Consumables**	**Overheads**	**Total**	**%**
Targeting				0.375026465		100%
Arrival/registration	2.25	-	-	0.13	2.37	100%

Pre-test counselling	-	-	0.04	-	0.04	100%
Test 1	-	-	0.80	-	0.80	100%
Test 2	-	-	0.54	-	0.03	6%
Test 3	-	-	0.52	-	0.01	1%
Test 4	-	-	0.54	-	0.01	1%
Whole blood draw	-	-	3.17	-	0.00	0%
Post-test counselling - Positive	-	-	-	-	-	100%
Negative result counselling	-	-	-	-	-	100%

Mobile testing plus community mobilisation

Average test cost	Staff	Equipment	Consumables	Overheads	Demand	Total	%
Positive test	5.13	-	1.40	0.04	11.41	17.98	6%
Negative test	5.13	-	0.85	0.04	11.41	17.43	94%
Average all test	5.13	-	0.88	0.04	11.41	17.46	100%

Cost per category	Staff	Equipment	Consumables	Overheads	Total	%
Demand Creation				20.95		100%
Arrival/registration	1.67	-	-	0.04	1.71	100%
Pre-test counselling	2.01	-	0.04	-	2.05	100%
Test 1	1.45	-	0.80	-	2.25	100%
Test 2	-	-	0.54	-	0.03	6%
Test 3	-	-	0.52	-	0.00	1%
Test 4	-	-	0.54	-	0.00	1%
Whole blood draw	-	-	3.17	-	0.00	0%
Post-test counselling - Positive	-	-	-	-	-	100%
Negative result counselling	-	-	-	-	-	100%

School testing

Average test cost	Staff	Equipment	Consumables	Overheads	Total	%
Positive test	5.13	0.00	2.45	0.05	7.63	2%
Negative test	5.13	0.00	1.89	0.05	7.07	98%
Average all test	5.13	0.00	1.90	0.05	7.08	100%
**Cost per category**	**Staff**	**Equipment**	**Consumables**	**Overheads**	**Total**	**%**
Arrival/registration	1.67	-	-	0.04	1.71	100%
Pre-test counselling	2.01	0.00	0.04	0.02	2.07	100%
Test 1	1.45	-	0.88	-	2.33	100%
Test 2	-	-	0.54	-	0.03	6%
Test 3	-	-	0.52	-	0.00	1%
Test 4	-	-	0.54	-	0.00	1%
Whole blood draw	-	-	3.17	-	0.00	0%
Post-test counselling - Positive	-	-	0.98	-	0.98	100%

Negative result counselling	-	-	-	-	-	

Family planning clients

Average test cost	Staff	Equipment	Consumables	Overheads	Total	%
Positive test	2.94	0.01	2.76	0.28	5.99	11%
Negative test	1.55	0.01	1.90	0.26	3.72	89%
Average all test	1.70	0.01	2.00	0.26	3.97	100%
**Cost per category**	**Staff**	**Equipment**	**Consumables**	**Overheads**	**Total**	**%**
Arrival/registration	0.60	0.01	0.37	0.20	1.18	100%
Information session	0.32	0.00	-	0.06	0.38	100%
Pre-test counselling	0.13	0.00	0.04	-	0.17	98%
Test 1	0.32	-	0.80	-	1.12	96%
Test 2	0.32	-	0.54	-	0.86	23%
Test 3	0.32	-	0.52	-	0.84	1%
Test 4	0.32	-	0.54	-	0.86	1%
Whole blood draw	0.44	0.00	3.17	0.02	3.63	0%
Post-test counselling - Positive	1.26	-	0.98	0.02	2.26	100%
Negative result counselling	0.19	-	0.68	-	0.87	100%

Assisted partner notification

Average test cost	Staff	Equipment	Consumables	Overheads	Demand	Total	%
Positive test	3.45	0.00	2.75	0.05	2.40	8.65	8%
Negative test	2.18	0.00	2.19	0.04	2.40	6.81	92%
Average all test	2.28	0.00	2.23	0.04	2.40	6.95	100%

Cost per category	Staff	Equipment	Consumables	Overheads	Total	%
Contract notification (self-referral, else tracing)	1.5	-	0.10	-	1.60	100%
Partner notification by call	0.1	-	0.70	-	0.80	100%
Arrival/registration	0.60	0.00	0.37	0.04	1.02	100%
Information session	0.32	-	-	-	0.32	100%
Pre-test counselling	0.63	0.00	0.04	-	0.67	98%
Test 1	0.32	-	0.80	-	1.12	96%
Test 2	0.32	-	0.54	-	0.86	12%
Test 3	1.23	0.00	0.52	0.01	1.77	1%
Test 4	0.32	-	0.54	-	0.86	1%
Whole blood draw	0.44	0.00	3.17	0.00	3.61	0%
Post-test counselling - Positive	1.26	-	0.98	0.00	2.24	100%
Negative result counselling	0.32	-	0.97	-	1.28	100%

ANC partners testing

Average test cost	Staff	Equipment	Consumables	Overheads	Demand	Total	%
Positive test	2.94	0.01	2.75	0.22	1.00	6.91	3%
Negative test	1.55	0.20	1.22	1.16	1.00	5.13	97%
Average all test	1.59	0.19	1.27	1.14	1.00	5.18	100%

Cost per category	Staff	Equipment	Consumables	Overheads	Total	%
Demand Creation				1.00	1.00	100%
Arrival/registration	0.60	0.01	0.37	0.20	1.18	100%
Information session	0.32	-	-	-	0.32	100%
Pre-test counselling	0.13	0.00	0.04	-	0.17	98%
Test 1	0.32	-	0.80	-	1.08	96%
Test 2	0.32	-	0.54	-	0.04	5%
Test 3	0.32	-	0.52	-	0.01	1%
Test 4	0.32	-	0.54	-	0.01	1%
Whole blood draw	0.44	0.00	3.17	0.02	0.00	0%
Post-test counselling - Positive	1.26	-	0.98	0.02	2.26	100%
Negative result counselling	0.19	0.19	0.00	0.97	0.01	100%

Home-based testing plus an offer of self-testing

Average test cost	Staff	Equipment	Consumables	Overheads	Demand	Total	%
Positive test	3.44	0.01	2.75	0.48	2.57	9.27	6%
Negative test	0.06	0.00	0.04	0.01	2.57	2.68	94%
Average all test	0.26	0.00	0.20	0.04	2.57	3.08	100%

Cost per category	Staff	Equipment	Consumables	Overheads	Total	%
Self-test kit and self-test demonstration*	-	-	-	2.57	2.57	100%
Arrival/registration†	0.60	0.01	0.37	0.29	1.28	2%
Information session†	0.32	0.00	-	0.10	0.41	2%
Pre-test counselling†	0.63	0.00	0.04	-	0.68	1%
Test 1†	0.32	-	0.80	-	1.12	0%
Test 2†	0.32	-	0.54	-	0.86	0%
Test 3†	0.32	-	0.52	-	0.84	1%
Test 4†	0.32	0.00	0.54	0.10	0.96	1%
Whole blood draw†	0.44	0.00	3.17	0.10	3.71	0%
Post-test counselling - Positive†	1.26	-	0.98	0.10	2.33	100%

* This includes the cost of the self-testing kit. † This cost only applies if the self-test returns a positive test and the individual seeks confirmatory testing.

ANC partners testing plus an offer of self-testing

Average test cost	Staff	Equipment	Consumables	Overheads	Demand	Total	%

Positive test	3.44	0.01	2.75	0.22	2.84	9.25	3%
Negative test	0.06	0.00	0.04	0.01	2.84	2.94	97%
Average all test	0.16	0.00	0.12	0.01	2.84	3.14	100%

**Cost per category**	**Staff**	**Equipment**	**Consumables**	**Overheads**	**Total**	**%**
Self-test kit and self-test demonstration*				2.84	2.84	100%
Arrival/registration†	0.60	0.00	0.37	0.05	0.02	2%
Information session†	0.32	-	-	-	0.01	2%
Pre-test counselling†	0.63	0.00	0.04	-	0.00	1%
Test 1†	0.32	-	0.80	-	0.00	0%
Test 2†	0.32	-	0.54	-	0.00	0%
Test 3†	0.32	-	0.52	-	0.00	0%
Test 4†	0.32	-	0.54	-	0.00	0%
Whole blood draw†	0.44	0.00	3.17	0.02	0.00	0%
Post-test counselling - Positive†	1.26	-	0.98	0.02	2.26	100%

* This includes the cost of the self-testing kit. † This cost only applies if the self-test returns a positive test and the individual seeks confirmatory testing.
